# Part I: NiMoO_4_ Nanostructures Synthesized by the Solution Combustion Method: A Parametric Study on the Influence of Synthesis Parameters on the Materials’ Physicochemical, Structural, and Morphological Properties

**DOI:** 10.3390/molecules27030776

**Published:** 2022-01-25

**Authors:** Mahmoud Bassam Rammal, Sasha Omanovic

**Affiliations:** Department of Chemical Engineering, McGill University, 3610 University Street, Montreal, QC H3A 0C5, Canada; sasha.omanovic@mcgill.ca

**Keywords:** nickel molybdate, nanostructures, solution combustion synthesis, agar, parametric study, physicochemical properties

## Abstract

The impact of process conditions on the synthesis of NiMoO_4_ nanostructures using a solution combustion synthesis (SCS) method, in which agar powder and Ni(NO_3_)_2_ were utilized as fuel and as the oxidant, respectively, was thoroughly studied. The results show that the calcination temperature had a significant implication on the specific surface area, phase composition, particle size, band gap, and crystallite size. The influence of calcination time on the resulting physicochemical/structural/morphological properties of NiMoO_4_ nanostructures was found to be a major effect during the first 20 min, beyond which these properties varied to a lesser extent. The increase in the Ni/Mo atomic ratio in the oxide impacted the combustion dynamics of the system, which led to the formation of higher surface area materials, with the prevalence of the β-phase in Ni-rich samples. Likewise, the change in the pH of the precursor solution showed that the combustion reaction is more intense in the high-pH region, entailing major implications on the physicochemical properties and phase composition of the samples. The change in the fuel content showed that the presence of agar is important, as it endows the sample with a fluffy, porous texture and is also vital for the preponderance of the β-phase.

## 1. Introduction

Mixed metal oxide compounds exhibit unique chemical and structural properties and are used extensively in a broad range of applications [1]. Nickel molybdates (NiMoO_4_) are an important family of mixed metal oxides due to their intriguing chemical and electronic properties, making them attractive catalytic materials. In fact, catalytic materials based on nickel molybdates have been extensively used in a wide range of industrial processes, namely in the chemical industry for the oxidative dehydrogenation of hydrocarbons, hydrodesulfurization, water–gas shift, and steam reforming, to name just a few [2]. Recently, a surge in interest has been observed for the utilization of this oxide family, produced at the nanoscale, in electrochemical applications (e.g., supercapacitors and lithium-ion batteries) after being long utilized in oxidative hydrogenation applications as bulk materials (commonly synthesized by coprecipitation) [2,3].

Many approaches have been adopted to produce NiMoO_4_ materials on the nanoscale and optimize their structure and physicochemical properties to enhance their performance in catalytic applications. The most commonly utilized methods that are reported in the literature include hydrothermal [4], sonochemical [5], mechanochemical [6], microwave [7], spray pyrolysis [8], solution combustion [9], and sol-gel [10]. The sol-gel method has been a popular wet-chemistry method because of its simplicity, low cost, and versatility [11]. However, classical sol-gel methods have often resulted in amorphous products, requiring extensive thermal treatment to obtain the desired physicochemical properties [11]. On the other hand, the solution combustion synthesis (SCS) method is an interesting alternative that enables the production of high-quality nanopowder in a simple, versatile manner and in a short time, with scale-up prospects [12]. Additionally, contrary to the frequently used solid-state synthesis methods that require a considerable input of energy, SCS can benefit from the intense release of heat, which allows fast in situ production of nanosized powders. SCS is characterized, among other things, by a highly exothermic combustion reaction and vigorous gas emission, both of which stem from a redox reaction between an organic fuel and oxidant [13]. Additionally, the final products of SCS could possess interesting properties such as abundant structural defects, which would prove useful in catalytic applications [14]. SCS is derived from the concepts of propellant chemistry and constitutes mixing component metal salts which contain anions of an oxidizing powder (typically metal nitrate) with an organic molecule of a reducing affinity, commonly known as fuel, followed by heating the mixture until a fast-propagating combustion reaction commences, resulting in the formation of oxide nanostructures. The first work on this method was carried out in 1988 by Patil et al. [15], who reported that during the production of alumina using Al(NO_3_)_3_ and urea, the precursor solution unexpectedly ignited upon heating, followed by foaming and a flame burst, generating a combustion reaction with a temperature of 1350 °C.

The use of SCS in the production of NiMoO_4_ has been limited thus far. Moreno et al. [9] investigated the production of nanometric NiMoO_4_, using metal acetylacetonate, urea (fuel), and ammonium nitrate (oxidant), reporting the occurrence of a high-energy exothermic reaction, which triggered the formation of the product. In another study, Selvan et al. [16] produced NiMoO_4_ using SCS by employing urea and metal nitrate as fuel and the oxidizing agent, respectively. Another group [17] utilized a method that closely resembles SCS (citrate-gel combustion) to produce NiO-MoO_3_ mixtures, using citric acid as fuel. In addition to the scarcity of the SCS-based systems for NiMoO_4_ production, the body of knowledge still lacks a systematic, parametric approach to evaluate the influence of experimental parameters on the physicochemical properties of materials produced by this method. This approach would help to consolidate a better fundamental understanding of the process and contribute to the development of advanced materials. That is because catalytic materials, among other things, are greatly influenced by the method of preparation since experimental conditions can impact an extensive range of properties [9]. The proper identification of the optimum experimental parameters in the synthesis procedure of a catalyst would contribute to cost reduction, a facile process, and enhanced products [18]. However, due to the extreme complexity, interrelation, and influence of the various parameters that control the process, a better understanding of the synthesis methodology would only be possible through a systematic research study, which evidences the effect of one variable while holding the other key parameters strictly constant.

As the physicochemical properties of the materials produced through SCS are strongly contingent on the employed experimental conditions, more fundamental research on this subject is warranted. Indeed, surface properties such as phase composition, particle size, porosity, surface area, morphology, aggregation, crystallinity, and optical properties are all contingent on the adopted experimental conditions, among which calcination temperature and time [19], the pH of the precursor solution [20], composition [11], and the fuel-to-oxidant ratio (φ) [21] are the major influential variables. In our previous preliminary work [22], we employed SCS using agar (C_14_H_24_O_9_) and Ni(NO_3_)_2_ as fuel and oxidant components, respectively. We noticed that after drying the precursor, and upon exposing the mixture to an elevated calcination temperature (500 °C) in a muffle furnace, a self-propagating, highly exothermic combustion reaction took place, followed by vigorous emission of gaseous products, producing foamy materials in a few seconds. The material was then calcined further for 6 h to improve the product properties and remove any possible impurities.

Herein, we extend on our previous study [22] by undertaking a comprehensive methodology to assess the influence of several synthesis parameters of interest, namely the calcination temperature and time, pH of the precursor solution, composition (Ni/Mo atomic ratio), and fuel content (i.e., fuel-to-oxidant ratio) described by the equivalence ratio (φ) on the physicochemical properties and microstructure of the synthesized materials. By relying on essential characterization techniques and first principle thermodynamic calculations, we aim to give insight into the interplay between the experimental conditions and the physicochemical properties of NiMoO_4_. The results presented here will also help the understanding of the role of experimental parameters of influence in SCS, in an effort to produce materials with properties that could be tailored for a wide range of catalytic applications where NiMoO_4_ finds uses.

Consequently, this paper presents the first part of a series of two papers, where the second paper (Part II) will discuss the influence of the parameters considered in this paper (synthesis parameters) and other non-synthesis parameters on the electrocatalytic properties of the produced nanostructured NiMo-oxides and their activity in the hydrogen evolution reaction.

## 2. Results and Discussion

### 2.1. Effect of Calcination Temperature

The effect of calcination temperature on the properties of oxide materials has been long studied. While some materials could be oxidized at relatively low calcination temperatures [23], others such as superconducting oxides must undergo a high-temperature calcination regimen (>1000 °C) in order to achieve the desired phases. For instance, Popa et al. [24] reported that pure, single-phase BiFeO_3_ nanocrystallites without impurities or amorphous phases were obtained when the precursor was treated at 600 °C for 3 h, while secondary phases were observed at 350 °C and 400 °C. D.W. Lee et al. [25] stated that below 850 °C, the production of BaCeO_3_ nanopowder using the citrate combustion process was accompanied by undesirable secondary phases. Only at 900 °C, single-phase BaCeO_3_ was obtained. Thomas et al. [26] prepared different tungsten-based oxide semiconductors using the SCS method, suggesting that the calcination temperature impacts the crystallite size, morphology, specific surface area, and photocatalytic properties.

The TGA/DSC analysis of NiMoO_4_ performed in our previous study [22] showed that a highly exothermic combustion reaction—a signature feature of SCS—occurred at 147 °C, manifested by a spark, followed by the appearance of an incalescent flame sweeping through the material and producing foamy materials in a few seconds. A calcination step is often required to remove residual impurities and attain the desirable phases. However, elevated calcination temperatures can also result in the reduction in the material’s surface area due to sintering, in addition to altering the phase composition, thereby affecting the activity of such a material in heterogeneous catalytic reactions, which depend strongly on the available surface area. Therefore, we prepared samples at different calcination temperatures to elucidate the effect of the calcination temperature on the microstructure and physicochemical properties of NiMoO_4_. The calcination time was fixed for 6 h in this section.

#### 2.1.1. XRD Measurements

NiMoO_4_ exhibits various polymorphs among which α-NiMoO_4_ and β-NiMoO_4_ are the most notable; α-NiMoO_4_ is considered as the room-temperature stable form of NiMoO_4_, while β-NiMoO_4_, being the metastable phase, has been traditionally obtained by heating α-NiMoO_4_ to elevated temperatures (>600 °C) and has been reported to remain stable only when kept above 180 °C [9]. Both phases belong to the monoclinic crystal system but have a different Mo coordination (octahedral for α-NiMoO_4_ and tetrahedral for β-NiMoO_4_). While α-NiMoO_4_ has been long synthesized by co-precipitation and solid-state methods, β-NiMoO_4_ has been rarely produced using conventional approaches [2]. The production of β-NiMoO_4_ at room temperature has been customarily achieved by using excess Ni in the precursor material or fixing the precursor material on a support [27]. Recently, β-NiMoO_4_ has been obtained using SCS [28]. β-NiMoO_4_ has been regarded, by many researchers, as the more catalytically active polymorph in catalytic reactions, such as the oxidation dehydration of petroleum derivatives [2]. By employing the SCS method, β-NiMoO_4_ can be stabilized under ambient conditions owing to the large amount of heat generated by the combustion reaction, leading to excess NiO in the lattice and helping in stabilizing β-NiMoO_4_ at room temperature [17].

An important aspect in establishing the presence of β-NiMoO_4_ is matching the observed XRD spectrum with readily available patterns in standard catalogs. A quick survey of the literature shows that researchers rely on (i) JCPDS 12-0348 or (ii) JCPDS 45-0142 to confirm the presence of β-NiMoO_4_. While in both cases, the most intense peak was seen at 26.6°, JCPDS 12-0348 reports another characteristic peak at 28.8°. In our view, the peak observed at 28.8° is not pertinent to β-NiMoO_4_ but rather to α-NiMoO_4_, and therefore JCPDS 45-0142 is an adequate indexed pattern to validate the presence of β-NiMoO_4_. The color of the sample is also indicative of the phase composition. Previous reports have indicated that β-NiMoO_4_ has a characteristic orange color compared to a yellowish-green shade for α-NiMoO_4_ [29].

**Figure 1:**
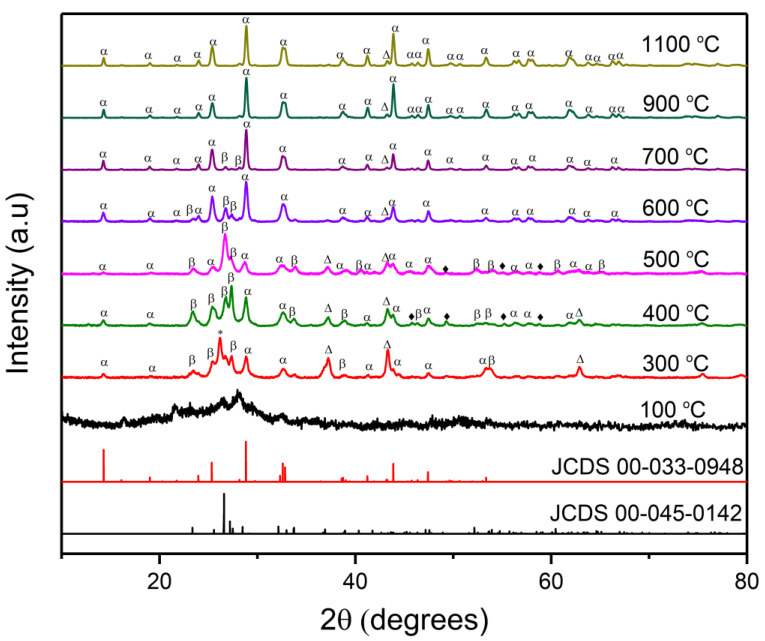
XRD patterns of NiMoO_4_ samples produced at different calcination temperatures; (α) α-NiMoO_4_; (β) β-NiMoO_4_, (*): MoO_2_, (♦) MoO_3_, and (Δ) NiO. Calcination time: 6 h, pH = 4.57, and fuel-to-oxidant ratio φ = 1.

Figure 1 shows the XRD patterns of the NiMoO_4_ samples prepared at different calcination temperatures. α-NiMoO_4_ (JCPDS 00-033-0948) and β-NiMoO_4_ (JCPDS 00-045-0142) reference diffraction patterns are also provided for comparative purposes. At 100 °C, the sample (which consisted of precursor materials at this stage) exhibits an XRD profile resembling a halo pattern, commonly associated with amorphous materials. Upon using a calcination temperature of 300 °C (post-combustion temperature), the sample shows sharp and defined peaks, evidence of high crystallinity. The phase composition at 300 °C appears to be a mix of α-NiMoO_4_, β-NiMoO_4__,_ and NiO (37.2° and 43.3°), in addition to displaying the presence of a high-intensity peak at 26.2° corresponding to MoO_2_, which constitutes much of the phase make up, as seen in Table 1. The color of the powder produced at this temperature was black, further signaling the disproportionate presence of MoO_2_ [30]. To ensure that the black shade is not carbon-related, EDX measurements were performed on the sample calcined at 300 °C and showed that it is carbon-free (Figure S1 in the supplementary document). The XRD spectrum of the sample prepared at 300 °C (Figure 1) shows that, despite the presence of a large number of undesirable phases, the crystallization and formation of β-NiMoO_4_ transpired at a temperature lower than that previously reported elsewhere [9].

At 400 °C, the black shade of the material dwindled, with the acquisition of a slight yellowish nuance, signaling the transformation of MoO_2_ to other phases. This was confirmed by semi-quantitative analysis (Table 1) and the reduced intensity of the MoO_2_ characteristic peak (26.2°), which was concomitant with the rise in the relative amounts of α-NiMoO_4_ and β-NiMoO_4_ (Figure 1 and Table 1). The sample calcined at 400 °C contained equal amounts of β-NiMoO_4_ and α-NiMoO_4_, albeit at larger quantities than the sample calcined at 300 °C. It is also evident that, at this calcination temperature, the intensity of the peak corresponding to NiO attenuated, while that of MoO_3_ increased. This is reasonable since MoO_2_ is stable in the temperature range of 225–350 °C, and in other studies, nanostructured MoO_3_ has been found to exhibit stability between 230 °C and 490 °C [31]. The comparative analysis of the XRD patterns suggest that between 300 °C and 400 °C, a portion of MoO_2_ changed to MoO_3_ and reacted with NiO to produce NiMoO_4_. At 500 °C, the major phase was, surprisingly, β-NiMoO_4_ with a small presence of α-NiMoO_4_ (Table 1). This means that there exists a temperature range within which β-NiMoO_4_ is preferentially produced, under the conditions employed in this work. The TGA/DSC analysis provided in our previous paper showed a minor, broad exothermic peak between 400–500 °C, without a discernable mass loss [22]. This peak is conjectured to be associated with further crystallization of β-NiMoO_4_ [32]. The sample produced at 500 °C acquired a dark-yellow color.

With a further increase in the calcination temperature to 600 °C (Figure 1), it is apparent that β-NiMoO_4_ underwent a major phase and crystallinity transformation, manifested by the diminution of the β-phase-related peak (26.6°) at the expense of an increase in that specific to the α-phase (28.8°), coupled with the appearance of green shade in the sample. At this temperature, the major phase was α-NiMoO_4_ (Table 1). Therefore, it can be said that the presence of β-NiMoO_4_ is initiated upon combustion and continues to rise with higher calcination temperatures, reaching a maximum at 500 °C. At 700 °C, the color of the sample became entirely green. A trace peak in the XRD pattern corresponding to β-NiMoO_4_ was still present, while the rest of the spectrum largely conformed to α-NiMoO_4_ (ca. 91 wt.%).

This result agrees with the findings of Moreno et al. [9], who reported that β-NiMoO_4_ has an upper-temperature limit of 600 °C. Figure 1 shows that the materials calcined at 900 °C and 1100 °C exhibited highly similar patterns associated with sharp peaks, indicative of an increased crystallinity and an overwhelming presence of α-NiMoO_4_ (Table 1). The color of the samples prepared at 700 °C, 900 °C, 1100 °C became increasingly greenish, implying the predominance of the α-NiMoO_4_ phase, in addition to a marked increase in density, as evidenced by the sharp, apparent drop in the powder volume, due to particle size growth by sintering. The XRD spectra at 900 and 1100 °C contained solely peaks specific to α-NiMoO_4_. The spectra show a major peak at 28.8°, which supports the fact that this peak is a fingerprint feature of α-NiMoO_4_.

In this study, it is remarkable that α-NiMoO_4_ remained stable even at temperatures higher than that at which the volatilization of MoO_3_ takes place. Rodriguez et al. [33] purported that the volatilization of MoO_3_ at 730 °C causes the decomposition of β-NiMoO_4_ and its transformation to NiO when cooled back to room temperature. On the other hand, Moreno et al. [9] affirmed that the β-NiMoO_4_ produced in their work did not transform into NiO even after cooling, stating that the obtained material was predominantly α-NiMoO_4_ at ambient conditions. Our results are in accordance with the findings of Moreno et al. [9] as calcination at high temperatures (700 °C, 900 °C, 1100 °C) resulted in the formation of α-NiMoO_4_, with no evidence of segregated phases.

**Figure 2:**
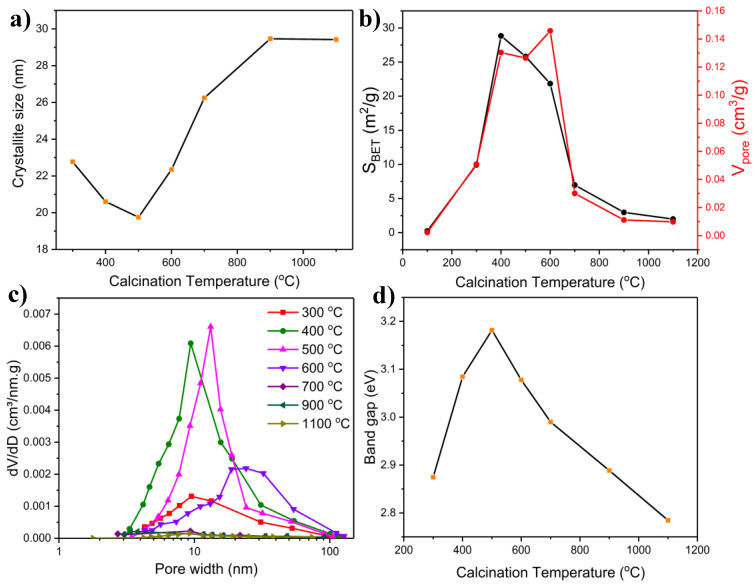
The effect of calcination temperature on the (**a**) crystallite size, (**b**) specific surface area and pore volume, (**c**) BJH pore size distribution, and (**d**) band gap of the synthesized NiMoO_4_ materials. Calcination time: 6 h, pH = 4.57, and φ = 1.

The calcination temperature also influences the crystallite size, as shown in Figure 2a. The crystallite size at 300 °C was larger than those at the two subsequent temperatures (400 and 500 °C). That is due to the presence of multiple phases at the lower temperature, including MoO_2_, which has the largest crystallite size compared to the other phases, as calculated by the Scherrer formula using its characteristic peak (26.1°)

The crystallite size decreased as the amount of MoO_2_ faded at higher calcination temperatures until reaching a minimum at 500 °C, where β-NiMoO_4_ was the predominant phase. Thereafter, it increased proportionally with respect to the calcination temperature, attaining a plateau at 900 °C. The increase in crystallite size is due to the Oswald ripening process associated with elevated calcination temperatures [19]. The change in crystallite size as a function of calcination temperature, in this work, agrees well with the observations of Selvan et al. [34], who employed SCS to produce MnMoO_4_ nanoparticles.

#### 2.1.2. FTIR Characterization

**Figure 3:**
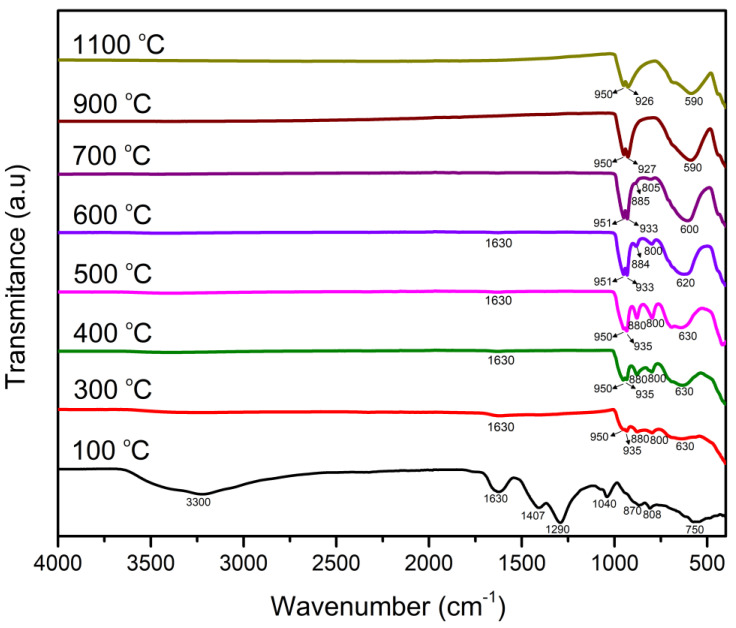
FTIR spectra of NiMoO_4_ samples produced at different calcination temperatures. Calcination time: 6 h, pH = 4.57, and φ = 1.

FTIR is a useful tool to distinguish between α-NiMoO_4_ and β-NiMoO_4_, each of which is associated with unique characteristic peaks. In Figure 3, the distinctive bands that are specific to β-NiMoO_4_ are those positioned at ~880 and 800 cm^−1^, which signify the tetrahedral position of Mo in the NiMoO_4_ lattice structure (structural characteristic of the β-phase) [35]. The broad peak at around 630 cm^−1^ indicates the presence of edge-shared MoO_6_ octahedra in α-NiMoO_4_ [6]. The peak at lower frequencies < 450 cm^−1^ designates the superposition of *v*_4_ and *v*_5_ vibration of MoO_6_ and the *v*_3_ mode of NiO_6_ building groups in NiMoO_4_ [6]. β-NiMoO_4_ and α-NiMoO_4_ also exhibited bands positioned in a close range. Particularly, the bands at 950–960 cm^−1^ and 930–940 cm^−1^ might be explained by either the activation of the ν_1_ vibration of the highly distorted MoO_4_ tetrahedra building block in β-NiMoO_4_ or the distorted MoO_6_ octahedral manifested in α-NiMoO_4_ [2]. Therefore, the bands at ~880 and 800 cm^−1^ are usually used to ascertain the presence or absence of β-NiMoO_4_ [36].

The FTIR results shown in Figure 3 are in good agreement with those obtained from the XRD spectra in Figure 1. At 100 °C (precombustion), the spectrum shows peaks related to the precursor’s undecomposed molecules. The band at 1407 cm^−1^ is consistent with the bending vibration of N–H of NH_4_^+^ groups that originated from ammonium heptamolybdate as a source of the Mo cation in the synthesis procedure. The band at 1290 cm^−1^ can be assigned to the symmetric vibrations of NO_3_^−^, originating from the utilization of Ni(NO_3_)_2_ as a Ni source. The band at 1040 cm^−1^ is associated with C-O bonds in agar. A drastic change occurred in the FTIR spectrum of the material as a result of the combustion reaction (the combustion reaction took place at ~147 °C as mentioned previously in the text). At 300 °C, it is notable that most of the precursor bands disappeared except for a minor peak at 1630 cm^−1^, which possibly emerges from the residual nitrate impurities stemming from the precursor [37]. Despite the clear transformation, the peaks pertaining to the β-NiMoO_4_ phase were not fully realized at 300 °C, in accordance with the XRD results (Figure 1). At 400 °C, the bands were more defined with a relatively higher intensity.

The peaks of the sample prepared at 500 °C were sharper and well-defined, especially those specific to β-NiMoO_4_ (880 and 800 cm^−1^). A drastic change in the 800–900 cm^−1^ region arises with a calcination temperature of 600 °C, similar to what was observed in the XRD plots in Figure 1. Indeed, at 600 °C, the intensity of the characteristic peaks of β-NiMoO_4_ decreased significantly, indicating a smaller presence of this phase in the oxide material (Table 1); additionally, at 600 °C, a red shift was observed at 600 cm^−1^ (at 500 °C, the peak position was at 620 cm^−1^). The slight shift in band positions was due to changes in the structure and mass of the sample as a result of the phase and density differences that transpired at different calcination temperatures. Higher calcination temperatures led to the disappearance of the β-NiMoO_4_-related peaks, which faded completely at 900 °C, evidencing the complete disappearance of the phase, in accordance with the results obtained by XRD (Figure 1 and Table 1). Since the FTIR spectra at 900 and 1000 °C contained exclusively α-NiMoO_4_, those spectra could serve as reference patterns for α-NiMoO_4_.

#### 2.1.3. BET Measurements

Figure S2 shows the N_2_ adsorption–desorption isotherms of the samples prepared at different calcination temperatures. These isotherms, which are of Type III, enabled the determination of the specific surface area, pore volume, and pore size of the materials. The isotherms are characterized by a little uptake of adsorbate (N_2_) in the low p/p_o_ region due to the relatively weak, adsorbent–adsorbate interactions and low microporosity. In the middle p/p_o_ region, a gradual increase in the adsorbate uptake was evident due to the formation of multiple layers of adsorbate on the adsorbent’s surface. At the high-relative-pressure end of the isotherm, a steep adsorbate uptake is noticeable due to the complete coverage of the surface, resulting in capillary condensation, whereby the adsorbate gets condensed in the pores at a pressure less than the saturation pressure of the bulk liquid. No saturation zone can be noticed in the region close to p/p_o_ = 1. In the desorption branch, a hysteresis effect is manifested, which is typical for mesoporous materials with a pore size greater than 4 nm. Hysteresis occurs as the adsorbate encounters metastability and/or network effects in the desorption branch, leading to capillary evaporation taking place at a lower pressure than capillary condensation [38]. The shape of the hysteresis loop bears a resemblance to type H3 hysteresis in the IUPAC classification [38], signifying that the stacking of individual particles generated the mesopores in the samples, and that the pores are irregular and open with good connectivity between intragranular pores with parallel, slit-like, and open-ended tubes [38].

**Figure 4:**
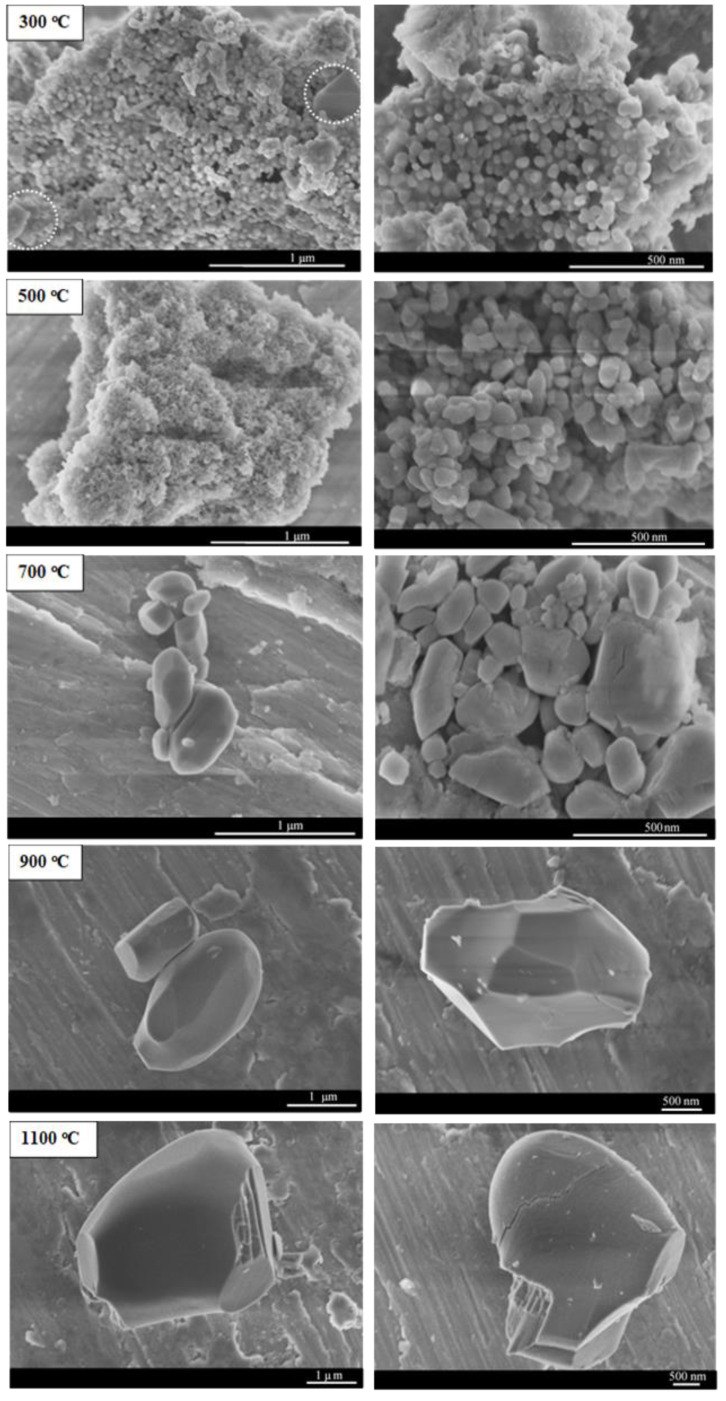
Representative SEM images illustrating the effect of calcination temperature on the surface morphology of NiMoO_4_ samples. Calcination time: 6 h, pH = 4.57, and φ = 1.

Figure 2b shows the evolution of the BET specific surface area and pore volume as a function of the calcination temperature. The surface area has been previously shown to be sensitive to the calcination temperature [39,40]. The specific surface area of the sample calcined at 100 °C was negligible since it constituted dried precursor materials, and it did not undergo a combustion reaction (the combustion reaction occurred at 147 °C [22]). At 300 °C, NiMoO_4_ was formed, and the specific surface area and pore volume increased significantly to 9.93 m^2^/g and 0.051 cm^3^/g, respectively. The specific surface area reached a maximum (28.8 m^2^/g) at 400 °C as more crystallization took place, concurrent with the increased presence of the α- and β-phase. The specific surface area and pore volume of the material calcined at 500 °C were also close to those recorded at 400 °C, with values of 25.8 m^2^/g, and 0.13 cm^3^/g, respectively. At 600 °C, the pore volume was still relatively large (0.14 m^2^/g), but the specific surface area encountered a drop to 21.9 m^2^/g. At 700 °C, the specific surface area and pore volume decreased precipitously due to sintering. In contrast, at higher temperatures, sintering became more pronounced, similar to what was previously observed in the crystallite size profile (Figure 2a). The particle size increased in an ascending fashion from 700 °C to 1100 °C (see Figure 4 later in the text), thereby reducing the specific surface area and the pore volume due to the collapse of the pore structure, destruction of the interagglomerate pores, and the closing of micropores [41].

The pore size distribution (PSD) of the synthesized materials at different calcination temperatures is shown in Figure 2c. At 300 °C, PSD covered a broad region centered at 9.5 nm. At 400 °C, the mean pore size was almost identical to that at 300 °C, but the PSD was narrower. A further increase in the calcination temperature resulted in an increase in the mean pore size to ~13.5 nm at 500 °C. PSD underwent a drastic change at 600 °C, showing a broad distribution (akin to a bimodal distribution) with an average at ~24 nm and a markedly broad PSD range. Elevated temperatures (700, 900, and 1100 °C) brought about an insignificant distribution with a noticeably broad and irregular profile. The total number of pores decreased as a result of sintering, while the amount of larger pore size increased. As noticed in other works, the higher the calcination temperature, the broader the PSD becomes, concomitant with large pore diameters [40]. This phenomenon can be attributed to sintering, destruction of micropores and mesopores, and rearrangement of the crystal network [42]. The increase in the pore size and decrease in the number of pores impacted the surface area and pore volume of the samples at higher temperatures (as seen in Figure 2b) due to a reduced void and interagglomerate space, which contributed to a lower surface area [42].

#### 2.1.4. Microscopy Characterization

SEM (Figure 4) and TEM (Figure S3) images were recorded to reveal the morphology and particle size of the materials prepared at different calcination temperatures. The sample prepared at 300 °C is associated with a high degree of agglomeration and shows, by and large, spherical particles of 120–150 nm in diameter. Morphological homogeneity was not observed at this temperature, as evidenced by the presence of differently shaped particles of random sizes (encircled in white in Figure 4), pointing to the existence of visibly segregated phases, which also agrees with the XRD results (Figure 1). At 500 °C, the material displayed a foamy morphology, which is a characteristic feature of materials produced through the combustion method, along with a quasi-spherical shape and size of 20–40 nm, with a moderate degree of agglomeration and high apparent porosity. The increase in foaminess and the smaller particle size contributes to the increase in specific surface area and pore volume, as seen in Figure 2b. Increasing the calcination temperature further led to a dramatic change in morphology and particle size. At 700 °C, the particles exhibited a polyhedral shape with attenuated porosity and a particle size range of 100–180 nm. The SEM images show that sintering was exacerbated beyond 600 °C, as is in line with the observations in Figure 2a,b. The morphological change with calcination temperature observed here is in line with the findings of Moreno et al. [9], who reported that, upon increasing the calcination temperature to 700 °C, a change in morphology of NiMoO_4_ to a polygonal shape could be observed with an increase in particle size and a less porous appearance. Due to sintering, the particle size increased to 1–2 µm at 900 °C and to 4–5 µm at 1100 °C, as also noted elsewhere [21]. SAED patterns portrayed in Figure S3 show the evolution of crystallinity with temperature, where between 300 and 700 °C, the patterns exhibited concentric circles and scattered dots, evidence of the samples’ polycrystallinity. At elevated temperatures (900 °C), due to the particle growth brought about by sintering, the SAED pattern signifies a single crystallite material.

#### 2.1.5. Band Gap Measurements

The optical properties of NiMoO_4_ samples synthesized at different calcination temperatures were assessed using UV/Vis spectrophotometry to determine the band gap values (the corresponding Tauc plots are presented in Figure S4). Both α-NiMoO_4_ and β-NiMoO_4_ are p-type semiconductors at room temperature [43]. NiMoO_4_ has two color centers stemming from the Ni and Mo complexes. It has been reported that the photon-induced exciton takes place through the *d*-*d* charge transfer from the O-2*p* to 3*d* orbital in Ni^2+^ or Mo^6+^ groups [44]. Electrical conductivity measurements indicate that the main surface defects are based on the model of doubly ionized vacancies for α-NiMoO_4_ and singly ionized vacancies for β-NiMoO_4_ [43]. A blue shift is commonly observed for nanometric, semiconducting materials, whereby the band gap increases as the size of the particles decreases due to quantum size effects [45]. Additionally, a red shift could take place due to chemical defects or vacancies in the intergranular regions [46].

Figure 2d shows the change in the band gap of the samples as a function of temperature. The relatively small band gap of the material prepared at 300 °C is due to the significant presence of MoO_2_. Conflicting reports about the band gap of MoO_2_ can be found in the literature, where some authors suggested that MoO_2_ exhibits a metallic behavior, while other findings purported that this material conveys a mixed semi-conductive/metallic behavior [31,47]. However, reduced MoO_3_ materials have been reported to attain a band gap between 2.4 and 2.7 eV, and recently Melo et al. reported a value of 1.83 eV for pure MoO_2_ [31]. Therefore, it is the large presence of MoO_2_ that explains the comparatively small band gap of the sample prepared at 300 °C. The band gap increased with increasing the calcination temperature as MoO_2_ subsided at the expense of the prevalence of α-NiMoO_4_, β-NiMoO_4_, and MoO_3_ (Table 1), all of which exhibited larger band gaps. Beyond 500 °C, the particle size increased, as shown in Figure 4 and Figure S3, due to sintering, leading to a reduction in the band gap due to the enlarged particles’ effect [48]. Previous studies on the conductivity of NiMoO_4_ have stated that for both phases (α- and β-NiMoO_4_), electrical transport occurs through the classic intrinsic band conduction mechanism, with the band gap of β-NiMoO_4_ being larger than that of α-NiMoO_4_ according to DFT results [49]. This agrees with the results presented in Figure 2d where the sample with the largest band gap was the one with the most prevalent amount of β-NiMoO_4_ (500 °C). However, other factors can also affect the band gap, including but not limited to particle size, morphology crystallite size, and agglomeration [50].

### 2.2. Effect of Calcination Time

After discerning the influence of the calcination temperature on the microstructure and the selected physicochemical properties of the synthesized materials, it was important to evaluate the impact of the calcination time on the samples’ physicochemical and structural characteristics.

The effect of calcination time is a crucial aspect to consider when preparing nanometric products using the SCS method. While some materials require a short calcination time to acquire the desired phase, others must undergo prolonged calcination periods to attain the required physicochemical/structural properties. The calcination time has been reported to be consequential to particle growth and phase evolution in addition to other physicochemical changes, as observed in the literature [19]. In the following section, the effect of calcination time was investigated at different time increments and at a fixed calcination temperature of 500 °C.

#### 2.2.1. XRD Measurements

The effect of the calcination time on the crystallinity and phase composition of the produced materials was studied using XRD, as shown in Figure S5a. The XRD spectra suggest that the crystallinity undergoes a minor change as the calcination time increases, evident from the invariability in the peaks’ sharpness. However, the phase composition encounters marked transformation with short calcination durations. The as-combusted sample (removed immediately from the furnace after the onset of the combustion reaction) exhibited a marked level of MoO_2_ with equivalent amounts of β-NiMoO_4_ and α-NiMoO_4_ in addition to a small presence of NiO and MoO_3_, as shown in Table 2. Previous studies indicated that the decomposition of agar leaves some residual ash (20%) even after exposing it to a temperature of 600 °C [51]; however, in our case, no agar residual was observed/detected due to the high temperature associated with the SCS synthesis procedure, arising from the combustion reaction [12]. The color of the as-combusted sample, at this stage, was still dark grey (similar to that at 300 °C, discussed in the previous section), reflecting the presence of a considerable amount of MoO_2_ in the sample (Table 2). No residual carbon from the precursor was present in this case, as evidenced by the EDX results (Figure S6). It is worth noting that the XRD spectrum of the as-combusted sample (Figure S5a) looks highly similar to the sample calcinated at 300 °C (Figure 1), emphasizing the importance of both the calcination time and calcination duration.

As the calcination time was increased to 5 min, the characteristic peak of β-NiMoO_4_ grew slightly relative to that of α-NiMoO_4_ and the intensity of MoO_3_ characteristic peaks increased, while the peaks relevant to NiO declined relative to the as-combusted sample (Figure S5a). With 20 min of calcination, the peaks in the XRD pattern appeared more developed and exhibited an increased level of β-NiMoO_4_. Calcinating the material for 60 min led to a more pronounced appearance in the characteristic peak of β-NiMoO_4_. The maximum amount of β-NiMoO_4_ was achieved in the sample calcined for 360 min (Table 2). As the calcination time was extended further, a small change in the XRD spectra was observed (Figure S5a), where at prolonged calcination times (720 min, 1440 min) the amount of β-NiMoO_4_ dropped slightly, while that of α-NiMoO_4_ increased. The change in the phase composition at extended calcination time is unsurprising based on findings elsewhere. Alibe et al. [19] assessed the effect of the calcination holding time on several physicochemical properties of Willemite nanoparticles, observing an increase in particle size and an appearance of single-phase Willemite at long calcination times. The same conclusions were reached by Ravanchi and coworkers [42], who reported that even a slight difference in calcination time could bring about a phase change in their studied materials (alumina). In another study, Zhang et al. [52] assessed the influence of calcination time on the physicochemical characteristics of porous TiO_2_-Al_2_O_3_ nanomaterial. They noticed that despite the slight change observed in the phase composition, the crystallite size, specific surface area, pore volume, and average pore width changed with calcination time. These parameters are investigated in the following sections.

**Figure 5:**
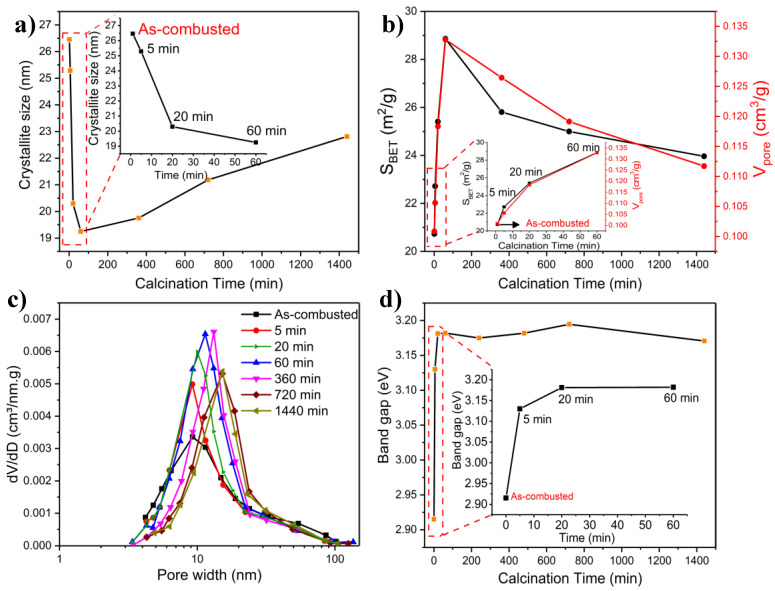
The effect of calcination time on the (**a**) crystallite size, (**b**) specific surface area and pore volume, (**c**) BJH pore size distribution, and (**d**) band gap of the synthesized NiMoO_4_ materials. Calcination temperature: 500 °C, pH = 4.57, and φ = 1.

Figure 5a shows the effect of calcination time on the crystallite size. It is evident that the smallest crystallite size was achieved with a calcination duration of 60 min, beyond which increasing the calcination duration amplified the crystallite size in almost a linear trend. The crystallite size of the as-combusted sample and that calcinated for 5 min were almost identical (25.7 and 24.6 nm, respectively). The comparatively large crystallite size of the as-combusted sample can be, to a certain extent, linked to the presence of MoO_2_, akin to the premise provided in Section 2.1. in relation to the sample calcined at 300 °C. With an increase in the calcination time, the crystallite size decreased and reached a minimum as the calcination time increased to 60 min. Interestingly, as is shown in Figure 5b, the sample calcined for 60 min achieved the largest specific area. This effect of calcination duration on crystallite size has also been corroborated in previous studies, namely that of Porter et al. [53], who purported that crystalline phases exhibit varying crystal sizes and growth profiles. Figure 5a shows that extending the calcination time past 60 min resulted in the growth of the crystallite size, reaching around 23 nm after 1440 min (24 h) of calcination. The increase in crystal size observed after 60 min can be ascribed to the removal of grain boundary defects, sintering, and growth of crystals through coalescence [19,54].

#### 2.2.2. FTIR Characterization

The FTIR spectra in Figure S5b confirm the results obtained with XRD. The FTIR pattern of the as-combusted sample experienced a major transformation compared to the precursor, as evidenced by the drastic change in the FTIR profile. The FTIR spectrum of the as-combusted sample shows that the characteristic bands of β-NiMoO_4_ at 800 and 880 cm^−1^ were present, although at a low relative intensity. Increasing the calcination time to 20 min showed that the peaks specific to β-NiMoO_4_ were almost fully developed. Beyond 20 min, the FTIR spectra do not display any major transformation. However, in accordance with the XRD results presented in Figure S5a, β-NiMoO_4_-related characteristic peaks dwindled at extended periods (1440 min). The impurity peak at 1630 cm^−1^—corresponding to nitrate–can be observed at a minimal intensity in all the samples.

#### 2.2.3. BET Measurements

The N_2_ adsorption–desorption isotherms are presented in Figure S7. Similar to the isotherms seen in Figure S2, the isotherms of the material prepared at different calcination times correspond to Type III isotherm with H3-type hysteresis, indicating mesoporous structures. The as-combusted sample showed a mesoporous characteristic with a specific surface area and pore volume of 20.7 m^2^/g and 0.10 cm^3^/g, respectively (Figure 5b). Longer calcination (5 min) entailed a minor change in the isotherm profile and pore volume but with an uptick in specific surface area (22.7 m^2^/g), which was probably due to the changes in the phase composition (Table 2) and the elimination of residual elements from the as-combusted material, which originated from the precursor material. As the calcination time was raised, the specific surface area and pore volume increased, achieving a maximum of 28.8 m^2^/g and 0.13 cm^3^/g with 60 min of calcination, respectively. Afterward, both the specific surface area and pore volume decreased with longer calcination times due to sintering and crystallite growth (Figure 5a). It is not completely clear what the origin of the maxima at 60 min could be. However, it can be conjectured that this was a result of two opposing forces: the change in phase composition (increase in the β-phase) and particle growth by sintering [2,53].

The PSD of the samples synthesized over varying calcination periods is presented in Figure 5c. The materials exhibited similar pore size distribution profiles. The as-combusted sample had a small mean pore size of 9.2 nm with a wide distribution. Upon further calcination, the PSD of the sample calcined for 5 min became narrower with a mean peak size identical to that of the as-combusted material. After 20 min of calcination, the PSD shifted slightly to a larger mean pore size with a similar distribution profile compared to the samples prepared at shorter calcination times. The sample calcined for 60 min showed an additional limited shift in the mean pore size with a large number of pores concentrated in a narrow range. After 360 min of calcination, PSD underwent a slight shift with a mean pore size of 13.2 nm, in which most of the pores ranged between 4.3 and 24.2 nm. At prolonged calcination times, PSD became broader, while the mean peak size shifted to 15.2 nm for the samples calcined for 720 and 1440 min, with most of the pores occurring in the 4.8–31.4 nm range. The increase in the mean pore size at extended calcination periods was possibly due to the intra-agglomerate densification or intercrystallite sintering in the agglomerates [53]. While Figure 5c suggests some variation in the PSD profile and mean pore size of the samples prepared at different calcination durations, PSD was hardly influenced by the calcination times beyond 60 min.

#### 2.2.4. Microscopy Characterization

SEM and TEM images (Figures S8 and S9) substantiate the previously mentioned observations showing the minor influence of calcination duration on the prepared samples (Figure 5). The as-combusted sample was highly agglomerated with particles of irregular shape, ranging between 20 and 45 nm (Figure S8). Similar to the sample prepared at 300 °C, the as-combusted sample exhibited segregated phases (encircled in white in Figure S8). At this stage, the sample still contained some remnants, at minor levels, from the precursor materials; however, the microstructure of this sample (Figures S8 and S9) exhibited a highly foamy and spongy morphology owing to the combustion reaction. A slight increase in the calcination time (5 min) resulted in a reduced degree of aggregation and the disappearance of the visibly segregated phases while maintaining the signature foamy appearance specific to the SCS method (Figure S8). Prolonged calcination durations (20 min, 60 min, 1440 min) did not entail a consequential modification in terms of morphology (Figure S8) or phase segregation (Figure 5a). The SAED patterns shown in Figure S9 indicate that the as-combusted material was polycrystalline. With an extended calcination time of 1440 min (24 h), the SAED pattern slightly changed, compared to the as-combusted sample.

#### 2.2.5. Band Gap Measurements

The band gaps of the materials prepared at different calcination times were assessed using the Tauc plot method (Figure S10), and the corresponding values are shown in Figure 5d. The band gap of the as-combusted material was 2.91 eV, which increased markedly to ~3.13 eV as the calcination time increased to 5 min. The increase was due to the elimination of MoO_2_, which had a lower band gap than the other oxide component materials, as mentioned previously (Section 2.1). Additionally, it is worth noticing that the band gap of the as-combusted sample was in close range with the sample calcinated at 300 °C (Figure 2d), both of which have similar physicochemical properties. At 20 min and beyond, the band gap of the samples became almost invariable (Figure 5d), an observation that is supported by the notion that the calcination time had a little impact on the physicochemical properties of the samples at extended calcination periods [55].

### 2.3. Effect of Composition

The effect of composition (atomic ratio of the constituent elements, Ni and Mo) is of interest due to its marked influence, among other things, on a material’s catalytic affinity. NiMo-oxide materials, both at the macro- and nanoscale, have been used in a host of catalytic applications with a wide range of compositions. Previous studies [2] on the catalytic activity of NiMoO_4_ of varying Ni/Mo ratios have shown that the following phases could be present: nickel oxide, molybdenum trioxide, nickel molybdate (α- and β-NiMoO_4_), and non-stoichiometric nickel molybdates in which a solid solution of nickel is dissolved in the molybdate lattice. Despite the common belief that secondary phases are undesirable and that the β-NiMoO_4_ polymorph is more catalytically active than its α- counterpart, the effectiveness of the phase is, in fact, application-related. For example, Ozkan and Scharader [56] established that the presence of excess MoO_3_ is paramount in increasing the catalytic activity of nickel molybdate in selective oxidative reactions. Additionally, Barrault et al. [57] proved a synergetic effect that exists between MoO_3_ and α-NiMoO_4_, whereby the maximum activity of propane oxidation takes place at a ratio of 0.25. On the other hand, Pilipenko et al. [58] investigated the effect of the composition of the NiMo-oxide system on butadiene production. They reported that the activity decreases with an increase in the level of MoO_3_. Additionally, Mo-rich catalysts were found to be five times more efficient than Ni-rich products for the oxy-dehydrogenation of propane into propene based on the findings of Thomas et al. [59]. Itenberg and coworkers [60] studied the effect of the Ni/Mo atomic ratio on the n-butene-to-butane conversion reaction and noted that excess Ni yields the maximum selectivity in the process. Therefore, it is important to study the influence of NiMo-oxide samples prepared with different atomic ratios on their physicochemical/structural properties. A calcination time and calcination period of 500 °C and 6 h, respectively, were adopted to produce samples of different Ni/Mo atomic ratios, and the corresponding results are presented as follows.

#### 2.3.1. Combustion Parameters

**Figure 6:**
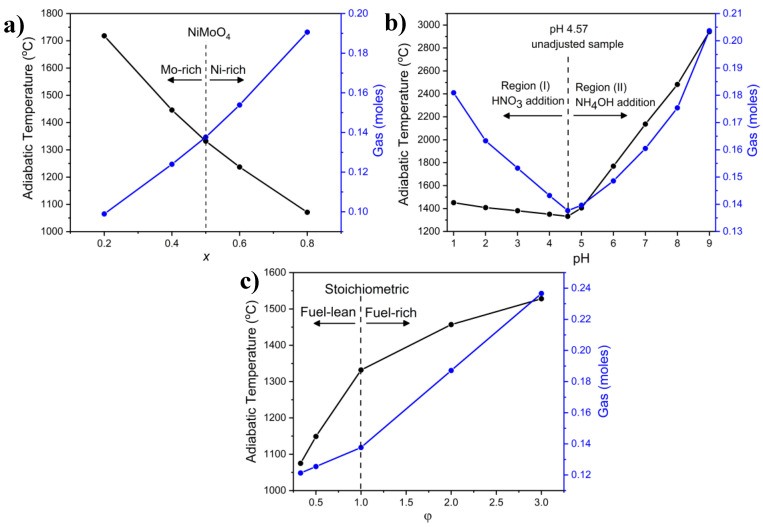
Variation in the theoretically calculated adiabatic temperature and amount of gas evolved as a function of (**a**) *x*, where *x* stands for the atomic fraction of Ni in Ni*_x_*Mo_1-*x*_-oxide, (**b**) pH of the precursor solution, and (**c**) fuel-to-oxidant ratio (φ).

As the production of the nanostructured NiMoO_4_ samples in this work was based on the SCS approach, it is important to shed light on some thermodynamic considerations that exhibit a direct impact on the observed physicochemical properties. It has been established that two main factors are impactful on the surface characteristics of materials synthesized by the SCS method: (i) the flame (adiabatic) temperature, T_ad_, which represents the maximum theoretical temperature that occurs inside the combusted mixture without accounting for thermal losses and (ii) the amount of generated gases that is emitted from the combusted medium [61]. Thermodynamic calculations (using thermodynamic data in Table S1) allowed the determination of T_ad_ based on Equations (S1)–(S3) shown in the supplementary document, while simple stoichiometric ratios based on the overall reaction of NiMoO_4_ production shown in Equation (S4) enabled the computation of the evolved gas amounts. Figure 6a shows the variation in T_ad_ and the amount of generated gas as a function of the material composition (Ni*_x_*Mo_1-*x*_-oxide, where *x* represents the atomic percentage of Ni in the compound).

It is evident that T_ad_ decreased as the Ni content increased in the oxide, while the generated gas followed the opposite trend. The decrease in T_ad_ with an increasing *x* is attributed to the relatively lower total enthalpy content at higher *x* due to the use of a larger amount of Ni(NO_3_)_2_. The increase in the evolved gas is ascribed to the fact that Ni(NO_3_)_2_ contributes more to the amount of gaseous products than (NH_4_)_6_Mo_7_O_24_, as calculated using basic stoichiometric ratios. These results carry major implications on the physicochemical properties and microstructural aspects of the samples prepared with different Ni/Mo ratios, as it will be shown in the following sections.

#### 2.3.2. XRD Measurements

Figure S11a shows the XRD patterns of Ni*_x_*Mo_1-*x*_-oxide (*x* = 0.0, 0.2, 0.4, 0.5, 0.6, 0.8, 1). NiO (*x* = 1), and MoO_3_ (*x* = 0) could be readily referenced with the indexed patterns (JCPDS, no. 01-071-1179) and (JCPDS, no. 00-035-0609), respectively. The actual compositions of the synthesized samples were validated using ICP-OES (Table 3), which confirmed that both the nominal and experimental compositions are practically identical. Figure S11a and Table 4 show that the spectrum at *x* = 0.2 closely resembles that of MoO_3_ (*x* = 0), indicating that the phase composition consists largely of MoO_3_ with insignificant amounts of α-NiMoO_4_ or β-NiMoO_4_.

At *x* = 0.4, a phase shift is notable since the peaks specific to β-NiMoO_4_ and α-NiMoO_4_ became more prevalent, while those relevant to MoO_3_ persisted. This observation has also been reported in the literature [62]. In the equiatomic stoichiometric sample (*x* = 0.5), there was a drastic change in the XRD pattern as the β-NiMoO_4_ peaks were more pronounced. The peaks corresponding to MoO_3_ were considerably attenuated, while those of NiO underwent a slight increase in intensity. At *x* = 0.6, the XRD pattern bore a high resemblance to that of *x* = 0.5, with a highly similar phase composition (Table 4). The relative preponderance of β-NiMoO_4_ at *x* = 0.5 and 0.6, compared to those below *x* = 0.5, confirms the role that excess NiO plays in the stabilization of β-NiMoO_4_ at room temperature. It has been reported that Ni-enriched samples contribute to the prevalence of β-NiMoO_4_ since Ni cation ions can replace Mo cations in the NiMoO_4_ lattice structure, thereby stabilizing β-NiMoO_4_ at ambient conditions [17].

At *x* = 0.8, the phase composition distribution was not computed since, at this sample, a relatively substantial broadness was observed in the peaks associated with NiO (37.3° and 43.3°) and would therefore render the phase analysis invalid based on the method used in this study. However, since this sample was prepared with excess Ni with a significant shortage of Mo in the system, the formation of NiMoO_4_ faded, while the presence of NiO increased significantly compared to *x* = 0.6 and *x* = 0.5. This is corroborated in the FTIR spectra (Figure S11b), which show that peaks related to NiMoO_4_ were significantly diminished.

**Figure 7:**
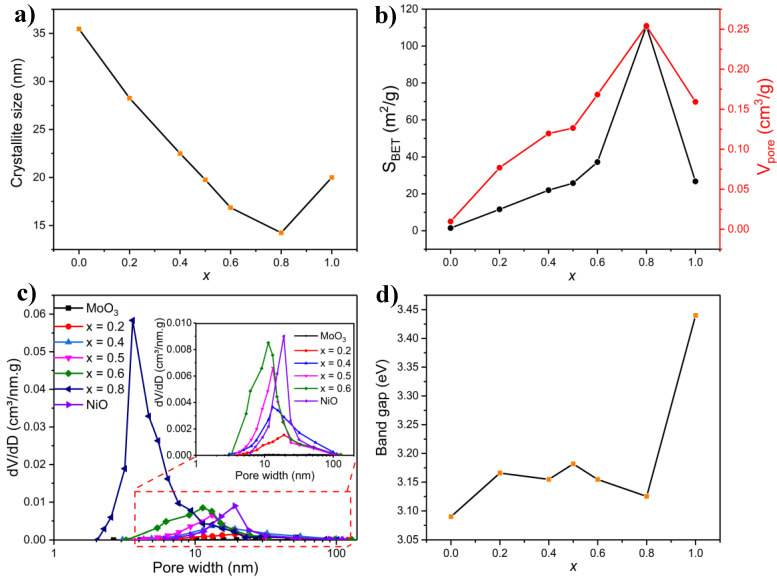
The effect of composition (depicted by *x*, which stands for the atomic fraction of Ni in Ni*_x_*Mo_1-*x*_-oxide) on the (**a**) crystallite size, (**b**) specific surface area and pore volume, (**c**) BJH pore size distribution, and (**d**) band gap measurements of the synthesized of Ni*_x_*Mo_1-*x*_-oxide samples. Calcination temperature: 500 °C, calcination time: 6 h, pH = 4.57, and φ = 1.

The influence on crystallite size brought about by the change in composition is due mainly to the change in T_ad_, and the amount of evolved gases in addition to the change in phase composition, as seen in Figure 7a. While higher T_ad_ leads to sintering and particle growth, gas evolution counteracts this effect due to the cooling effect it elicits in the combusted medium [63]. During the combustion process, the amount of the released gases (such as CO, CO_2_, NO, NO_2_, NH_3_, water vapor, etc.) is a controlling factor that affects properties such as crystallite size, specific surface area, and porosity (Figure 7) by preventing the formation of a dense structure and by disintegrating the large particles to yield nanometric structures. The crystallite size decreased as *x* increased, owing to the decrease in T_ad_ and increase in the volume of generated gases, both of which are favorable for the formation of smaller crystallites (Figure 7a). The increase in the crystallite size at *x* = 1 (NiO) can be explained by the lack of (NH_4_)_6_Mo_7_O_24_ in the synthesis procedure and consequently the absence of ammonia which, as it will be seen later in the paper (Section 2.4), is vital in intensifying the combustion reaction.

#### 2.3.3. FTIR Characterization

FTIR was used to confirm the phases present in the samples. The FTIR spectra of pure oxides (NiO and MoO_3_) are similar to those reported in the literature. At *x* = 0.2, the FTIR spectrum looks highly similar to that of pure MoO_3_, evidenced by the bands at 814 and 857 cm^−1^, which stem from MoO_3_, whereas there is a small band at around 930 cm^−1^, which corresponds to α-NiMoO_4_. At *x* = 0.4, a significant transformation in the FTIR spectrum occurred, as manifested by the appearance of the characteristic peaks of β-NiMoO_4_ at 880 and 800 cm^−1^, in addition to those at 950 and 600 cm^−1^ specific to α-NiMoO_4_. At *x* = 0.5, the β-NiMoO_4_ peaks seem to be fully realized, accompanied by a new broad peak at 650 cm^−1^. At *x* = 0.6, the spectrum is similar to that of *x* = 0.5, except for the absence of the broad peak at 650 cm^−1^, indicating a change in the material’s structure. At *x* = 0.8, the FTIR spectrum is highly akin to that of NiO with notable peaks related to the presence of β-NiMoO_4_ but with a much lower intensity than those at *x* = 0.6 and *x* = 0.5. The information yielded from the FTIR spectra (Figure S11b) agrees well with the observations obtained from the XRD (Figure S11a) and are similarly reported elsewhere [2]. Notably, the FTIR spectra corroborated the findings related to the proliferation of the α-phase in the samples that contain excess Mo (region *x* ≤ 0.4), while the β-phase was prevalent in the samples with excess Ni, confirming the notion that the presence of excess Ni in the precursor solution helps to stabilize the β-phase at ambient conditions [2].

#### 2.3.4. BET Measurements

The specific surface area and pore volume of Ni*_x_*Mo_1-*x*_-oxide (0 ≤ *x* ≤ 1) samples, derived from the N_2_ adsorption–desorption isotherms shown in Figure S12, are displayed in Figure 7b. All the isotherms are consistent with Type III, according to IUPAC classification with H3 hysteresis (similar to the samples discussed in the previous sections). The specific surface area and pore volume changed significantly with the composition, as depicted in Figure 7b. For MoO_3_ (*x* = 0), the specific surface area and pore volume were almost negligible. As *x* increased to 0.2, the specific surface area increased 10-fold, reaching 11.6 m^2^/g, along with an increase in pore volume. The specific surface area and pore volume continued to rise as the atomic fraction of Ni in NiMoO_4_ increases, reaching a maximum at *x* = 0.8 (110 m^2^/g, 0.25 cm^3^/g). Interestingly, the specific surface area and pore volume at *x* = 0.8 were starkly greater than those of any other composition. This is despite the increase in the NiO phase, which, according to previous reports [2], should have reduced the surface area. The specific surface area and pore volume of NiO were considerably lower (26.7 m^2^/g and 0.16 cm^3^/g, respectively).

These observations can be explained by the basic principles of the SCS method. Increasing the Ni content in NiMoO_4_ required increasing the amount of the Ni salt precursor (Ni(NO_3_)_2_), which has an oxidizing power, while the amount of agar remained constant. This gave rise to different fuel systems (fuel-rich for *x* < 0.5 and fuel-lean for *x* > 0.5). As previously mentioned, T_ad_ and the amount of evolved gases are two main (often competing) forces that dictate the surface area and the level of agglomeration, morphology, and particle size of the material [63]. While high T_ad_ elicits sintering, lessening of the surface area, and enlarging of the crystallite size, the amount of gas produced in the combustion reaction can counter those effects as gas generation helps dissipate the heat of combustion and offsets the rise in temperature, which creates pores, limits particle growth, and increases the specific surface area. The combination of low T_ad_ and high gas liberation contributes to a high specific surface area, with the large pore volume and smallest pore size and crystallite size (compare the trends in Figure 6a and Figure 7a,b). When the combustion reaction gas is released, it creates a cooling effect, allowing the removal of a large amount of heat, resulting in smaller grains. The amount of generated gas contributes to the formation of porous, foamy products with a high surface area if T_ad_ is not elevated enough to reverse those effects. For these reasons, the surface area and pore volume increased at an accelerating rate with a rising *x* since this was concomitant with a decline in T_ad_ and an increase in gas volume. The appreciable decline in the surface area of NiO (*x* = 1), compared to *x* = 0.8, points to the importance of (NH_4_)_6_Mo_7_O_24_ in the precursor solution. This is because the synthesis procedure of NiO lacks (NH_4_)_6_Mo_7_O_24_, and consequently ammonia. Ammonia, as it will be seen later in the paper (Section 2.4), plays an important role in intensifying the combustion reaction.

The PSDs of the Ni*_x_*Mo_1-*x*_-oxide (0 ≤ *x* ≤ 1) samples are depicted in Figure 7c. Notably, PSD changed markedly as the amount of Ni in the samples increased. For MoO_3_, the PSD was almost unnoticeable due to the low porosity of this material. With increasing *x*, the pore size distribution became narrower, and the average pore size decreased. Notably, at *x* = 0.8, the mean pore size underwent a significant decline to 3.5 nm. As explained previously, the relatively mild T_ad_ and the large amount of exhausted gases, seen at higher *x* (Figure 6a), are responsible for shrinking the PSD, lowering the average pore size, and increasing the porosity. The PSD of NiO shows a narrow distribution with a relatively large mean pore size.

#### 2.3.5. Microscopy Characterization

SEM and TEM images (Figures S13 and S14) were recorded for Ni*_x_*Mo_1-*x*_-oxide (0 ≤ *x* ≤ 1) samples. For MoO_3_, the particle exhibited a hexagonal shape with a particle size range between 200 and 350 nm, and low apparent porosity, consistent with Figure 7b. Additionally, the SAED pattern in Figure S14 indicates that the material is of a single-crystal nature, as evidenced by the presence of discrete spots. Increasing the amount of Ni in the material gives it a slightly more porous form. For instance, at *x* = 0.2 (Figure S13), the sample consisted of a mix of large particles of a hexagonal shape corresponding to MoO_3_ and another type of round and porous particles, attributed to NiMoO_4_, also evidenced by studies elsewhere [56], which is in line with the results of the XRD (Figure S11a). The low porosity and the relatively large particle size stem from the high T_ad_ and a comparably small amount of evolved gas, as is illustrated in Figure 6a and Figure 7c. Due to the presence of segregated phases (MoO_3_ and NiMoO_4_), two particle sizes were present: those between 20 and 40 nm ascribed to NiMoO_4_ and the larger particles (200–350 nm) corresponding to MoO_3_ (Figure S13). Additionally, the SAED patterns (Figure S14) show a drastic transformation in crystallinity, as apparent from the appearance of well-defined rings composed of bright spots evidencing the polycrystalline structure of the material.

An additional increase in Ni (*x* = 0.4) in Ni*_x_*Mo_1-*x*_-oxide caused the formation of smaller particles with a drastic change in morphology, which is associated, to a large extent, with quasi-spherical particles in addition to a haphazard presence of the MoO_3_-related hexagonal shapes. The sample also showed a drastic increase in porosity, in line with Figure 7c, due to a more vigorous gas generation rate and lower T_ad_ compared to *x* = 0.2. The SAED pattern was practically identical to the sample of *x* = 0.2. With the equiatomic stoichiometric sample, *x* = 0.5 (shown in Figure 4 as the sample prepared at 500 °C), the material exhibited, in large proportions, agglomerates of porous particles with a sponge-like, foamy appearance morphology—a hallmark of materials synthesized by the combustion route—with a particle size of 20–40 nm and an SAED pattern (Figure S14) identical to the samples of lower *x*. In the presence of excess Ni (*x* = 0.6), the morphology became more porous and foamier, characterized by smaller particles between 20 and 30 nm. At *x* = 0.8 when the material attained its maximum specific area and porosity, as seen in Figure 7b, the sample appeared to have a highly porous morphology (also corroborated in Figure 7c) with remarkably tiny particles in the range of 3–6 nm (Figures S13 and S14). This is due, in large part, to the low T_ad_ and a large amount of evolved gas (Figure 6a). A low-magnification SEM image showing the extent of porosity at *x* = 0.8 is also provided in Figure S15. Additionally, it is apparent that the sample was no longer polycrystalline, indicating a degree of amorphousness, evidenced by the halo rings in its SAED pattern, as shown in Figure S14. NiO (*x* = 1) exhibited spherical particles between 20 and 30 nm with a marked absence of “fluffiness”. The SAED pattern (Figure S14) shows rings made of equivalently sized spots, indicating equally sized crystals and a polycrystalline nature.

#### 2.3.6. Band Gap Measurements

The impact of sample composition on the band gap of Ni*_x_*Mo_1-*x*_-oxide was evaluated using UV-Vis spectra from which the Tauc plots were obtained (Figure S16). As shown in Figure 7d, the band gap of the materials with varying *x* changed scantly except for NiO (*x* = 1), which exhibited a band gap of 3.44, in line with other findings [64]. The small dependence of the band gap on the sample composition (0.2 ≤ *x* ≤ 0.8) is an intriguing observation since, with an increase in Ni content in the sample, the corresponding band gap was expected to increase considering that MoO_3_ and NiO have different band gap values (3.13 eV for MoO_3_ vs. 3.44 eV for NiO) [22]; hence, the increase in the amount of NiO should have reasonably brought about a larger band gap. However, for materials on the nanoscale, quantum effects are prevalent. It is well-known that for nanomaterials, a reduction in particle size leads to an increase in the band gap (blue shift). A blue shift takes place when the conduction band edge (CB) shifts to higher energy, coincident with a drop in the valence band edge (VB) to lower energy, resulting in a larger band gap in the band structure [48]. This effect is likely to become predominant with increasing the amount of Ni in Ni*_x_*Mo_1-*x*_-oxide, in accordance with the results in Figures S13 and S14, which confirmed a decrease in the particle size at higher Ni/Mo ratios. However, since Figure 7d shows that in the range of 0.2 ≤ *x* ≤ 0.8 the band gap did not follow any trend, it is suggested that phase, compositional, and structural changes significantly govern the band gap values. Another important aspect to consider is the change in the type of semiconductivity with varying Ni/Mo ratios: for Ni-rich materials, a p-type semiconductivity was observed, while an n-type was prevalent for Mo-rich compounds [59].

For comparative purposes, a commercial NiMoO_4_ was purchased and subjected to different characterization techniques, as shown in Figure S17. The commercial sample featured a scattered, broken nanorod morphology with a BET surface area and pore volume of 19.8 m^2^/g and 0.085 cm^3^/g, respectively. The samples belong to the hydrated NiMoO_4_ phase with an absence of the α and β polymorphs, unlike the samples produced in this study. The pore size distribution exhibited a mean pore size of ~35 nm, which is much higher than those of the samples produced here. The band gap of the commercial sample was also larger (3.31 eV) compared to the samples produced in this work.

### 2.4. Effect of the pH of the Precursor Solution

The effect of the precursor solution’s pH in the SCS method has been extensively studied in the literature [12]. The pH has a direct influence on the intensity of the combustion reaction, in addition to playing a key role in gauging the dissociation degree of the organic fuel and their affinity to chelate metal cations. The pH can impact a wide range of physicochemical properties, namely microstructure and morphology, crystallite and particle size, surface area, pore volume, and pore size [65]. Because a lack of control over the pH could lead to the precipitation of the metal ions through the formation of hydroxides or salts, the proper tuning of pH is of the essence. Therefore, it was important to investigate the effect of pH on the structural/morphological properties of the NiMoO_4_ samples produced in this work. The NiMoO_4_ samples discussed in the following text were prepared at a calcination temperature of 500 °C for a period of 6 h.

#### 2.4.1. Combustion Parameters

One of the factors that makes the precursor solution’s pH impactful in influencing the physicochemical properties of the combusted products is the use of additives such as HNO_3_ and NH_4_OH, which are considered oxidants, to adjust the pH of the starting solution. Both additives were employed in our study since they enhance the dissolution of metal precursor salts and are also widely utilized in SCS studies [61]. In this work, the pH of the precursor solution without adjustment was 4.57, and this is conveniently referred to in the text as the control sample. For pH modifications, HNO_3_ was utilized to lower the precursor solution’s pH and NH_4_OH was used to raise it. Based on the thermodynamic stoichiometric calculations portrayed in Figure 6b, in the region where HNO_3_ was added (Region I), at pH = 1, the amount of generated gas was significantly higher than that generated with the control sample (pH 4.57), whereas the difference in T_ad_ was more subdued. As the pH increased, the volume of the gas generated and T_ad_ decreased, reaching an inflection point at the control sample. In Region II where NH_4_OH was added, T_ad_ rose at a markedly higher rate than in Region (I), while the increase in the evolved gas was on par with that in Region (I). As the pH was further increased, T_ad_ rose significantly, reaching ca. 3200 °C at pH = 9 with ca. 0.21 mol of evolved gas. The results show that the addition of NH_4_OH had a more pronounced impact on the combustion process than HNO_3_. These findings underscore, among other things, the role that pH adjustments of the precursor solution could play in impacting the physicochemical properties of the samples, as will be shown further in the text. A possible explanation for the pronounced effect of NH_4_OH (Region II) on the combustion dynamics is ascribed to the production of NH_4_NO_3,_ which is considered a super oxidant, as an intermediate in the reaction pathway [66]:

Ni(NO_3_)_2_ + 2NH_4_OH → Ni(OH)_2_ + 2NH_4_NO_3_ (1)

Peng et al. [67] utilized NH_4_OH to assess the effect of acidity on the formation of nanocrystalline alumina, observing a significant increase in the heat of reaction when varying the pH from 2.0 to 10.5, based on Equation (1). The presence of NH_4_NO_3_ has also been shown to increase the heat of reaction; this affects the physicochemical properties of the materials, notably the phase composition [68]. The use of HNO_3_ as an oxidant, on the other hand, has been deemed to be less effective on the combustion dynamics than NH_4_OH [61]; this agrees with our results, shown in Figure 6b. It is important to mention in this context that using other additives to modify the pH (e.g., KOH or H_2_SO_4_) will probably result in different combustion dynamics and would likely alter the physicochemical properties of the samples; this would be an interesting avenue for further investigation.

#### 2.4.2. XRD Measurements

The effect of pH on the crystalline phases of the samples produced at different pH values is depicted in Figure S18a and summarized in Table 5. In Region (I), β-NiMoO_4_ was prevalent, and the phase composition seemed to be hardly affected by pH change, as evidenced by the practically identical spectra between pH = 1 and the control sample (also see Table 5). However, by adding NH_4_OH to increase the pH, a clear and significant transformation in the phase composition occurred. At pH = 5, the presence of β-NiMoO_4_ faded, while that of α-NiMoO_4_ increased, in addition to a marked rise in the amount of NiO. At pH = 6, the content of β-NiMoO_4_ continued to decline along with MoO_3_ and NiO, while α-NiMoO_4_ was more pronounced. A further increase in pH did not seem to have a discernable impact on the phase composition except for an incremental increase in the amount of β-NiMoO_4_ at the expense of the α-phase (Table 5).

The effect of pH can be attributed to several factors. First, as seen in Figure 6b, the addition of HNO_3_ and NH_4_OH, both of which are oxidizing agents, to adjust the pH changed the dynamics of the combustion reaction, leading to a variation in T_ad_, a parameter that is indicative of the combustion rate. Apart from the combustion dynamics, another aspect where a pH adjustment is relevant relates to its effect on the complexing affinity of the fuel toward the metal ions present in the solution. The relation between the ability of the complexing agent to bind to metal ions and the pH of the medium has been extensively reported in the literature [12]. The β-phase was predominant when HNO_3_ was added for pH reduction, while the α-phase was dominant in the upper pH region where NH_4_OH was used. The effect of pH on phase structure has been elucidated previously by Junliang et al. [69], who evaluated the influence of pH on the phase composition of their synthesized powders. They suggested that the phase composition of barium hexaferrite could be changed from a multiphase mixture to a single-phase BaFe_12_O_19_ as the barium and iron ions complex fully with citric acid when pH increases. Studies linking the chelating activity of agar to pH are lacking in the literature but are much needed to fully understand the interplay between the pH, combustion temperature, and the complexing affinity of agar toward metal ions. Apart from the chelating and oxidizing effects, the change in pH could also induce a change in phase equilibria since the rise in pH promotes the formation of metal hydroxide, which precipitates and leads to the production of segregated phases, promoting the formation of α-NiMoO_4_, akin to the coprecipitation method [37]. The coprecipitation approach to produce NiMoO_4_ has been traditionally implemented using pH modifications (pH > 5) to induce the precipitation of Ni and Mo, leading to the predominant production of α-NiMoO_4_ [2].

**Figure 8:**
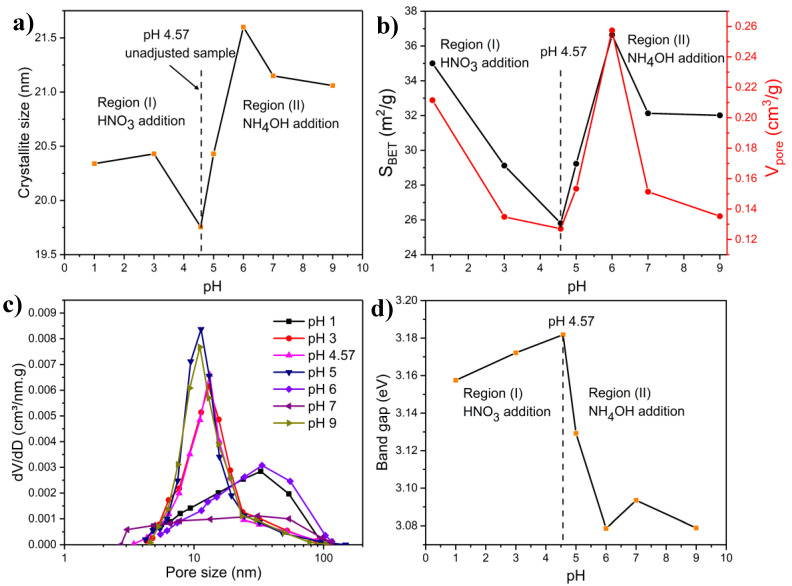
The effect of the precursor solution’s pH on the (**a**) crystallite size, (**b**) specific surface area and pore volume, (**c**) BJH pore size distribution, and (**d**) band gap of the synthesized NiMoO_4_ materials. Calcination temperature: 500 °C, calcination time: 6 h, and φ = 1.

Figure 8a shows the change in NiMoO_4_ crystallite size as a function of pH. The behavior can be ascribed to the influence of T_ad_ and evolved gas and is in accordance with the results in Figure 6b. In the low-pH region (pH ≤ 4.57), T_ad_ slightly increased as pH decreased, which contributed to the formation of larger crystallites. This was compensated by a significant increase in evolved gas. However, at a pH higher than that of the unadjusted sample (pH = 4.57), T_ad_ increased at a much higher rate, leading to higher comparative growth in the crystallite size.

Those results agree with a previous study by Duraz [70], who observed that the use of NH_4_NO_3_ in the synthesis of cobalt ferrite through the SCS method brings about a higher reaction temperature and an increase in the crystalline size. Similarly, Yue et al. [71] investigated the effect of pH on NiCuZn ferrite. They could obtain highly porous precursors with network structures at high pH and justified their observations by an increase in the combustion rate, resulting in larger crystals. The same conclusion was reached by Peng et al. [67], who also attributed the increased crystallite size to the high enthalpy of the combustion reaction in the high-pH region.

#### 2.4.3. FTIR Characterization

The FTIR spectra of the materials prepared at different pH values are displayed in Figure S18b. The plot reflects the phase change that took place as a result of altering the pH by adding HNO_3_ or NH_4_OH. This accords with the findings obtained from the XRD spectra (Figure S18a and Table 5). Indeed, in the region where HNO_3_ was added (Region I), an insignificant change in the spectra could be seen, except at pH = 3 where the band at 700 cm^−1^ was more pronounced. In this region, the characteristic bands of β-NiMoO_4_, at 880 and 800 cm^−1^, were fully developed. In Region (II), at pH = 5, it is noteworthy that the band at 650 cm^−1^ was reduced drastically, signifying structural changes in the material. Additionally, a notable decrease in the intensity of β-NiMoO_4_-related peaks was evident. At pH = 6, the β-NiMoO_4_-related peaks were diminished significantly, while a high-intensity peak at 595 cm^−1^ was formed, which, together with the band at 955 cm^−1^, signify the increased presence of α-NiMoO_4_, coinciding with the XRD results (Figure S18a, Table 5). At pH = 7, the spectrum still points to the prevalence of α-NiMoO_4_, accompanied by a minor presence of β-NiMoO_4_. It is noteworthy that a slight shift in the position of the peaks is apparent between pH 5–7, which signifies structural and morphological modifications. At pH = 9, the spectrum slightly changed compared to that of pH = 7, except for a minor increase in the peaks of β-NiMoO_4_, consistent with the XRD results (Figure S18a and Table 5).

#### 2.4.4. BET Measurements

N_2_ adsorption–desorption isotherms of the powders synthesized at different pH values are shown in Figure S19. All the isotherms correspond to Type III with H3 type hysteresis. The influence of the pH on the specific surface area and pore volume is portrayed in Figure 8b and seems to follow a similar trend as that observed in Figure 8a for the crystallite size, and therefore the results can be assessed using the same regions. In Region I, the amount of exhausted gas decreased significantly with an increase in pH, accompanied by a minor reduction in T_ad_. Consequently, the specific surface area decreased, which shows that the amount of evolved gas governed the trends of specific surface area and pore volume in this pH region. This is reasonable since gas generation elicits higher porosity, which helps increase the specific surface area. The surface area and pore volume reached a minimum at the control sample (pH = 4.57), where T_ad_ and the amount of generated gas were at a comparative minimum (Figure 6b). In Region (II), as the amount of evolved gas increased again (Figure 6b), the specific surface area and pore volume followed suit, despite an uptick in T_ad_. The specific surface area and pore volume attained a maximum at pH = 6. In addition to being influenced by the combustion dynamics, the existence of this maximum could be related to the change in the sample’s morphology, being predominantly nanorods at this pH (Figures S20 and S21). It is well-known that the specific surface area is governed by several factors, including particle size, particle morphology, surface texturing, and porosity. The large surface area and pore volume observed in this sample arose from the void spaces originating from the nanorod’s assembly. Beyond pH = 6, the specific surface area and pore volume declined again, mostly due to the considerably higher T_ad_, which reached ~2800 °C, bringing about severe agglomeration and tightly packed particles (Figure S20), in addition to morphological transformation in the produced nanostructures. As is shown in Figure S20 (Section 2.4.5), the nanorods at pH = 7 were larger than those at pH = 6 and thus had a smaller surface area and pore volume [72]. Therefore, it can be concluded that while T_ad_ has an important effect on the crystallite size and agglomeration, the amount of gas evolved contributes to higher porosity and has a more important effect on the surface area.

The effect of pH on the samples’ PSD is presented in Figure 8c. For pH = 1, a broad distribution was observed in the range of 4.8 and 53.8 nm due to the high gas generation rate at low pH, as seen in Figure 6b. As the pH increased, the shape of PSD narrowed (spanning from 4.6 to 23.7 nm), and the mean peak size shifted to ~13 nm. An identical profile was observed with the control sample. In Region (II), at pH = 5, the mean value was ~11.3 nm, with a range of 4.8–24.5 nm. At pH = 6 and 7, where the morphology of the materials changed (refer to Figures S20 and S21), the PSD underwent a sharp transformation, shifting again to a broad distribution with an average peak at 33.5 nm in a range of 5.4–96.7 nm. This was due to a drastic change in morphology seen at pH = 6 and 7 where nanorods are predominantly present. In fact, the PSD at both pH = 6 and 7 closely resembled each other since both constitute nanorods, but the PSD of the sample prepared at pH = 7 exhibited a higher mean peak size with pores distributed over a wide pore size range. At pH = 9, the distribution profile changed again, resembling that of the samples prepared in the pH region of 3–5, with a pore range spanning from 4.5 nm to 23.5 nm.

#### 2.4.5. Microscopy Characterization

The morphology and particle size of the samples prepared at different pH values are illustrated in Figures S20 and S21. For pH = 1, the particle size range was 15–30 nm, while the morphology was of a fluffy type and exhibited visibly large pores distributed over a wide size range, in accordance with the results in Figure 8b,c. At pH = 3, the pore size range and the morphology of the sample were similar to those at pH = 1, notwithstanding the apparent decrease in the pore size, also evidenced in Figure 8c. For the control sample (pH = 4.57, portrayed as the sample calcinated at 500 °C in Figure 4), the morphology was comparable to the samples prepared at the lower pH, with a particle size distribution of 20–40 nm. In Region (II), at pH = 5, the morphology of the particles was almost identical to that of the control sample, but with a scant appearance of nanorods. At pH = 6, the sample exhibited a large presence of agglomerated nanorods with a diameter and length of 25–35 nm and 150–250 nm, respectively. The synthesis of NiMoO_4_ nanorods has been reported previously by several researchers [18], although none used the SCS method. Notably, the hydrothermal method was used in the pH range of 5–6 to produce α-NiMoO_4_ nanorods similar in size and shape to what we observed in the current study [73]. A further increase in pH to 7 led to the preponderance of nanorods, which were of a less agglomerated nature and higher definition than those seen at pH = 6, with a diameter of 25–40 nm and length of 350–450 nm. The presence of nanorods in the pH range of 5–7 means that the nucleation of the particles occurred over a longer period in this range than at lower pH, indicating a different kind of particle growth compared to the other samples prepared at different pH. Surprisingly, when the pH increased slightly to pH = 8 (not shown), the morphology constituted highly aggregated, tightly packed microparticles akin to knitting balls with a primary particle size range of 15–35 nm and an irregular shape. At pH = 9, a more significant degree of agglomeration was seen, as the particles formed large microspherical aggregates.

The considerable aggregation associated with high pH values is a consequence of the extreme adiabatic temperature observed during the combustion reaction, as demonstrated in Figure 6b. Pathak et al. [68] studied the influence of pH on the formation of nanocrystalline alumina powder using citric acid as fuel. The particle morphology, in their work, changed from plate-like flake structures (observed at a low pH of 2) to desegregated fine particulates at pH = 10. They attributed the findings to a change in the reaction’s exothermicity that resulted from adding NH_4_OH to the solution, which intensified the combustion reaction. Additionally, the change in morphology could also be linked to the variation in the zeta potential of agar, which is a function of pH [74]. Interestingly, the shift in morphology to nanorods occurred close to the isoelectric point of agar, which was reported previously to occur at pH = 5.53 [75]. The relationship between the zeta potential and the morphology is likely related to their effect on the chelating affinity of agar with cations, which, in turn, depends on the pH [76], but more studies are warranted to better elucidate this aspect. The addition of ammonia and the resulting highly alkaline media can also impact the hydrolysis–condensation reaction, affecting the material’s morphology. High pH values in the precursor solution have been reported to form large agglomerates in other studies where an increase in pH induced the precipitation of metal hydroxides, leading to pronounced aggregation during the combustion process [77]. Additionally, in a different study [78], it was revealed that the samples prepared at low pH exhibited porous and loosely bonded particles, while those synthesized at higher pH (8, 9) were characterized with hard agglomeration, in agreement with our findings.

SAED patterns were recorded for selected samples, as shown in Figure S21. At pH = 1, the material was highly polycrystalline. As the morphology of the material changed predominantly to nanorods (pH = 6), a reduced intensity in the rings was observed, albeit with persistent polycrystallinity. At pH = 9, the SAED pattern was similar to that of pH = 1.

#### 2.4.6. Band Gap Measurements

The influence of pH on the band gap (obtained from the Tauc plots in Figure S22) is shown in Figure 8d. Although the band gap values of all the samples prepared at different pH existed within a narrow range, the materials synthesized at low pH (Region I) exhibited, on average, a larger band gap than those obtained at a higher pH. The phase composition plays a vital role in governing the band gap. The β-phase, prevalent in the low pH region, exhibited a larger band gap than that of the α-phase, as mentioned in Section 2.1.5, based on computational studies performed elsewhere [49]. The change in band gap can also be ascribed to the variation in morphologies with varying pH. Namely, the reduction in band gap in the pH range of 5–7 can be explained by the appearance of nanorods in this pH region. This agrees with the findings of Musa et al. [79], who reported a change in band gap values when the material morphology (zinc oxide nanomaterials) changed from nanoparticle to nanorods, attributing this occurrence to the quantum confinement effect. Furthermore, Feng et al. [80] stated that a marked reduction in the band gap of TiO_2_ could be obtained by altering the morphology of the structure. The slight increase in band gap seen in our study, between pH = 6 and 7 (both of which showed the presence of nanorods), was likely due to the difference in their length and diameter. This is in line with the work of Li [48], who developed a thermodynamic model to calculate the band gap of nanorods with varying lengths and diameters, noting that the band gap was influenced by the dimensions of the material.

### 2.5. Effect of Fuel Content

Of all the variables that govern the combustion reaction in the SCS method, the effect of fuel content (i.e., fuel-to-oxidant ratio (φ)) is arguably the most influential parameter. Many researchers have investigated the effect of this parameter on the physicochemical characteristics of nanostructured materials produced through the SCS method [61]. It has been noted that properties such as specific surface area, crystallinity, phase, morphology, and band gap are dependent on the fuel content [61]. Additionally, from a sustainability point of view, it is important to determine the proper fuel-to-oxidant ratio since a reduced amount of fuel is desirable for an efficient, cost-effective system.

In the following section, NiMoO_4_ samples were prepared using different fuel-to-oxidant ratios (φ). To the best of our knowledge, this aspect has not yet been studied in any SCS-related work on NiMoO_4_. It is also noteworthy that in many literature studies involving the SCS method, a missing element was observed, related to investigating the impact of fuel absence on the properties of the products. Therefore, in the following section, we report on the samples produced with different agar content, in addition to a control sample devoid of agar, to better elucidate the role of the fuel on the physicochemical properties of the products. The NiMoO_4_ samples discussed in the following text were prepared by calcination at 500 °C for a period of 6 h.

#### 2.5.1. Combustion Parameters

The effect of the fuel content (φ) on T_ad_ and the amount of evolved gas is presented in Figure 6c. As shown in the figure, in the fuel-lean region (φ < 1), as φ increased, T_ad_ and the evolved gas (N_2_, water vapor, and NH_3_) increased, albeit at different rates. Beyond the stoichiometric ratio (φ = 1), in the fuel-rich region, T_ad_ still increased but at a lower rate than that seen in the fuel-lean region, while the amount of the exhausted gas rose at a considerably steeper rate. The increase in gas generation and T_ad_ in fuel-rich systems has been established in prior studies using thermodynamic modeling [81]. While the above discussion portrays the thermodynamically calculated combustion parameters, other aspects are also relevant when varying the fuel content, namely the flame type and the combustion duration. At a small φ, the duration of the combustion reaction is short (slow-burning rate) with a smoldering flame accompanied by a mild combustion temperature (usually below 1000 °C) [82]. As the fuel amount approaches stoichiometry (φ = 1), the combustion type shows an incandescent appearance (flame glow) of a short duration; however, as the amount of fuel further rises (fuel-rich), the combustion type still shows a flame but with a markedly longer duration [63]. Those considerations hold important implications for the physicochemical properties of the samples, as will be seen next.

#### 2.5.2. XRD Measurements

The influence of the fuel content on the structure of NiMoO_4_ is shown in Figure S23a. The sample prepared without fuel (φ = 0) predominantly comprises the α-NiMoO_4_ phase (Table 6). In fact, in the absence of agar (φ = 0), the formation of nanostructures occurred by co-precipitation, which has been, traditionally, the most commonly used and reported method to produce α-NiMoO_4_ [2]. A small amount of the β-phase in this sample was detected since the powder was cooled rapidly at the end of the calcination step, in accordance with other reports [9]. When a small fuel amount was utilized (φ = 1/3), the phase changed dramatically to the β-phase (Table 6), as evidenced by the significant increase in the characteristic peak of β-NiMoO_4_ (26.6°, Figure S23a). This illustrates the importance of the combustion reaction in the formation of β-NiMoO_4_. With a higher fuel amount (φ = 1/2), the content of the β-phase increased to ~65 wt.% with a concurrent drop in the amount of α-NiMoO_4_ (Table 6). As the amount of the fuel increased further (φ ≥ 1), the amount of β-NiMoO_4_ reached a maximum at φ = 1 (~73 wt.%) with a simultaneous decline in the content of the α-phase, beyond which the phase composition changed slightly. The results presented here show that at a minimum, a stoichiometric fuel-to-oxidant ratio (φ = 1) is required to maximize the amount of the β-NiMoO_4_ phase in the product under the experimental conditions employed in this study (Table 6).

The effect of fuel content on the phase structure was illustrated previously by various authors. For example, Guo et al. [63] showed that the ratio of the monoclinic to the tetragonal phase of ZrO_2_ exhibits a volcano trend, culminating at the stoichiometric fuel-to-oxidant ratio. In our study, the fact that the increase in the amount of fuel influenced the phase structure (proliferation of the β-phase) could be attributed to both the high T_ad_ associated with the combustion reaction and the complexing affinity of agar toward metal cations. As mentioned previously [22], the high combustion temperature during the combustion reaction induced an excess of Ni in the lattice structure of NiMoO_4_, which led to stabilizing β-NiMoO_4_ at room temperature. The small drop in the β-phase with high fuel content is likely due to the weakening in the combustion reaction since at high φ the auto combustion reaction becomes slow and loses intensity [81]. Another controlling factor in the increase in the β-phase with higher fuel content can be attributed to the complexing affinity of agar. Agar contains functional groups, including -OH functional groups and acidic-side groups such as sulfate and pyruvate that could help bind to metal ions and inhibit precipitation and phase segregation of the metal species, thus contributing to phase changes [83].

**Figure 9:**
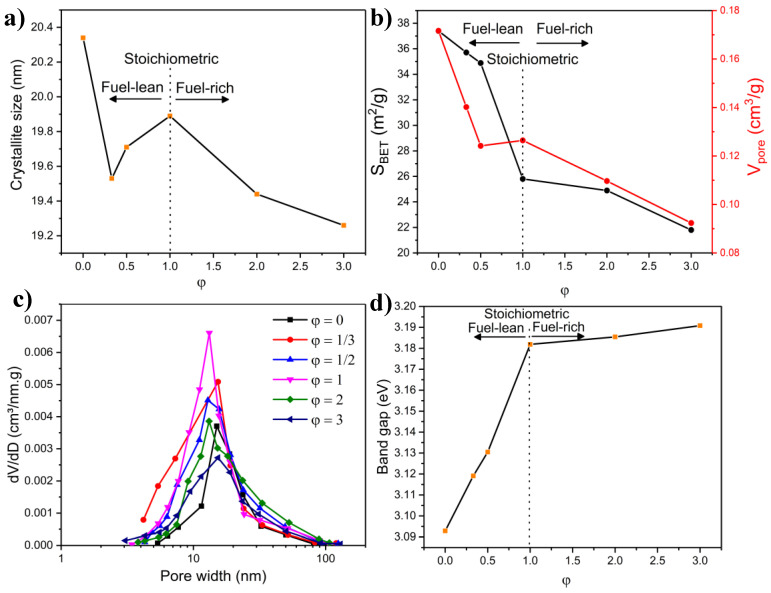
The effect of fuel-to-oxidant ratio (φ) on the (**a**) crystallite size, (**b**) specific surface area and pore volume, (**c**) BJH pore size distribution, and (**d**) band gap of the synthesized NiMoO_4_ materials. Calcination temperature: 500 °C, calcination time: 6 h, and pH = 4.57.

The crystallite size dependence on the fuel-to-oxidant ratio is portrayed in Figure 9a. The sample in a fuel-deprived system (φ = 0) presented the largest crystallite size. When employing the fuel (agar) in the synthesis method, the crystallite size decreased initially in the fuel-lean region (φ = 1/3) due to the mild T_ad_ and the high generation rate of gases (refer to Figure 6c), both of which yielded small crystals. At φ = 1/2, the crystallite increased as T_ad_ ramped up at a higher rate than that of gas generation; the increase in crystal size reached a maximum at the stoichiometric value (φ = 1). In the fuel-rich region, as the rate of generated gas became more predominant than that of T_ad_, the crystallite size declined again due to the higher generation of gaseous products at higher fuel amounts, which helps to cool the combustion front and impede the crystal growth.

There is little agreement in the literature related to the dependence of the crystallite size on the fuel content. While some researchers reported an increase in crystallite size with higher φ due to an elevated T_ad_, others mentioned that a fuel increase brought about a decline in crystallite size due to more vigorous gas generation. High T_ad_ causes a growth in the crystallite size by sintering and agglomeration, whereas released gaseous products impact the cooling rates during the combustion reaction, both factors being competing forces [37]. For instance, Toniolo et al. [84] revealed that the combustion temperature in the formation of Co_3_O_4_ increases with higher fuel content (urea and glycine), leading to a growth in the specific surface area and crystallite size. In contrast, Masoudpanah et al. [37] showed that the crystallite size of their synthesized CoFe_2_O_4_ nanoparticles decreases with excessive use of fuel (poly(vinyl-pyrrolidone)) due to the large volume of exhausted gases that mitigates any temperature increase from the combustion reaction. In our opinion, the fuel type is a highly influential factor because fuels are characterized by varying functional groups, side chains, and heat of combustions, all of which endow different fuels with varying affinity in emitting gases depending on their molecule structure and functional groups [37].

#### 2.5.3. FTIR Characterization

The FTIR spectra (Figure S23b) confirm that in the absence of the fuel (φ = 0), the characteristic peaks specific to β-NiMoO_4_ (885 and 805 cm^−1^) were poorly noticeable, which agrees with the XRD results presented in Figure S23a and Table 6. Upon using a small amount of fuel (φ = 1/3), the intensity of the β-NiMoO_4_ characteristic peaks increased, signifying an increased content of this phase, while the peaks at 930 and 603 cm^−1^ experienced a blue shift, indicating the change in the structure of the material. As φ was raised, the β-NiMoO_4_ peaks were amplified and became fully developed at φ = 1/2, in accordance with the conclusions drawn from Figure S23a and Table 6. For φ = 2 and 3, in addition to the other residual peak at 1630 cm^−1^, a peak corresponding to a precursor residual element is apparent at 2340 cm^−1^ due to the increased presence of agar in the precursor, while the remaining peaks were remarkably identical to those seen with φ = 1. For an extremely fuel-rich combustion system, the combustion reaction takes place without fully combusting the fuel, which could result in the charring of the product, leaving behind residual carbon [61].

#### 2.5.4. BET Measurements

The N_2_ adsorption–desorption isotherms used to determine the specific surface area, pore volume, and pore diameter at varying fuel-to-oxidant ratios (φ) are shown in Figure S24. All the isotherms are of Type III with H3 hysteresis, indicating the mesoporous nature of the samples. The evolution of the specific surface area with φ is portrayed in Figure 9b, which shows that the specific surface area and pore volume were higher in the fuel-lean region and decreased gradually as the amount of fuel increased. In the fuel-lean region (φ < 1), the heat generated was not comparatively elevated. This reduced the reaction rate, combustion temperature, and evolved gas (Figure 6c), yielding smaller particles (SEM and TEM images in Figures S25 and S26) and higher specific surface areas. An increase in the agar content entailed a drop in the specific surface area and pore volume (Figure 9b), accompanied by a phase transformation to β-NiMoO_4_ (Table 6). The change in the specific surface area, seen in Figure 9b, can be explained by referring to Figure 6c. It is apparent that as more fuel was used, T_ad_ and the amount of evolved gas increased, although at different rates, whereby T_ad_ rose at a higher rate in the fuel-lean region and slowed down as more agar was used. The volume of evolved gas showed the opposite trend. The steep increase in T_ad_ in the fuel-lean region led to a considerable reduction in the specific surface due to crystal growth, sintering, and agglomeration. At the same time, the amount of generated gas partially helped to dampen this effect. In the fuel-rich region, the increase in both T_ad_ and gas volume occurred at a lower rate, exerting a smaller impact on the surface area and pore volume. The rate of decrease in a specific surface area in the fuel-rich region (φ > 1) was lower than that seen in the fuel-lean region, which confirms the importance of the amount of gas evolved in the combustion reaction needed to neutralize the effects of high T_ad_.

Our results agree with the findings of other researchers, notably Pourgolmohammad et al. [37], who studied the effect of fuel content on the surface area of CoFe_2_O_4_ nanoparticles with different fuels (glycine, urea, citric acid), noting that the surface area of their samples decreased with increasing φ until leveling off in the fuel-rich region, asserting that the evolved gas helps in normalizing the elevated T_ad_ seen at high temperatures. Nevertheless, despite many studies, the effect of fuel content on the structure and texture of products is still not clear as different and often contradictory trends, explaining the change in particle size with fuel content, have been reported in the literature. For example, some authors noted that an increase in fuel contributed to a large particle size, while others have presented a different view whereby a volcano-type trend was observed [63]. This, yet again, points to the intricate nature of the combustion system that is often highly dependent on the fuel type.

The pore size distribution of the NiMoO_4_ samples, produced at different fuel-to-oxidant ratios, is shown in Figure 9c. The PSD of the samples exhibited highly similar profiles with slight differences in the mean peak size. In the absence of agar (φ = 0), PSD showed a narrow size distribution with a mean size at 15.5 nm, which declined slightly to 13.2 nm upon adding a small amount of agar (φ = 1/2) and remained almost unchanged at the stoichiometric fuel-to-oxidant ratio (φ = 1). Beyond the stoichiometric ratio, the distribution became broader, while the mean pore size increased to ~15.7 nm in the fuel-rich region (φ = 3). The dependence of the pore size on the fuel-to-oxidant ratio stems from the fact that the greater the amount of fuel yield used, the higher the volume of generated gas produced, which penetrates through the combusted material, yielding large pores and wider PSD, as shown elsewhere [85].

#### 2.5.5. Microscopy Characterization

The morphologies of the samples prepared with different fuel-to-oxidant ratios (φ) are shown in Figures S25 and S26. When no agar was used (φ = 0), the samples exhibited irregularly shaped nanostructures and a broad particle size distribution (35–85 nm) without the foamy, sponge-like texture associated with materials produced via SCS. This could be explained by the role that agar plays as a fuel, which upon combustion leads to a high gas generation rate, endowing the material with a foamy characteristic [22]. Additionally, the lack of effective complexing leads to cation segregation, uncontrolled precipitation, and random-sized and shaped particles. When a small amount of agar was used (φ = 1/3), the morphology showed two distinct features: (i) a spongy, porous texture derived by the combustion reaction, with a more uniform particle distribution in the range of 15–30 nm and (ii) quasi-spherical shape and irregular particles. This shows that in the extremely fuel-deficient system (φ = 1/3), the amount of agar was not sufficient to produce homogeneously shaped structures. It is noteworthy that the large number of -OH moieties found in agar help in binding to metal ions and contribute to the formation of smaller-sized particles. The particle size and agglomeration increased at higher agar content (20–40 nm and 30–55 nm for the stoichiometric mixture (φ = 1) and fuel-rich (φ = 3) materials, respectively); this observation arises from the increased T_ad_ observed at higher φ, as seen in Figure 6c. The increase in particle size resulted in a decline in the specific surface area, as shown in Figure 9b.

#### 2.5.6. Band Gap Measurements

The Tauc plots in Figure S27 allowed the determination of the band gaps for the materials prepared at different φ (Figure 9d). The sample synthesized without agar (φ = 0) exhibited the smallest band gap, while in the fuel-lean region (φ < 1), the band gap seemed to rise in a linear fashion. In the fuel-rich region (φ ≥ 1), the band gap increased only slightly with a higher agar content. As was mentioned previously, the fuel-lean region is associated with major changes in the phase composition of the products (marked increase in the content of the β-phase at the expense of the α-phase), crystallite size, and surface area (Figure 9), all of which contribute to the value of the band gap. In the fuel-rich region where minor changes were seen with respect to the physicochemical properties, the band gap varied within a limited range. The large nature of the band gaps in the fuel-rich region agrees with the findings of Shaat et al. [50], who confirmed, using the SCS approach to produce ZnO nanoparticles, that an increase in fuel leads to an increase in the band gap.

## 3. Materials and methods

### 3.1. Material Synthesis

Using the production method outlined in our previous study [22], the influence of several experimental conditions on the physicochemical/structural/topographical properties of the synthesized nanostructured NiMoO_4_ was assessed. Briefly, the production of the nanostructured NiMoO_4_ materials discussed in the current paper was based on the solution combustion synthesis (SCS) and the principles of propellant chemistry, where agar was utilized as an organic fuel and Ni(NO_3_)_2_ as both a Ni source and oxidizing agent, while ammonium heptamolybdate served as the Mo source. Unless otherwise indicated, a stoichiometric fuel-to-oxidant ratio (φ) was used, corresponding to the condition where the ratio of the net oxidizing valency of the metal nitrate to the net reducing valency of the fuel is unity. In addition, with a stoichiometric fuel-to-oxidant ratio, the combustion reaction does not require any molecular oxygen to be initiated (Equations (S4) and (S5)). Other fuel-to-oxidant ratios investigated in this work were: φ = 1/3, 1/2, 1, 2, and 3. To prepare the stoichiometric sample, a Ni-containing solution of 0.01 moles of nickel nitrate (Ni(NO_3_)_2_) × 6H_2_O, Sigma-Aldrich, St. Louis, MO, USA, 97% purity) and a Mo-containing solution of 0.0014 moles of ammonium molybdate tetrahydrate ((NH_4_)_6_Mo_7_O_24_ × 4H_2_O, Fisher Scientific, Hampton, NH, USA, 99% purity) were prepared in ultrapure water. The Ni and Mo solutions were mixed in separate beakers in 30 mL of water for 30 min to ensure adequate homogeneity, after which the Mo-containing solution was added to that of Ni. The effect of sample composition was assessed by varying the Ni/Mo ratio (% Ni: 0, 20, 40, 60, 80, and 100). The pH of the resulting solution was 4.57, which was modified (pH = 1, 3, 5, 6, 7, and 9) when the effect of pH was investigated (pH changes in the precursor solution were executed using diluted HNO_3_ (68–70%, Fisher Scientific, Hampton, NH, USA) and NH_4_OH (28–30%, Fisher Scientific, Hampton, NH, USA). Thereafter, the mixture was placed on a hot plate and heated to a set temperature of 95 °C. Agar (fuel) was added to this solution as the temperature was ramping up. When agar was dissolved in the solution (15 min), the solution was removed to be cooled at ambient conditions. The solution was dried in a convection oven at 110 °C for 24 h to remove the water content. Calcination was then performed in a muffle furnace at varying temperatures (300, 400, 500, 600, 700, 900, and 1100 °C) for different durations (1, 5, 20, 60, 360, 720, and 1440 min), at the end of which the product was cooled slowly to room temperature, mildly crushed with a pestle to break up the clusters, and then stored in glass vials.

### 3.2. Characterization Techniques

X-ray diffraction (XRD) was employed to determine the crystallinity, identify the prevalent crystal phases, and compute the grain size, using a Bruker D8 DISCOVER X-ray diffractometer with a two-dimensional VÅNTEC-500 detector and CuK (λ = 1.54056 Å) source of radiation with a tube voltage and current of 40 kV and 20 mA, respectively. An estimate of the phase composition present in the samples was carried out using a semi-quantitative phase analysis procedure based on the EVA software. The semi-quantitative analysis was performed, employing the pattern’s relative heights and I/I_cor_ values, where I/I_cor_ refers to the ratio between the intensities of the strongest line of the compound of interest and the strongest line of corundum (cor). The crystallite size was obtained from the XRD spectra using the Scherrer formula and represents the average crystallite size of all the phases determined in the phase composition analysis.

N_2_ adsorption–desorption measurements (−196 °C) were conducted using a Micromeritics Tristar 3000 BET analyzer to determine the total surface area, pore size distribution, and pore volume of the synthesized materials. Before the analysis, the samples were degassed under vacuum for 12 h at 120 °C. The Barrett–Joyner–Halenda (BJH) model and the Brunauer–Emmett–Teller (BET) methods were applied to determine the pore size and BET surface area, respectively. The pore size was computed using the adsorption and desorption branches of the isotherm. The pore size distributions were calculated from the data of the desorption branch of the isotherm using the BJH method for the pore volume and the Kelvin equation for the pore size. All reported data vary within ± 5% based on repeated analysis. The BET surface area will be referred to as the “specific surface area” in the entirety of the text.

Optical properties were measured by photoluminescence spectroscopy utilizing a 266 nm excitation laser and a UV/Vis spectrometry (Thermo Scientific Evolution 300, Thermo Fisher Scientific, Waltham, MA, USA) using green light to generate diffused reflectance. KBr was used as a diluent for the measured samples with a KBr-to-sample ratio of 90:10 wt.%.

The morphology and the particle size of the materials were studied using field emission scanning electron microscopy (FE-SEM, Hitachi S-8000, Tokyo, Japan) and transmission electron microscopy (FEI Tecnai G2 F20 with 200 kV, Hillsboro, OR, USA). Elemental mapping was carried out using an EDS detector (XMax 80 mm^2^ Oxford Instruments, Abingdon-on-Thames, UK), and the data was processed on the Aztec software.

FTIR spectra were recorded using a Bruker Alpha FTIR in the wavenumber range of 400–4000 cm^−1^.

The heat of formation of agar was obtained by Parr 6400 calorimeter using 243 g of agar as the input weight.

Inductively coupled plasma optical emission spectrometry (ICP-OES) was performed using the Thermo Scientific iCAP 6500 system (Thermo Fisher Scientific, Waltham, MA, USA) to determine the actual bulk compositions of the materials prepared at different Ni/Mo atomic ratios.

## 4. Conclusions

The solution combustion synthesis method was used to produce NiMoO_4_ samples with various physicochemical properties. By using extensive physical characterization methods, we showed that the structural characteristics of the synthesized materials can be tailored depending on the initial experimental conditions. The change in calcination temperature had an impact on all the investigated properties, most notably on the phase composition, where the β-phase was maximized at 500 °C (in Part II of this manuscript series, it will be shown that the predominance of the β-phase in NiMoO_4_ plays a major role in the material’s electrocatalytic activity in the hydrogen evolution reaction). The change in the calcination time exhibited a mild influence on the investigated properties of the materials, although short calcination periods were necessary to stabilize the phase composition of the samples.

Concerning the impact of Ni/Mo atomic ratio in NiMo-oxides, thermodynamic computations showed that as the amount of Ni increased in the oxide compound, the combustion reaction became less intense, resulting in an increased specific surface area and porosity and a reduced crystallite size. The pH of the precursor solution was also found to exhibit an important influence, where low pH conditions favored the formation of β-NiMoO_4_, while alkaline conditions allowed the formation of the α-phase. The effect of the amount of agar was also explored, and we showed that in the absence of the fuel, the produced material was characterized by densely packed particles, poor porosity, and consisted mainly of α-NiMoO_4_. Conversely, at higher fuel amounts, the samples attained a foamy and porous texture, while the phase composition increasingly shifted to the β-NiMoO_4_ phase with higher fuel-to-oxidant ratios, until leveling off beyond φ = 1.

## Figures and Tables

**Table 1:** The effect of calcination temperature on the phase composition of NiMoO_4_ samples. Calcination time: 6 h, pH = 4.57, and φ = 1.

Temperature (°C)	Phase Composition (wt.%)				
	α-NiMoO_4_	β-NiMoO_4_	MoO_2_	MoO_3_	NiO
300	22.4	22.7	46	1.8	7.1
400	41.9	40.7	0	12.3	5.1
500	19.6	72.8	0	4.1	3.5
600	73.7	23	0	2.2	1
700	90.9	7.4	0	1.4	0.3
900	100	0	0	0	0
1100	100	0	0	0	0

**Table 2:** The effect of calcination time on the phase composition of NiMoO_4_ samples. Calcination temperature: 500 °C, pH = 4.57, and φ = 1.

Time (min)	Phase Composition (wt.%)				
	α-NiMoO_4_	β-NiMoO_4_	MoO_2_	MoO_3_	NiO
As-combusted	22.1	21.9	44.4	4.4	7.1
5	37.9	44.8	0	10.4	6.9
20	24.7	55.5	0	7	3.4
60	26.9	62.5	0	5.8	4.8
360	19.6	72.8	0	4.1	3.5
720	26.3	67.7	0	2.8	3.1
1440	41.4	51	0	2.5	5.1

**Table 3:** Nominal and actual compositions of the synthesized Ni*_x_*Mo_1-*x*_-oxides, determined by ICP-OES analysis.

Nominal	Observed
MoO_3_	MoO_3_
Ni_0.20_Mo_0.80_O_x_	Ni_0.20_Mo_0.80_O_x_
Ni_0.40_Mo_0.60_O_x_	Ni_0.40_Mo_0.60_O_x_
Ni_0.50_Mo_0.50_O_x_	Ni_0.51_Mo_0.49_O_x_
Ni_0.60_Mo_0.40_O_x_	Ni_0.59_Mo_0.39_O_x_
Ni_0.80_Mo_0.20_O_x_	Ni_0.80_Mo_0.20_O_x_
NiO	NiO

**Table 4:** The phase composition of Ni*_x_*Mo_1-*x*_ oxide (0 ≤ *x* ≤ 1) samples. Calcination temperature: 500 °C, calcination time: 6 h, pH = 4.57, and φ = 1.

Composition (*x* in Ni_*x*_Mo_1-*x*_-oxide)	Phase Composition (wt.%)				
	α-NiMoO_4_	β-NiMoO_4_	MoO_2_	MoO_3_	NiO
0	0	0	0	100	0
0.2	25.9	4.5	0	69.6	0
0.4	44.4	26.8	0	27.7	1.1
0.5	19.6	72.8	0	4.1	3.5
0.6	19.2	70.3	0	2.7	7.8
0.8	–	–	–	–	–
1	0	0	0	0	100

**Table 5:** The effect of the precursor solution’s pH on the phase composition of NiMoO_4_ samples. Calcination temperature: 500 °C, calcination time: 6 h, and φ = 1.

pH	Phase Composition (wt.%)				
	α-NiMoO_4_	β-NiMoO_4_	MoO_2_	MoO_3_	NiO
1	22.4	73.3	0	3.2	1.1
3	21.3	74.2	0	2.7	1.8
4.57	19.6	72.8	0	4.1	3.5
5	27.8	58.7	0	3.9	9.6
6	79.6	15.6	0	3.2	1.6
7	75	20.8	0	1.5	2.7
9	69.1	28.5	0	1.1	1.4

**Table 6:** The effect of the precursor solution’s pH on the phase composition of NiMoO_4_ samples_._ Calcination temperature: 500 °C, calcination time: 6 h, and pH = 4.57.

Fuel-to-Oxidant Ratio φ	Phase Composition (wt.%)				
	α-NiMoO_4_	β-NiMoO_4_	MoO_2_	MoO_3_	NiO
0	85.9	13.1	0	0.7	0.3
1/3	39.8	56	0	2.7	1.8
1/2	30.3	64.7	0	3.5	1.4
1	19.6	72.8	0	4.1	3.5
2	24.8	71.3	0	2.7	1.2
3	26.9	69.6	0	2.5	1

## Data Availability

Data are available from the authors upon request.

## Supplementary Materials

The following supporting information can be downloaded online. Figure S1: SEM image showing the NiMoO_4_ sample calcined at 300 °C and its EDX analysis in the mapping mode, depicting the distribution of Ni, Mo, and O in the sample; the presence of Al and Cu in the EDX spectra was due to the deposition of the sample directly on the SEM aluminum stud, before imaging. No carbon was detected in the sample. Calcination time: 6 h, pH = 4.57, and φ = 1. Figure S2: Nitrogen adsorption–desorption isotherms of NiMoO_4_ samples produced at different calcination temperatures. Calcination time: 6 h, pH = 4.57, and φ = 1. Figure S3: TEM images of NiMoO_4_ samples produced at different calcination temperatures. Calcination time: 6 h, pH = 4.57, and φ = 1. Figure S4: Tauc plots derived from the UV-Vis absorption spectra of NiMoO_4_ samples produced at different calcination temperatures. Calcination time: 6 h, pH = 4.57, and φ = 1. Figure S5: (a) XRD patterns and (b) FTIR spectra of NiMoO_4_ samples produced during different calcination times; (α) α-NiMoO_4_, (β) β-NiMoO_4_, (*): MoO_2_, (♦) MoO_3_, and (Δ) NiO. Calcination temperature: 500 °C, pH = 4.57, and φ = 1. Figure S6: SEM image showing the as-combusted NiMoO_4_ sample and the EDX analysis in the mapping mode depicting the distribution of Ni, Mo, and O in the sample; the presence of Al and Cu in the EDX spectra was due to the deposition of the sample directly on the SEM aluminum stud, before imaging. No carbon was detected in the sample. Calcination temperature: 500 °C, pH = 4.57, and φ = 1. Figure S7: Nitrogen adsorption–desorption isotherm of NiMoO_4_ samples produced during different calcination periods; the plot labeled “as-combusted” refers to the sample removed immediately from the furnace after the combustion reaction and the other isotherms denote samples subjected to different calcination periods (5 min, 60 min, 360 min, 720 min, and 1440 min). Calcination temperature: 500 °C, pH = 4.57, and φ = 1. Figure S8: Representative SEM images illustrating the effect of calcination time on the surface morphology of NiMoO_4_ samples. Calcination temperature: 500 °C, pH = 4.57, and φ = 1. Figure S9: TEM images of the as-combusted NiMoO_4_ sample and the one calcined for 24 h. Calcination temperature: 500 °C, pH = 4.57, and φ = 1. Figure S10: Tauc plots derived from the UV-Vis absorption spectra for the NiMoO_4_ samples produced during different calcination periods. Calcination temperature: 500 °C, pH = 4.57, and φ = 1. Table S1: Relevant thermodynamics data used in the computation of adiabatic temperature. Figure S11: (a) XRD patterns and (b) FTIR spectra of Ni*_x_*Mo_1-*x*_-oxide (0 ≤ *x* ≤ 1) samples; (α) α-NiMoO_4_, (β) β-NiMoO_4_, (♦) MoO_3_, and (Δ) NiO. Calcination temperature: 500 °C, calcination time: 6 h, pH = 4.57, and φ = 1. Figure S12: Nitrogen adsorption–desorption isotherms of Ni*_x_*Mo_1-*x*_-oxide (0 ≤ *x* ≤ 1) samples (*x*: 0, 0.2, 0.4, 0.6, 0.8, 1). Calcination temperature: 500 °C, calcination time: 6 h, pH = 4.57, and φ = 1. Figure S13: Representative SEM images illustrating the surface morphology of Ni*_x_*Mo_1-*x*_ oxide (0 ≤ *x* ≤ 1) samples. Calcination temperature: 500 °C, calcination time: 6 h, pH = 4.57, and φ = 1. Figure S14: TEM images and SAED patterns of Ni*_x_*Mo_1-*x*_-oxide (0 ≤ *x* ≤ 1) samples (*x*: 0, 0.2, 0.4, 0.6, 0.8, 1). Calcination temperature: 500 °C, calcination time: 6 h, pH = 4.57, and φ = 1. Figure S15: Low-magnification SEM image of the material with Ni:Mo of 80:20 (*x* = 0.8). The image shows the high porosity of the material stemming from the low adiabatic temperature and vigorous gas volume generated during the combustion reaction. (Calcination temperature: 500 °C, calcination time: 6 h, pH = 4.57, and φ = 1). Figure S16: Tauc plots derived from the UV-Vis absorption spectra of Ni*_x_*Mo_1-*x*_-oxide (0 ≤ *x* ≤ 1) samples (*x*: 0, 0.2, 0.4, 0.6, 0.8, 1). Calcination temperature: 500 °C, calcination time: 6 h, pH = 4.57, and φ = 1. Figure S17: Characterization of commercial NiMoO_4_, procured from Alfa Aesar; (a) XRD pattern and the corresponding indexed standard that best match the observed spectrum, (b) nitrogen adsorption–desorption isotherm used to determine the BET surface area, pore volume, and pore size distribution, (c) pore size distribution obtained using the Kelvin equation and BJH model, (d) Tauc plot obtained from the UV-Vis absorption spectrum to calculate the band gap, and (e) representative SEM images taken at a different magnification, showing the presence of nanorods with a diameter range of 70–120 nm and length range of 300–450 nm. Figure S18: (a) XRD patterns and (b) FTIR spectra of NiMoO_4_ samples produced at different precursor solution’s pH; (α) α-NiMoO_4_, (β) β-NiMoO_4_, (♦) MoO_3_, and (Δ) NiO. Calcination temperature: 500 °C, calcination time: 6 h, and φ = 1. Figure S19: Nitrogen adsorption–desorption isotherms of NiMoO_4_ samples produced at different precursor solution’s pH (1, 3, 4.57, 5, 6, 7, 9). Calcination temperature: 500 °C, calcination time: 6 h, and φ = 1. Figure S20: Representative SEM images illustrating the impact of the precursor solution’s pH on the surface morphology of the synthesized NiMoO_4_ materials. Calcination temperature = 500 °C, calcination time = 6 h, and φ = 1. Figure S21: TEM images of NiMoO_4_ samples produced at different precursor solution’s pH (1, 6, 9). Calcination temperature: 500 °C, calcination time: 6 h, and φ = 1. Figure S22: Tauc plots derived from the UV-Vis absorption spectra of NiMoO_4_ samples produced at different precursor solution’s pH (1, 3, 4.57, 5, 6, 7, 9). Calcination temperature: 500 °C, calcination time: 6 h, and φ = 1. Figure S23: (a) XRD patterns and (b) FTIR spectra of NiMoO_4_ samples produced at different fuel-to-oxidant ratios (φ); (α) α-NiMoO_4_, (β) β-NiMoO_4_, (♦) MoO_3_ and (Δ) NiO. Calcination temperature: 500 °C, calcination time: 6 h, and pH = 4.57. Figure S24: Nitrogen adsorption–desorption isotherms of NiMoO_4_ samples produced at different fuel-to-oxidant ratios (φ: 0, 1/3, 1/2, 1, 2, 3). Calcination temperature: 500 °C, calcination time: 6 h, and pH = 4.57. Figure S25: Representative SEM images illustrating the impact of the fuel-to-oxidant ratio (φ: 0, 1/3, 1/2, 1, 2, 3) on the surface morphology of the synthesized NiMoO_4_ materials. Calcination temperature: 500 °C, calcination time: 6 h, and pH = 4.57. Figure S26: TEM images and SAED patterns of NiMoO_4_ samples produced at different fuel-to-oxidant ratios (φ: 0, 1/3, 1/2, 1, 2, 3). Calcination temperature: 500 °C, calcination time: 6 h, and pH = 4.57. Figure S27: Tauc plots obtained from UV-Vis absorption spectra of NiMoO_4_ samples produced at different fuel-to-oxidant ratios (φ: 0, 1/3, 1/2, 1, 2, 3). Calcination temperature: 500 °C, calcination time: 6 h, and pH = 4.57.

## Sample Availability

Samples of the compounds are not available from the authors.
