# Supplementary material for: Part I: NiMoO4 Nanostructures Synthesized by the Solution Combustion Method: A Parametric Study on the Influence of Synthesis Parameters on the Materials’ Physicochemical, Structural, and Morphological Properties
Source: Molecules. 2022 Jan 25;27(3):776. doi: 10.3390/molecules27030776 (PMC8839866; doi:10.3390/molecules27030776)
Supplement: Supplementary file 1 [file molecules-27-00776-s001.zip › molecules-1525695-supplementary.pdf]

Supplementary Information

Part I: $NiMoO_4$ Nanostructures Synthesized by the Solution Combustion Method: A Parametric Study on the Influence of Synthesis Parameters on the Materials' Physicochemical, Structural, and Morphological properties

Mahmoud Bassam Rammal [1*], Sasha Omanovic [1]

[1]Department of Chemical Engineering, McGill University, 3610 University Street, Montreal, Quebec, H3A 0C5, Canada

*Correspondence: mahmoud.rammal@mail.mcgill.ca

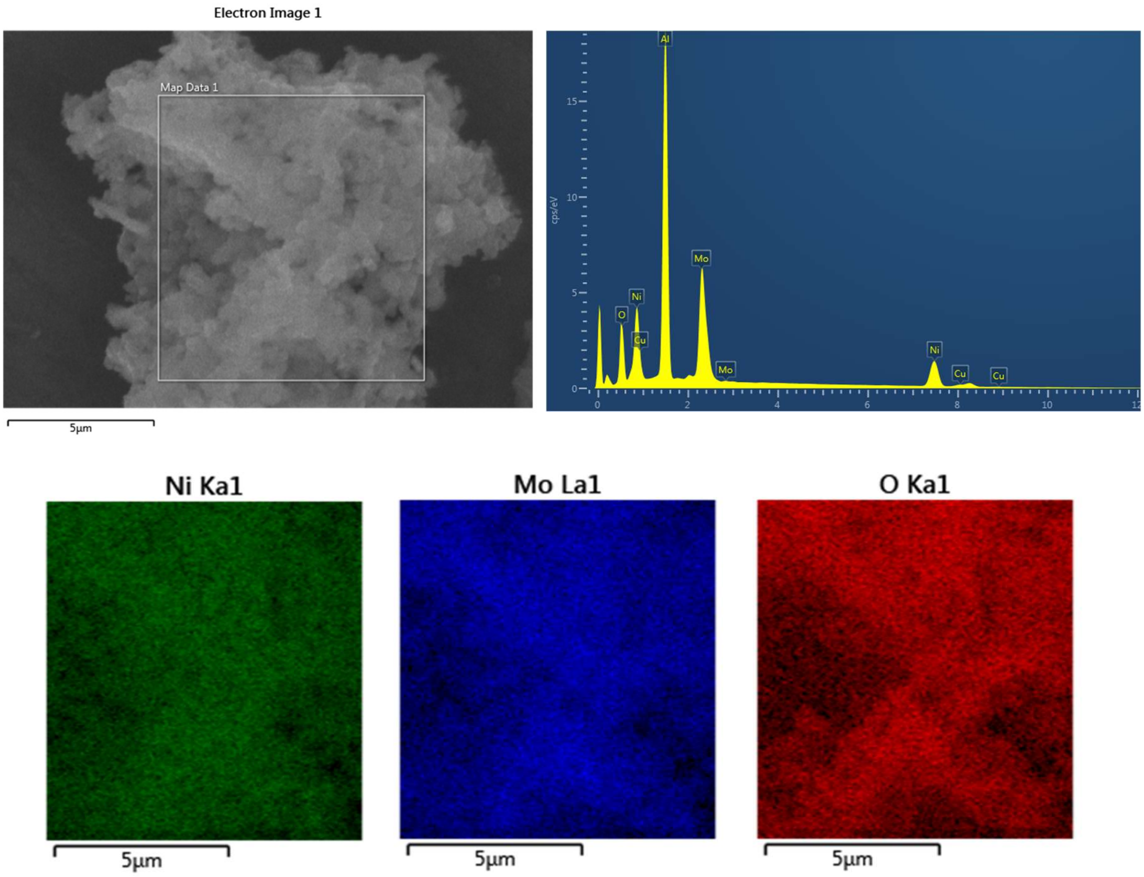

Figure S1. SEM image showing the $NiMoO_4$ sample calcined at 300 ^oC and its EDX analysis in the mapping mode, depicting the distribution of Ni, Mo, and O in the sample; the presence of Al and Cu in the EDX spectra is due to the deposition of the sample directly on the SEM aluminum stud, before imaging. No carbon was detected in the sample. Calcination time: 6 h, pH = 4.57, and $\varphi = 1$.

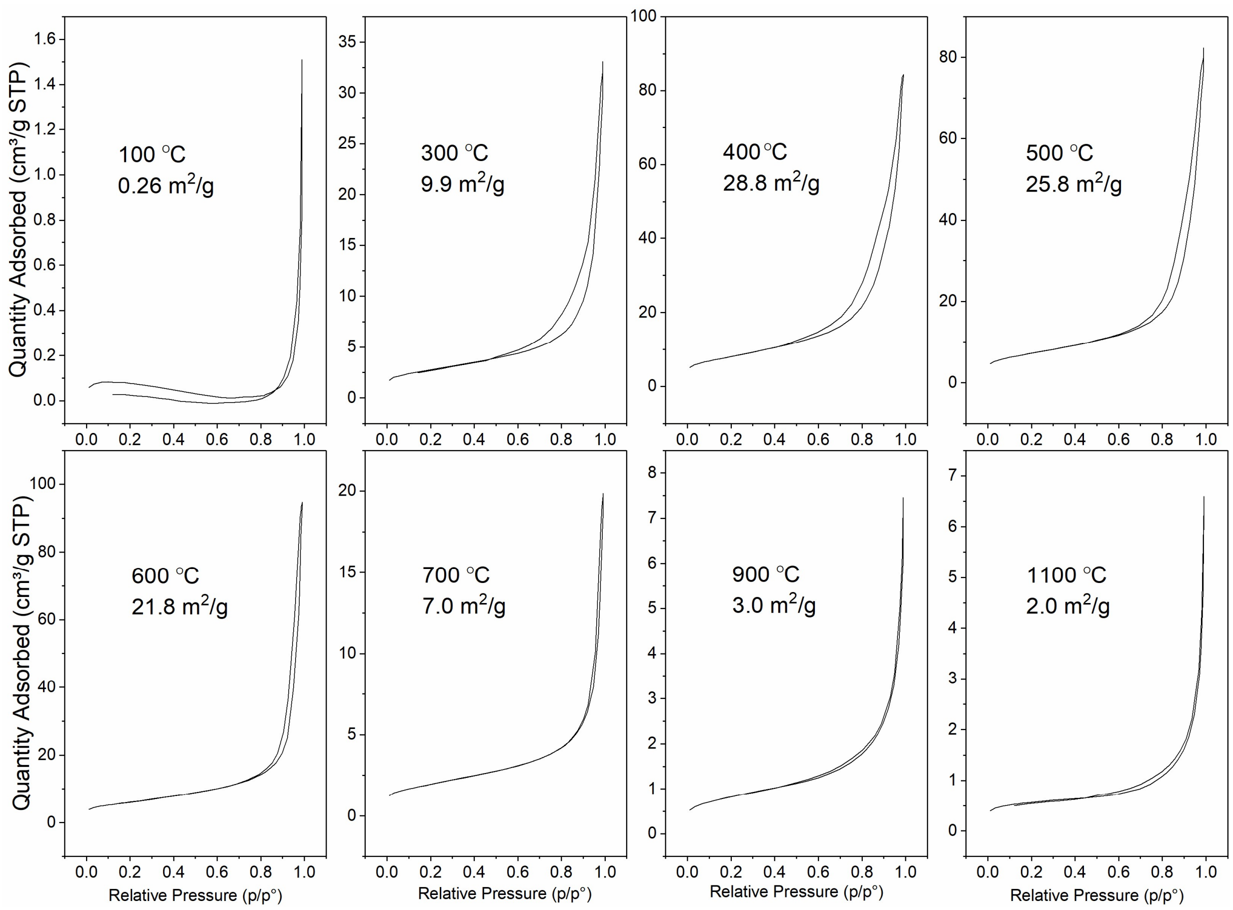

Figure S2. Nitrogen adsorption-desorption isotherms of $NiMoO_4$ samples produced at different calcination temperatures. Calcination time: 6 h, pH = 4.57, and φ = 1.

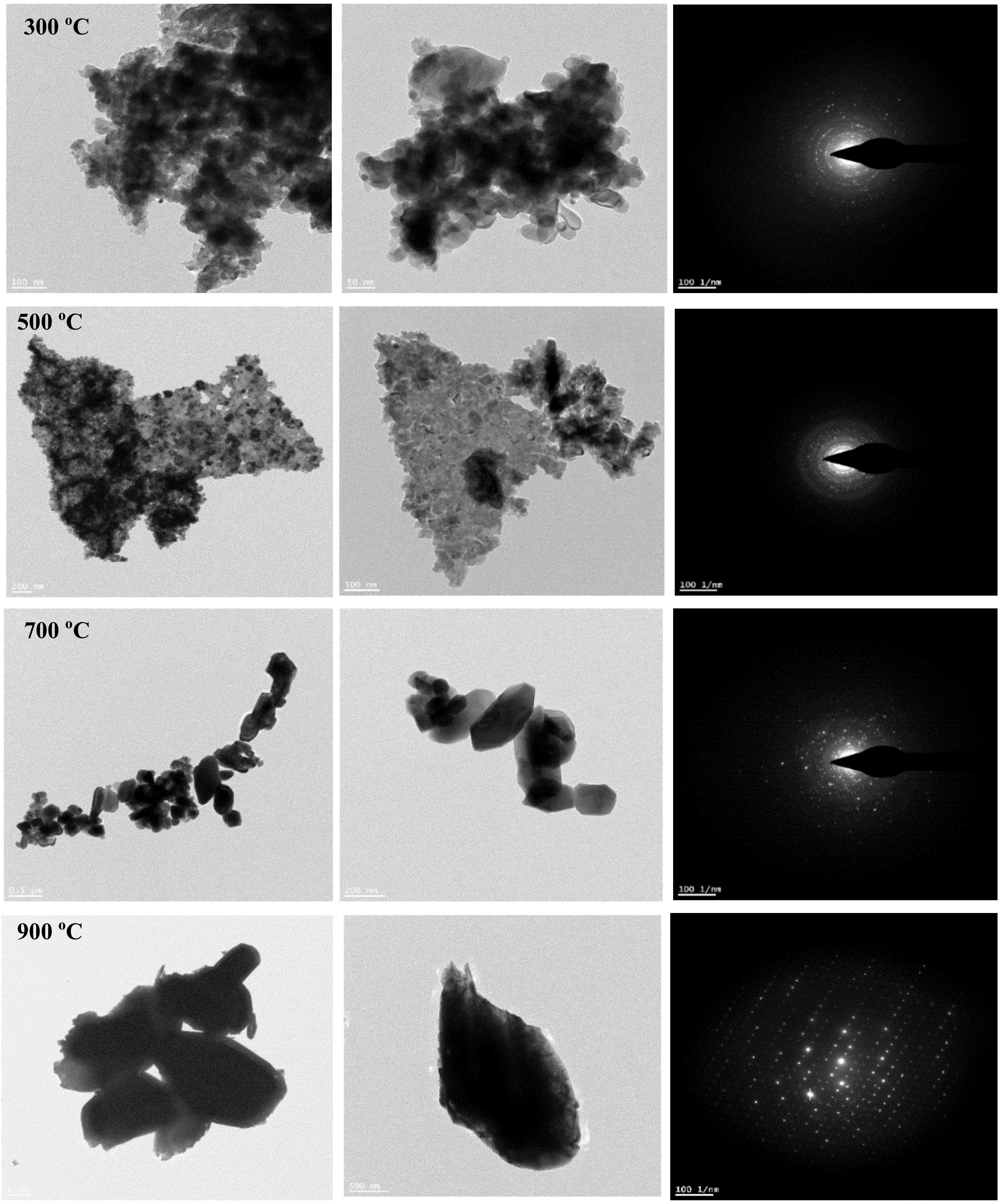

Figure S3. TEM images of $NiMoO_4$ samples produced at different calcination temperatures. Calcination time: 6 h, pH = 4.57, and $\varphi = 1$.

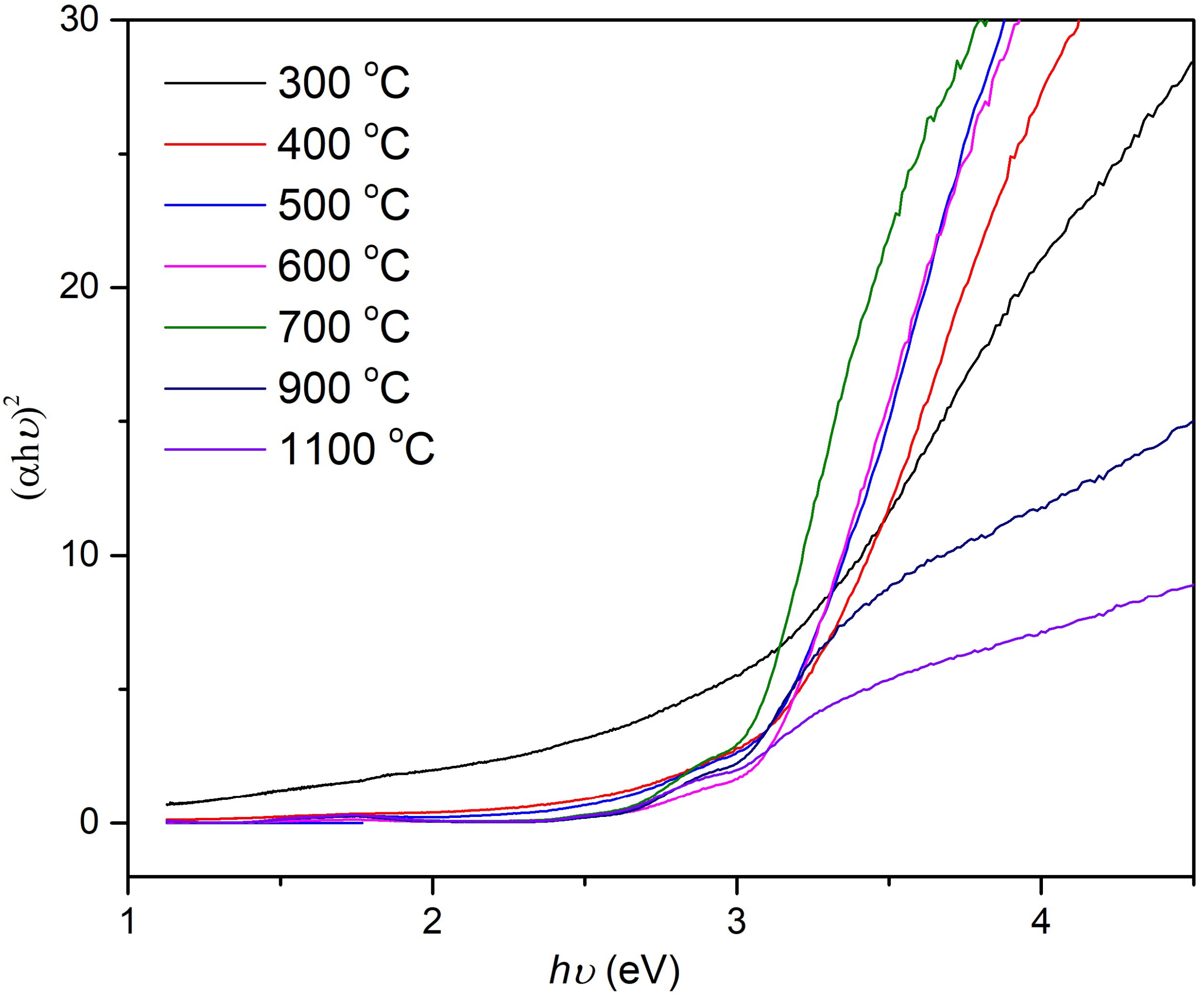

Figure S4. Tauc plots derived from the UV-Vis absorption spectra of $NiMoO_4$ samples produced at different calcination temperatures. Calcination time: 6 h, pH = 4.57, and φ = 1.

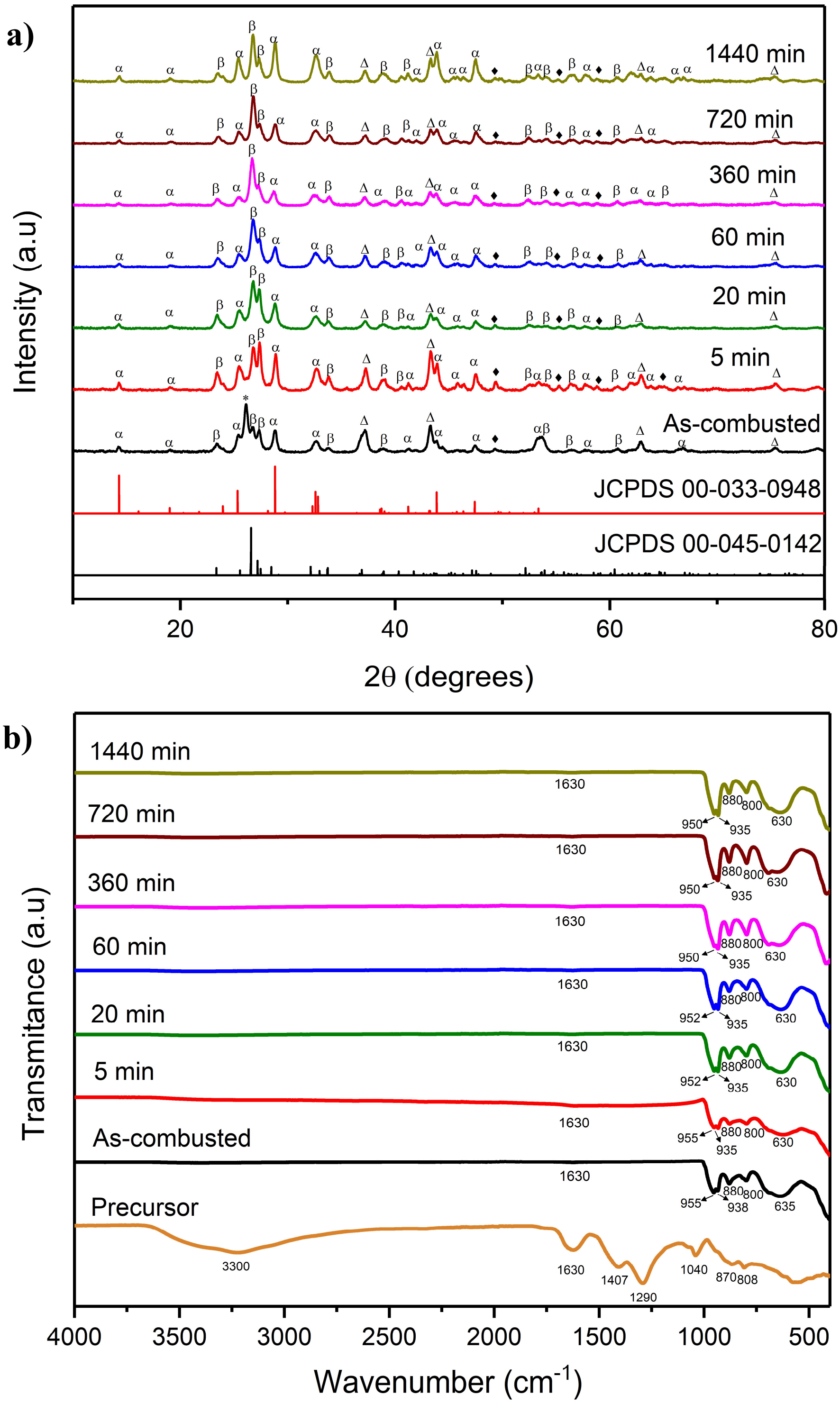

Figure S5. (a) XRD patterns, and (b) FTIR spectra of $NiMoO_4$ samples produced during different calcination times; (α) α-$NiMoO_4$, (β) β-$NiMoO_4$, (*): MoO_2, (♦) MoO_3, and (Δ) NiO. Calcination temperature: 500 ºC, pH = 4.57, and φ = 1.

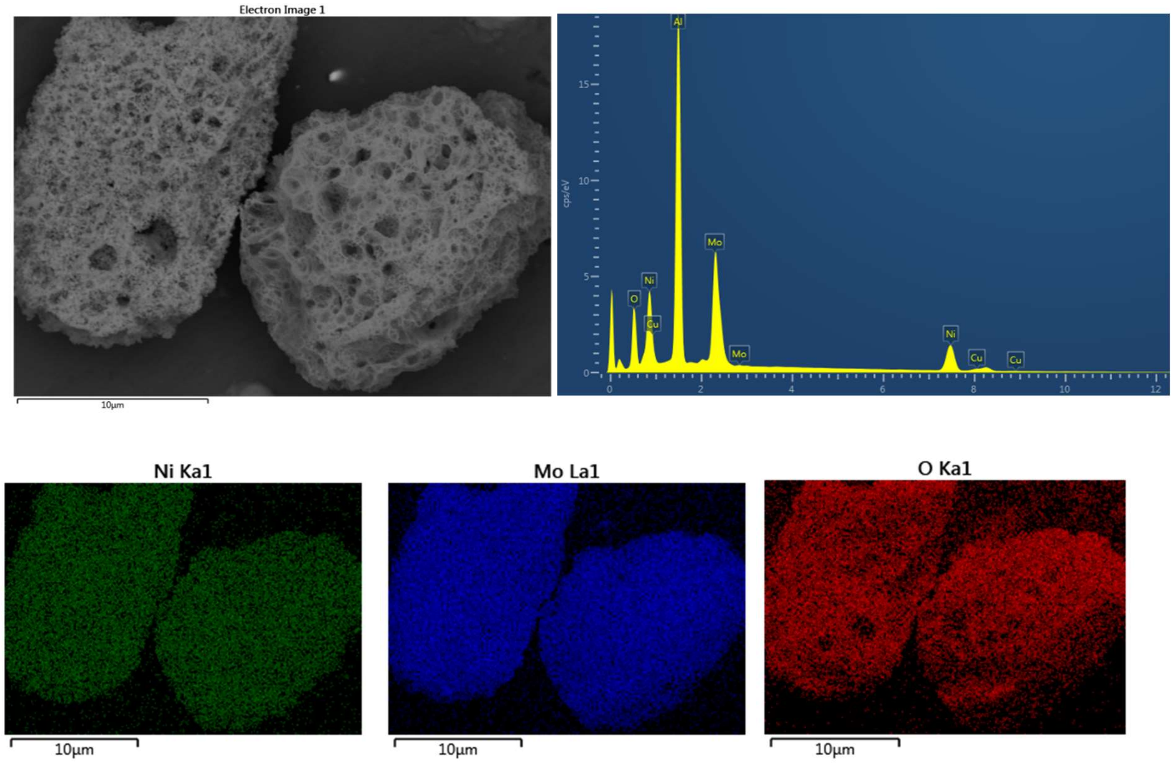

Figure S6. SEM image showing the as-combusted $NiMoO_4$ sample and the EDX analysis in the mapping mode depicting the distribution of Ni, Mo, and O in the sample; the presence of Al and Cu in the EDX spectra is due to the deposition of the sample directly on the SEM aluminum stud, before imaging. No carbon was detected in the sample. Calcination temperature: 500 ^{o}C, pH = 4.57, and $\varphi = 1$.

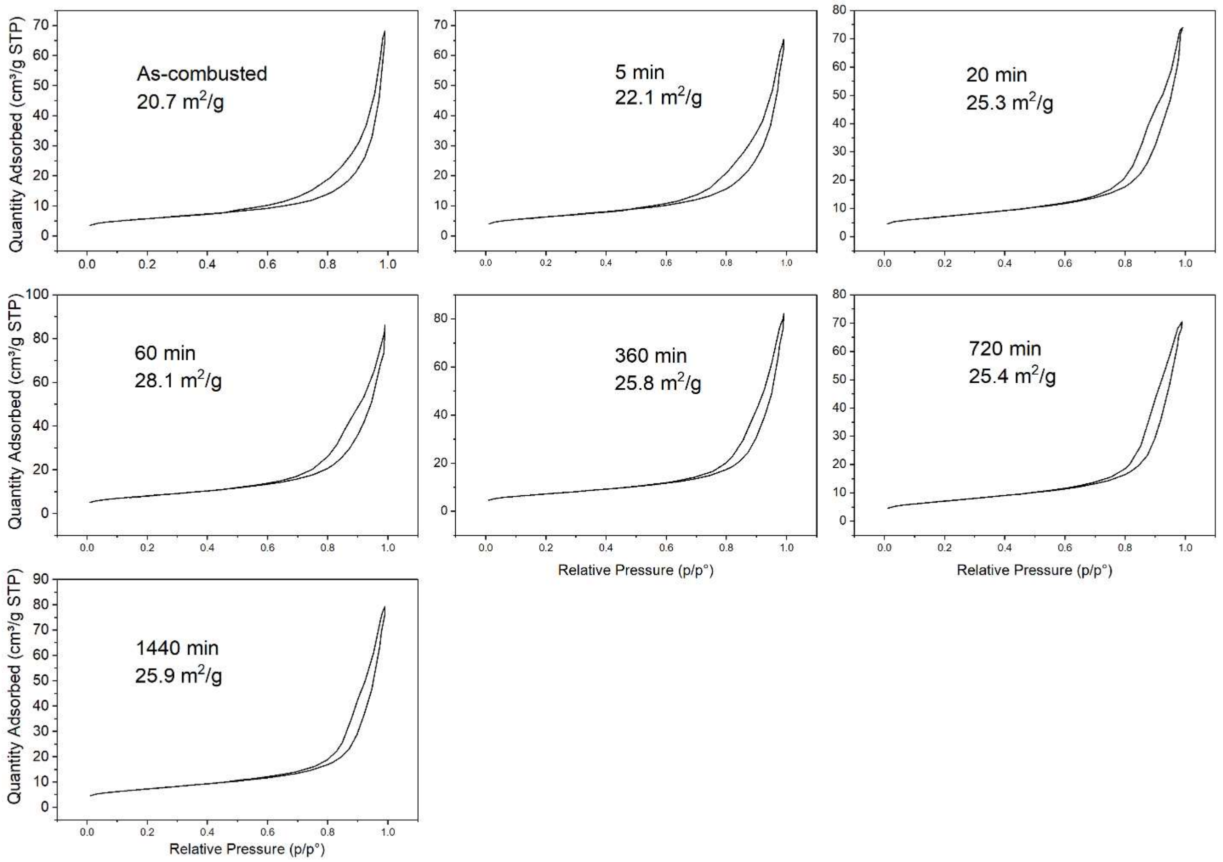

Figure S7. Nitrogen adsorption-desorption isotherm of $NiMoO_4$ samples produced during different calcination periods; the plot labeled "as-combusted" refers to the sample removed immediately from the furnace after the combustion reaction and the other isotherms denote samples subjected to different calcination periods (5 min, 60 min, 360 min, 720 min, and 1440 min). Calcination temperature: 500 °C, pH = 4.57, and φ = 1.

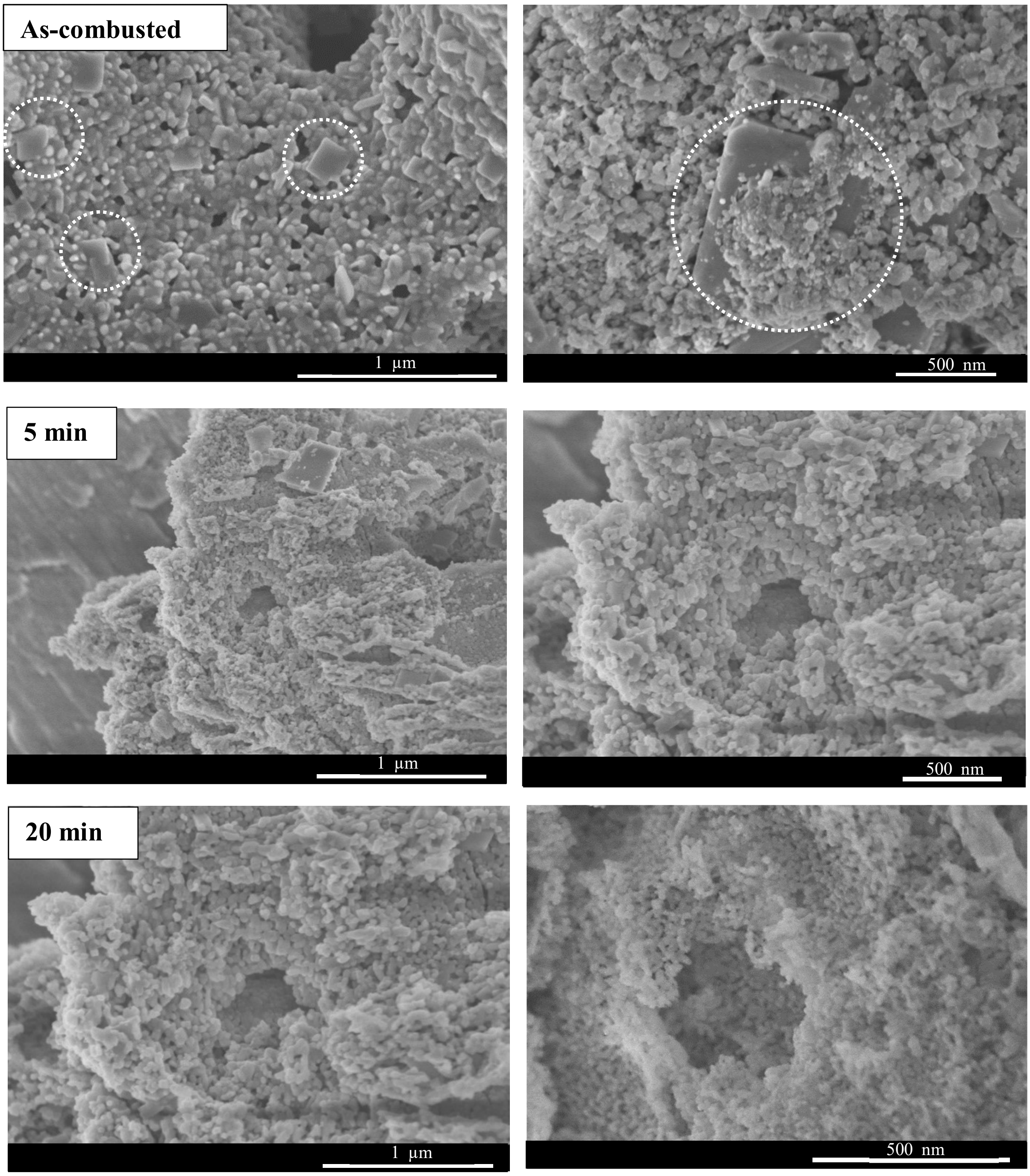
As-combusted
1 μm
500 nm
5 min
1 μm
500 nm
20 min
1 μm
500 nm

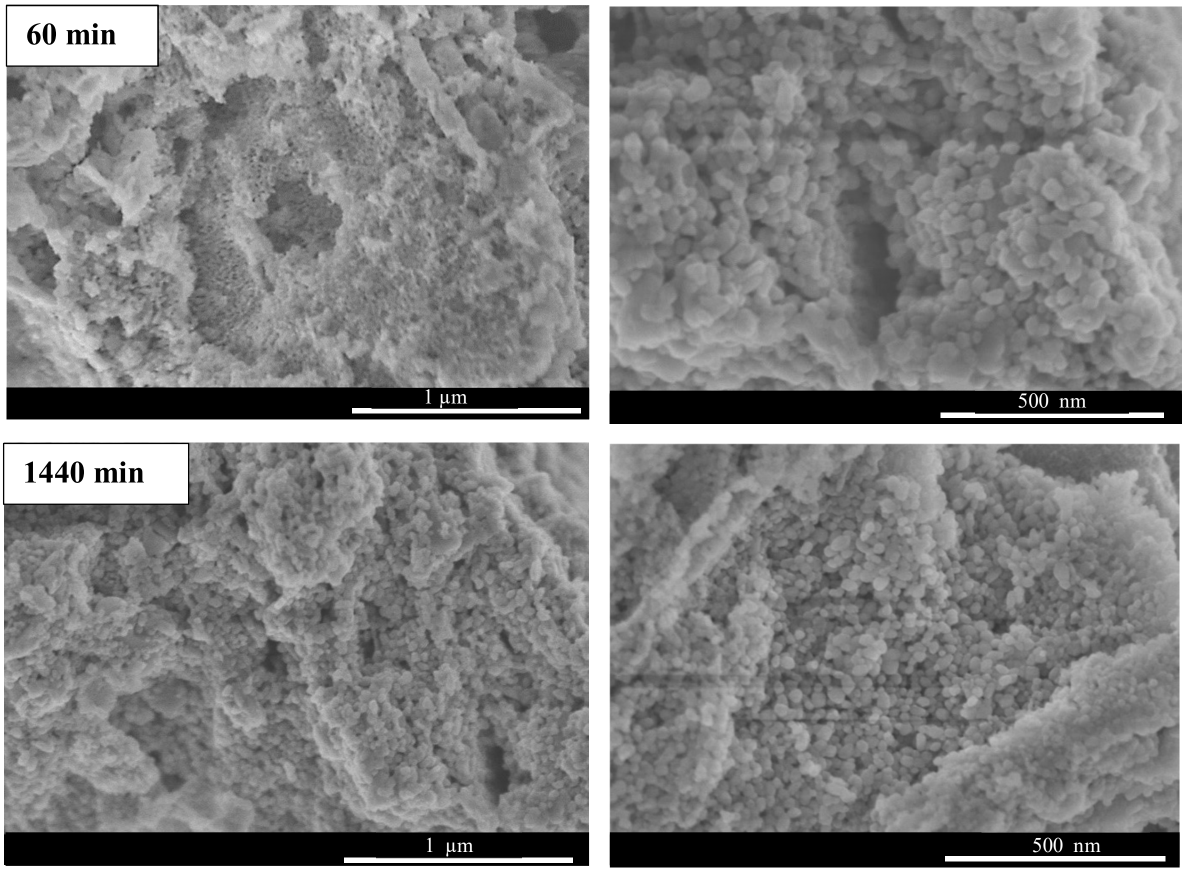

Figure S8. Representative SEM images illustrating the effect of calcination time on the surface morphology of $NiMoO_4$ samples. Calcination temperature: 500 °C, pH = 4.57, and φ = 1.

As-combusted

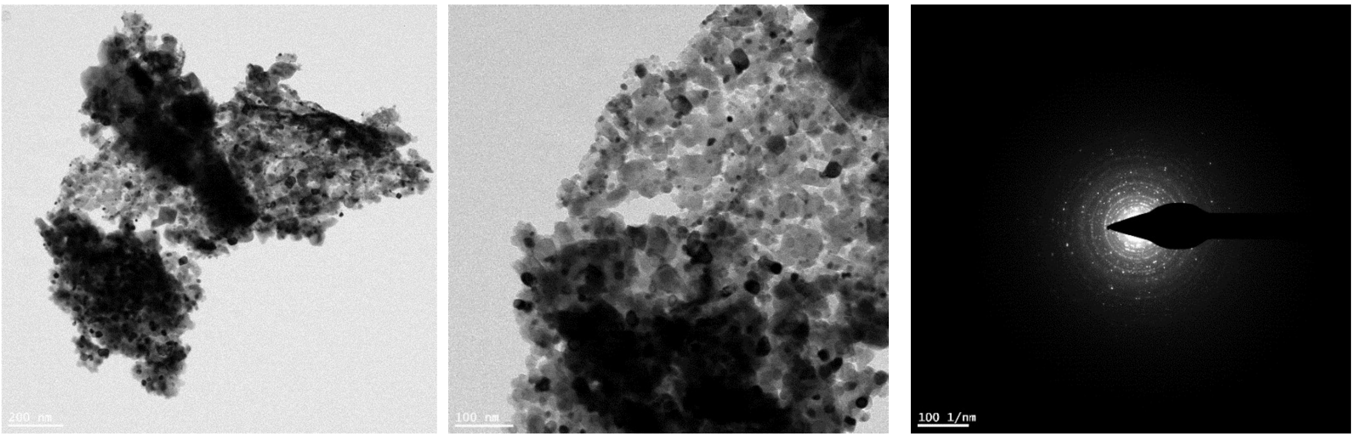

1440 min

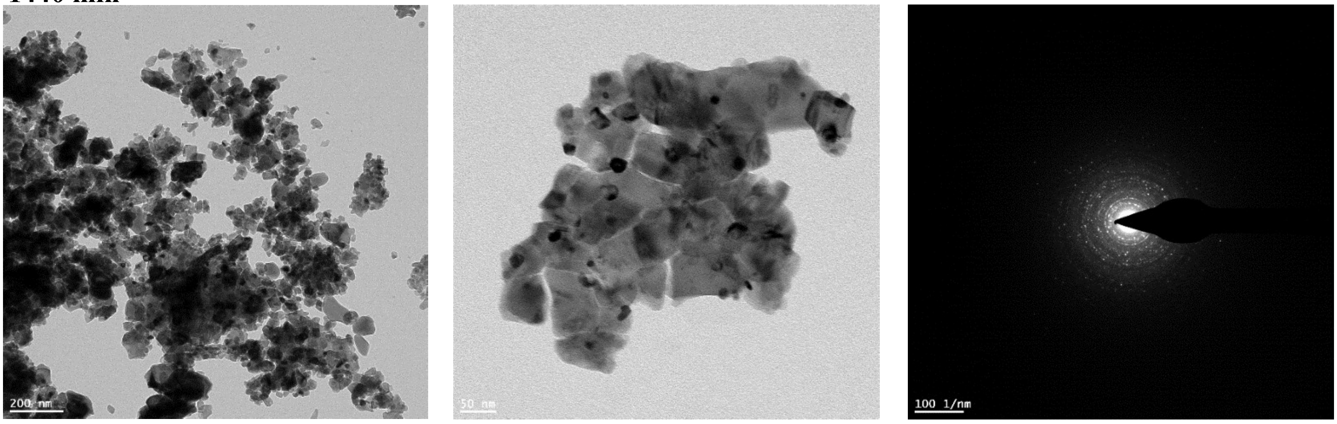

Figure S9. TEM images of the as-combusted $NiMoO_4$ sample and that one calcined for 24 h. Calcination temperature: 500 ^{o}C, pH = 4.57, and $\varphi = 1$.

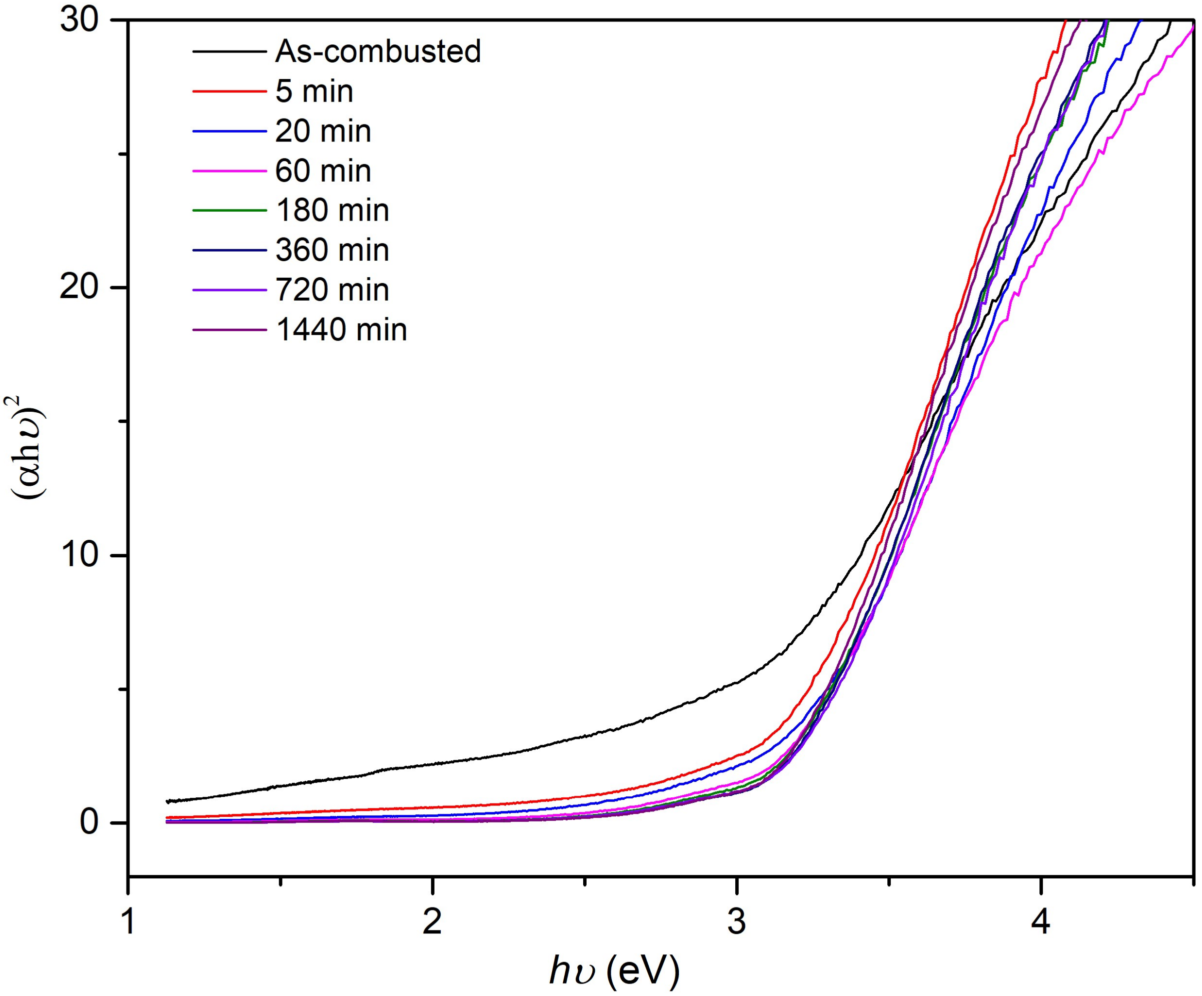

Figure S10. Tauc plots derived from the UV-Vis absorption spectra for the $NiMoO_4$ samples produced during different calcination periods. Calcination temperature: 500 ºC, pH = 4.57, and φ = 1.

The following approach was used to obtain T_{ad}, by initially determining the total heat (Q, J/mol) released in the reaction according to the following:

$$Q = \Delta H_f^0 = \Delta H_f^0(products) - \Delta H_f^0(reactants) \quad \text{(S1)}$$

ΔH_r^0 and ΔH_p^0 (J/mol) are the enthalpies of formation of the reactants and products. The above equation assumes adiabatic conditions (the heat released during the reaction is used to heat the reaction product to T_{ad} without accounting for thermal losses).

According to thermodynamic principles, the amount of heat needed to raise the products' temperature to T_{ad} is:

$$Q = \Delta H_f^0 = \int_{298}^{T_{ad}} C_P \, dT \quad \text{(S2)}$$

where ΔC_P represents the heat capacity of the products at a constant pressure (J/mol·K) and could be assumed, in the first approximation, to be independent of the temperature. This would allow a rough estimation of T_{ad} using Eq. (S1) and (S2), as shown below:

$$T_{ad} = T_o + \frac{\Delta H_r^0 - \Delta H_p^0}{C_P} \quad \text{(S3)}$$

where T_o = 298 K for the reference state

It is to be noted that T_{ad} is not necessarily equal to the actual combustion temperature which is, usually, considerably lower due to several factors such as heat loss, incomplete combustion, irradiated losses, and heating contribution [1]. Assuming N_2, H_2O, CO_2, and NH_3 are typical combustion by-products, the redox reaction that takes place during the production of $NiMoO_4$ can be approximated as:

$$7Ni(NO_3)_2\times 6H_2O + (NH_4)_6Mo_7O_{24}\times 4H_2O + \left(\frac{35}{31}\varphi\right)C_{14}H_{24}O_9 + \frac{35}{2}(\varphi - 1)O_2 \rightarrow 7NiMoO_4 + \left(\frac{420}{31}\varphi + 49\right)H_2O + 7N_2 + \left(\frac{490}{31}\varphi\right)CO_2 + 6NH_3 \quad \text{(S4)}$$

where φ stand for the equivalence ratio (also known as the stoichiometric fuel-to-oxidant ratio), which is determined using the following equation:

$$\varphi = \frac{\sum(\text{Coefficient of oxidizing elements}) \text{ x } (\text{Valency})}{-\sum(\text{Coefficient of reducing elements}) \text{ x } (\text{Valency})} \quad \text{(S5)}$$

In this method, metal cations (M^{v+}), carbon, and hydrogen are considered as reducing elements with the corresponding valence for metal (v), carbon (+4), and hydrogen (+1), while oxygen (-2) is the only oxidizing element, and nitrogen is neutral. A mixture is considered stoichiometric when $\varphi = 1$, fuel-lean when $\varphi < 1$, and fuel-rich when $\varphi > 1$.

The simplified reaction shown in Eq. (S4) does not intend to convey the complexity of the redox mixture, but rather present a stoichiometric operating condition that can be used as a reference, especially when comparing the effect of fuel-rich/fuel-lean mixtures on the synthesis, as seen in Section 3.5 in the main text.

To determine the adiabatic temperature using Equation (S3), thermodynamic data for the reaction constituents are presented in Table S1. The enthalpy of formation (heat of combustion) of agar could not be found in the literature (since it was used, to the best of our knowledge, in our current work for the first time as a combustion fuel [2]); therefore, a bomb calorimeter method was employed in this work to determine its value, which was -5500 kJ/mol. It is to be noted that the enthalpy of formation of $(NH_4)_6Mo_7O_{24}$ was not utilized in the thermodynamic calculations since it was neither possible to obtain its value from the literature nor our tests using the the bomb calorimeter yielded viable results. Also, $(NH_4)_6Mo_7O_{24}$ can be considered as a self-redox precursor which would combust without fuel, so its contribution could be disregarded [3]. It is also worth to note that in our previous publication [2], the reaction of agar and $(NH_4)_6Mo_7O_{24}$ did not yield any combustion reaction.

Table S1. Relevant thermodynamics data used in the computation of adiabatic temperature.

Compound	ΔH_f^0 (kJ/mol)	C_P(J/mol.K)
[a]$Ni(NO_3)_2$	-2211.7	464
$C_{14}H_{24}O_9$	-5500	1311.7[d]
[b]$NiMoO_4$	-1026	111
[a]H_2O	-241.8	30+0.015T
[a]N_2	0	27+0.004T
[a]CO_2	-393.5	43+0.011T
[a]O_2	0	25+0.015T
[c]NH_3	-46	35.01
NH_4NO_3	-80.8	
HNO_3	-206.3	

a: [1], b: [4] c: [5], d: [6]

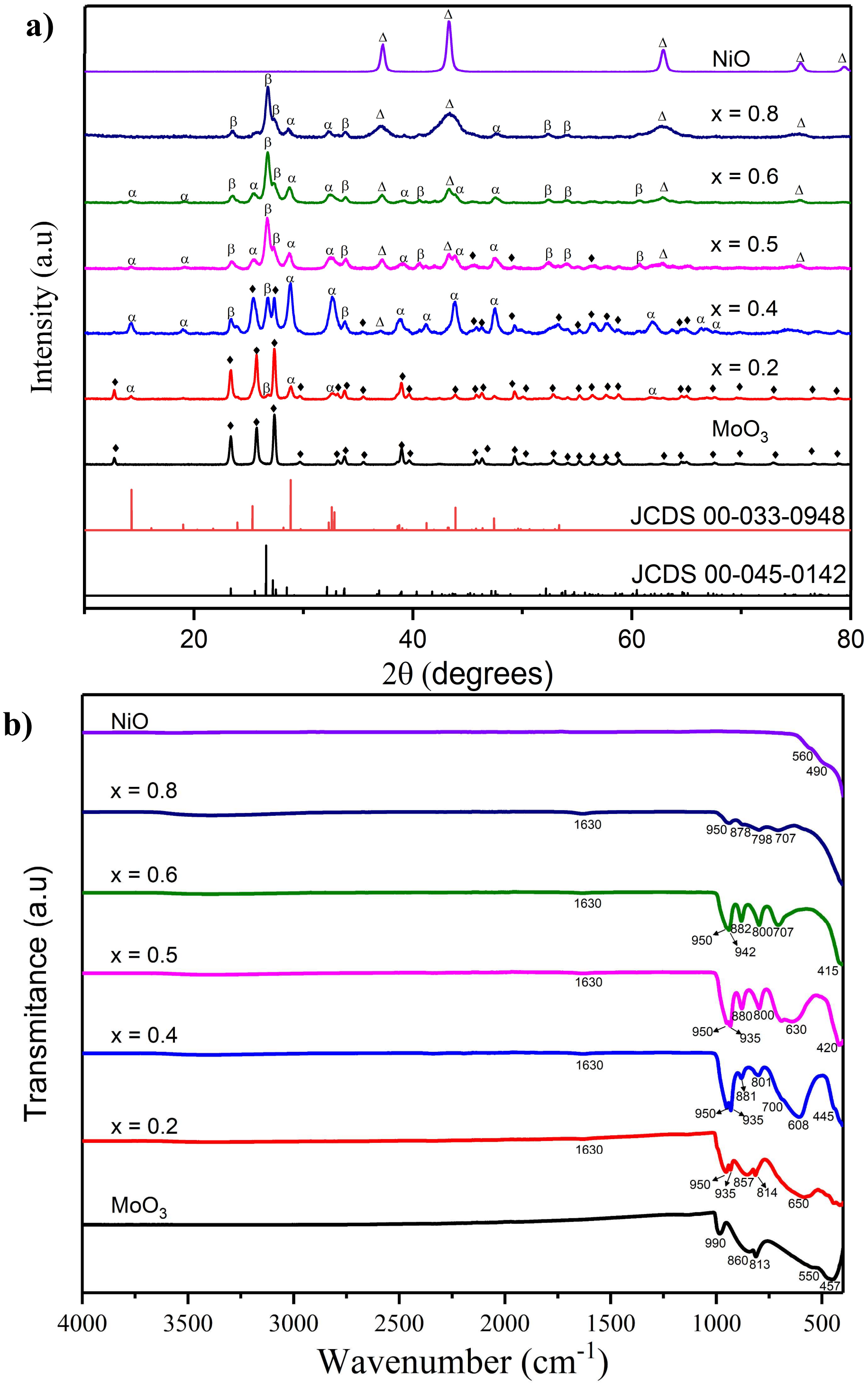

Figure S11. (a) XRD patterns, and (b) FTIR spectra of Ni_xMo_{1-x}-oxide ($0 \leq x \leq 1$) samples; (α) α-$NiMoO_4$, (β) β-$NiMoO_4$, (♦) MoO_3, and (Δ) NiO. Calcination temperature: 500 °C, calcination time: 6 h, pH = 4.57, and φ = 1.

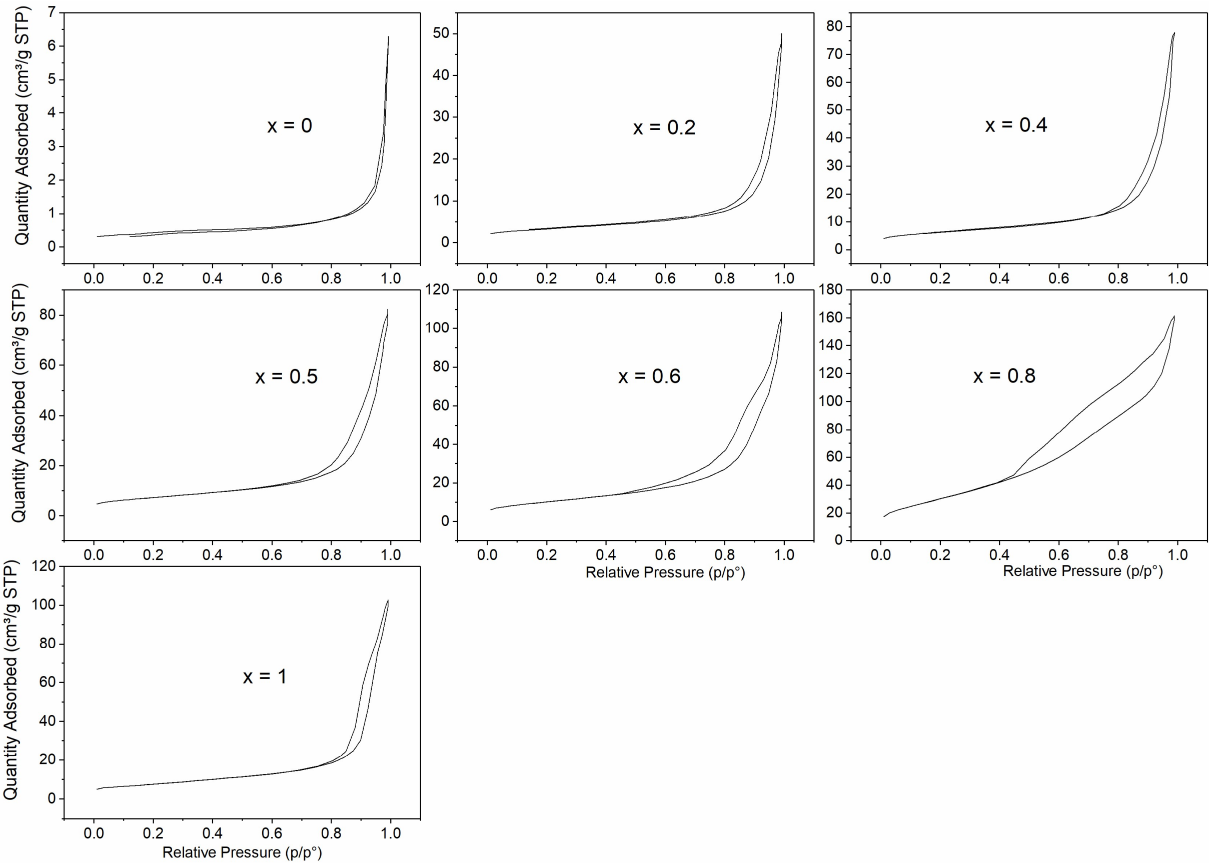

Figure S12. Nitrogen adsorption-desorption isotherms of Ni_xMo_{1-x}-oxide ($0 \leq x \leq 1$) samples (*x*: 0, 0.2, 0.4, 0.6, 0.8, 1). Calcination temperature: 500 °C, calcination time: 6 h, pH = 4.57, and φ = 1.

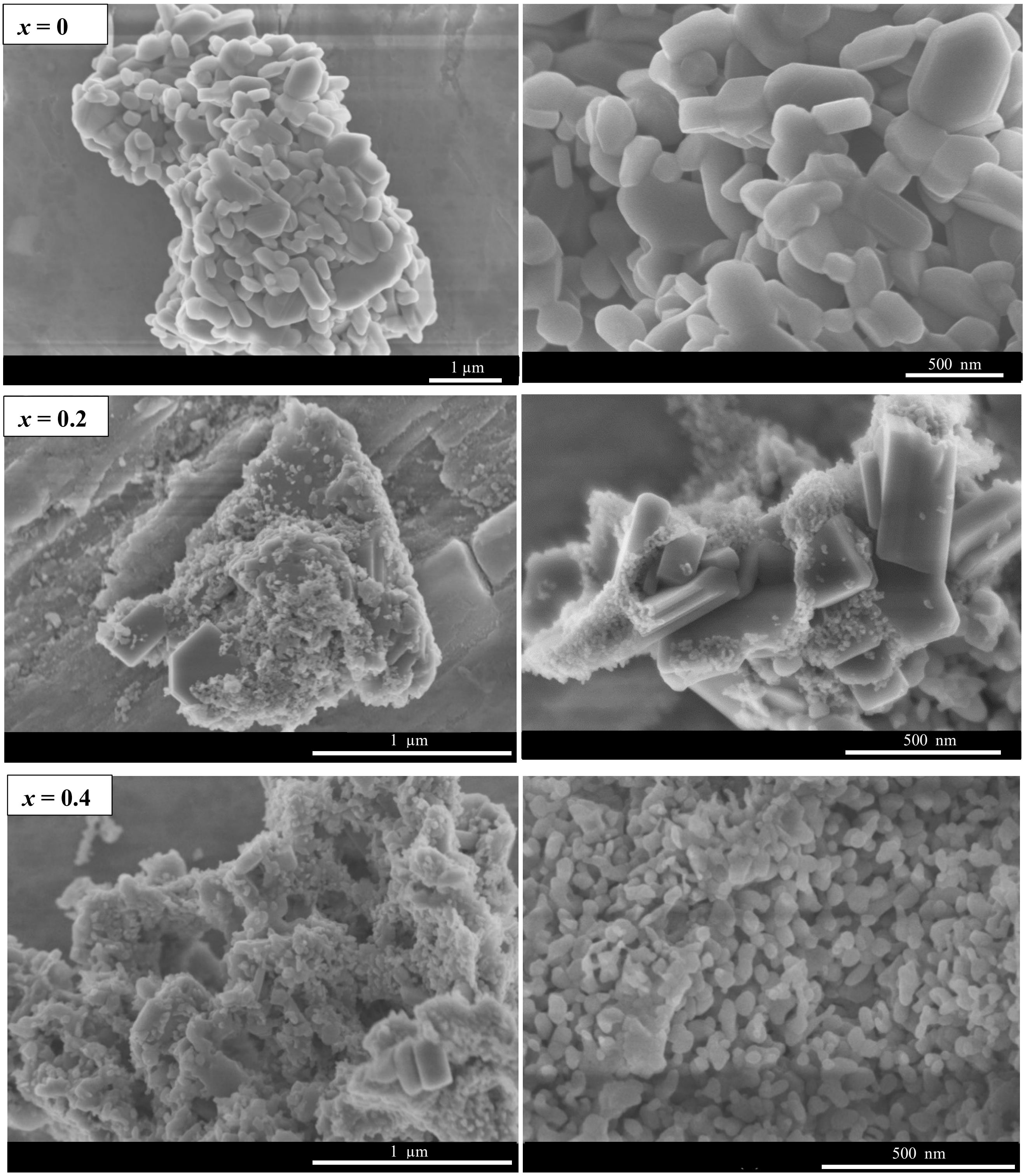

x = 0
1 μm
500 nm
x = 0.2
1 μm
500 nm
x = 0.4
1 μm
500 nm

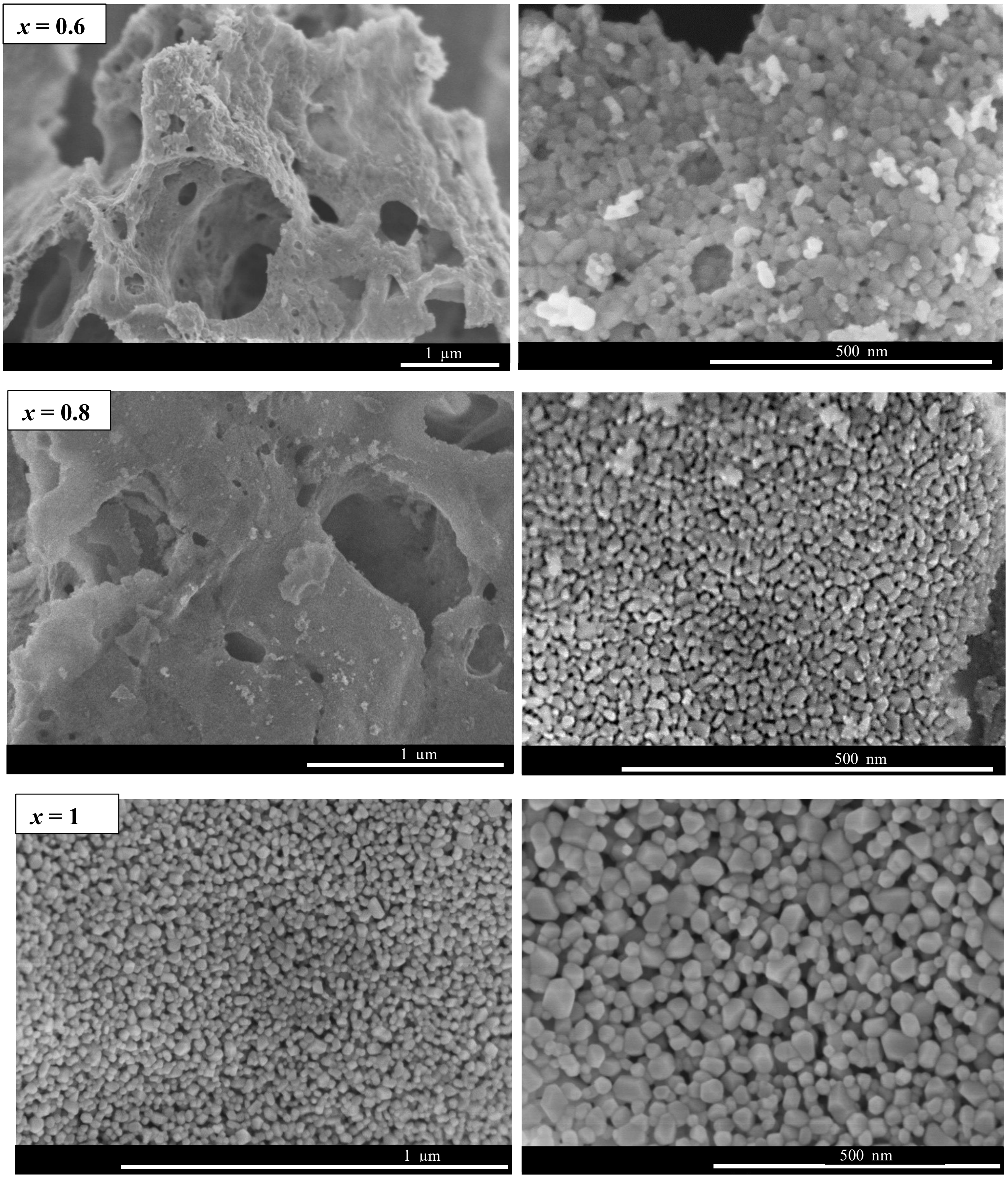

Figure S13. Representative SEM images illustrating the surface morphology of Ni_xMo_{1-x} oxide ($0 \leq x \leq 1$) samples. Calcination temperature: 500 ºC, calcination time: 6 h, pH = 4.57, and φ = 1.

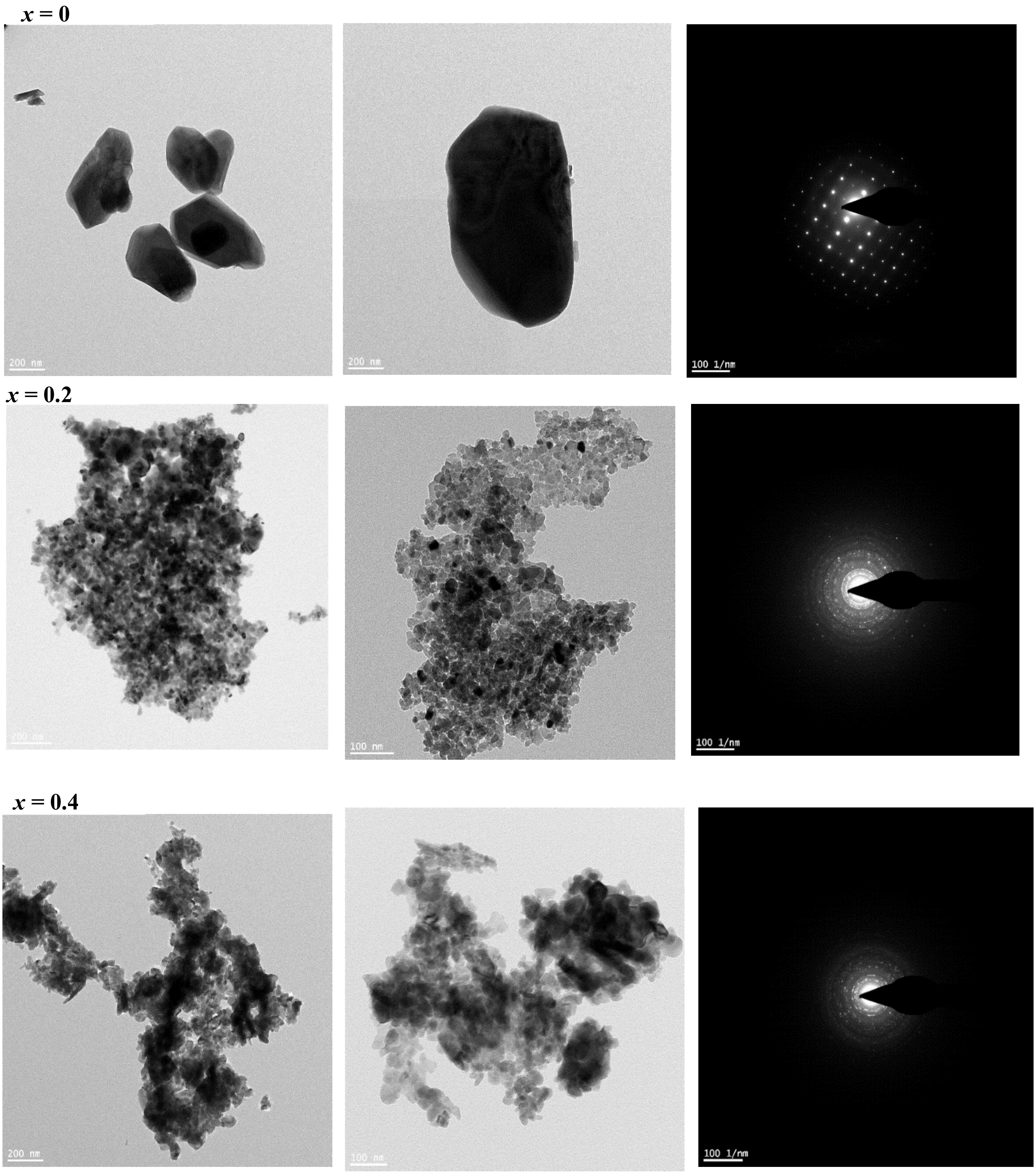
x = 0
200 nm
200 nm
100 1/nm
x = 0.2
200 nm
100 nm
100 1/nm
x = 0.4
200 nm
100 nm
100 1/nm

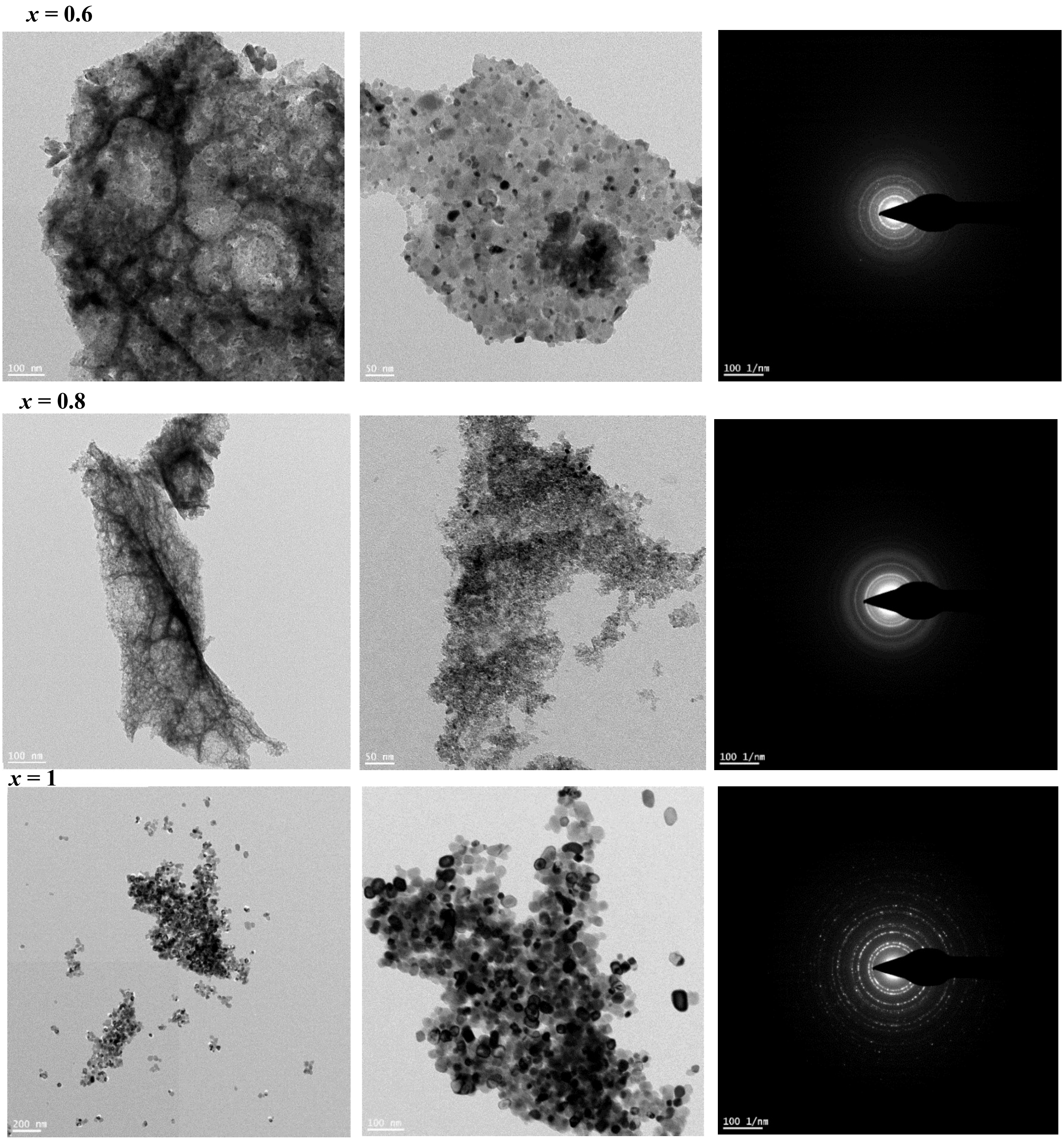

Figure S14. TEM images and SAED patterns of Ni_xMo_{1-x}-oxide ($0 \le x \le 1$) samples (x: 0, 0.2, 0.4, 0.6, 0.8, 1). Calcination temperature: 500 °C, calcination time: 6 h, pH = 4.57, and φ = 1.

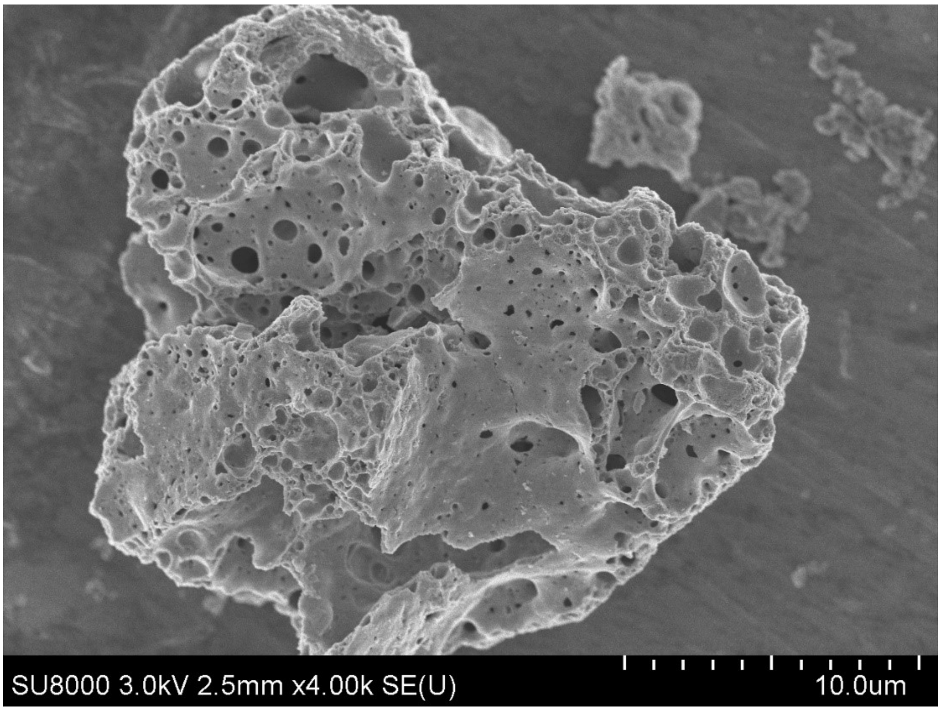

Figure S15. Low magnification SEM image of the material with Ni:Mo of 80:20 (x = 0.8). The image shows the high porosity of the material stemming from the low adiabatic temperature and vigorous gas generated during the combustion reaction. (Calcination temperature: 500 ^{o}C, calcination time: 6 h, pH = 4.57, and φ = 1).

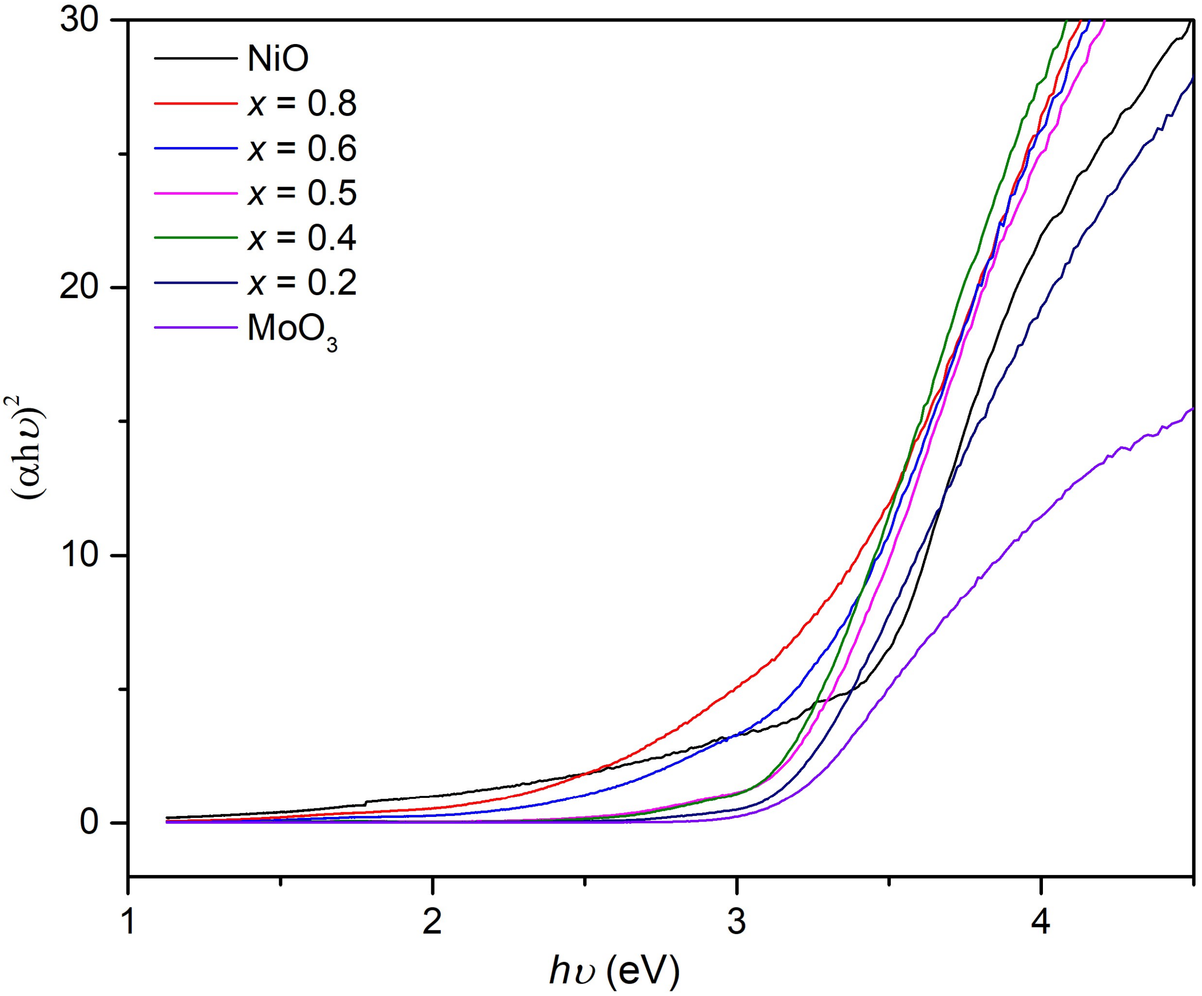

Figure S16. Tauc plots derived from the UV-Vis absorption spectra of Ni_xMo_{1-x}-oxide ($0 \leq x \leq 1$) samples (x: 0, 0.2, 0.4, 0.6, 0.8, 1). Calcination temperature: 500 °C, calcination time: 6 h, pH = 4.57, and φ =1.

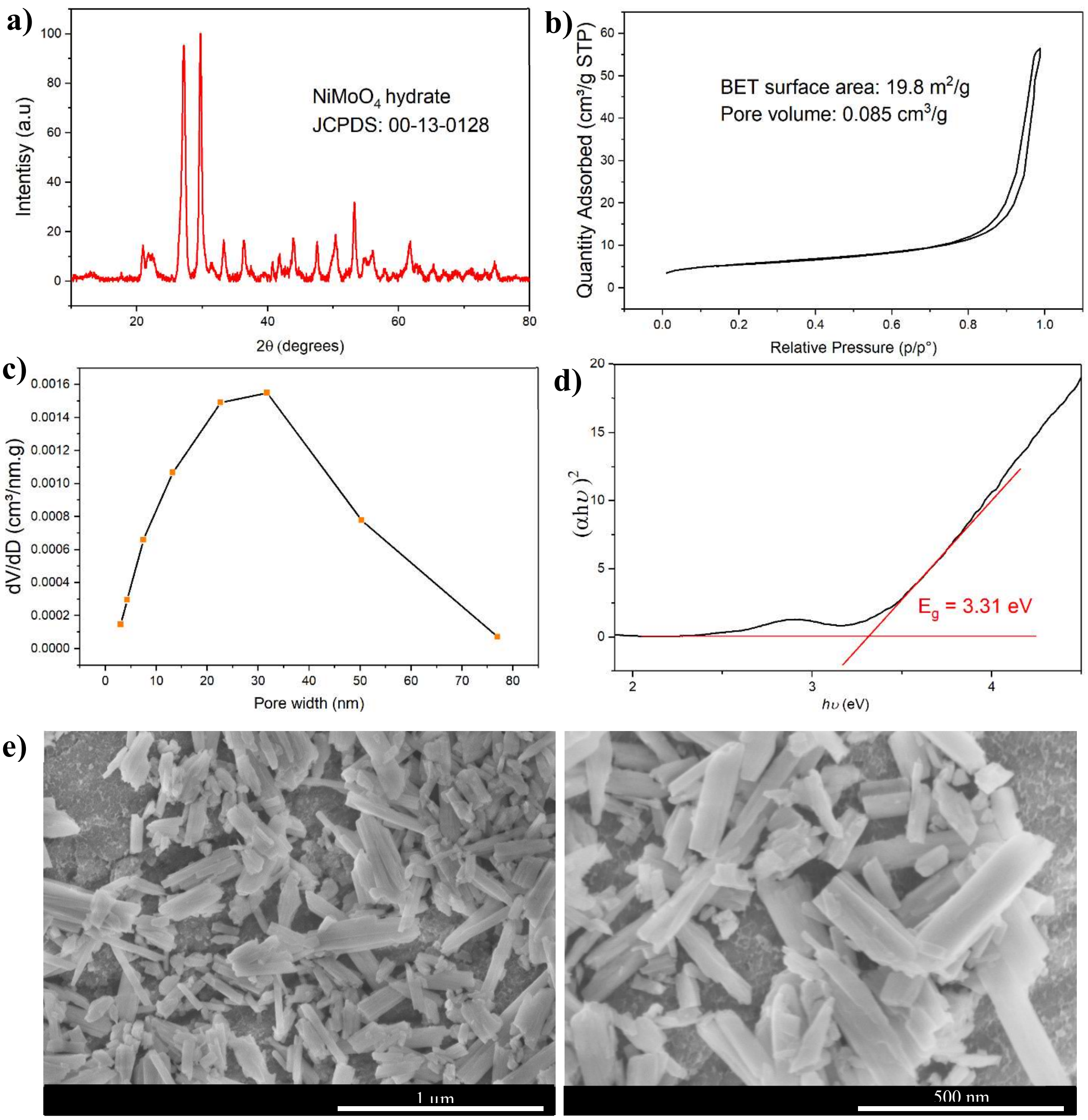

Figure S17. Characterization of commercial $NiMoO_4$, procured from Alfa Aesar; (a) XRD pattern and the corresponding indexed standard that best matches the observed spectrum, (b) Nitrogen adsorption-desorption isotherm used to determine the BET surface area, pore volume, and pore size distribution, (c) pore size distribution obtained using the Kelvin equation and BJH model, (d) Tauc plot obtained from the UV-Vis absorption spectrum to calculate the band gap, and (e) representative SEM images taken at a different magnification, showing the presence of nanorods with a diameter range of 70-120 nm, and length range of 300-450 nm.

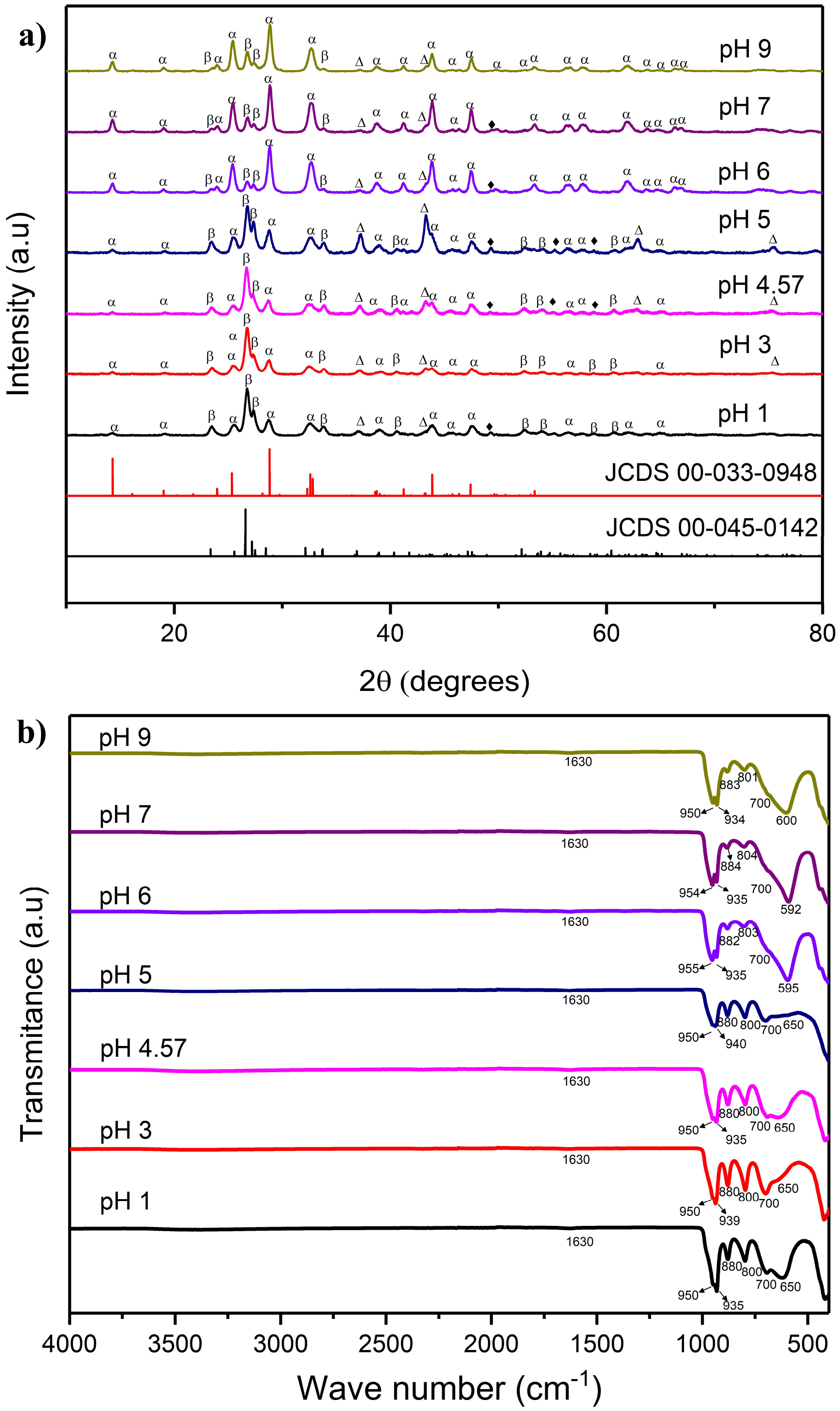

Figure S18. (a) XRD patterns, and (b) FTIR spectra of $NiMoO_4$ samples produced at different precursor solution's pH; (α) α-$NiMoO_4$, (β) β-$NiMoO_4$, (♦) MoO_3, and (Δ) NiO. Calcination temperature: 500 °C, calcination time: 6 h, and φ =1.

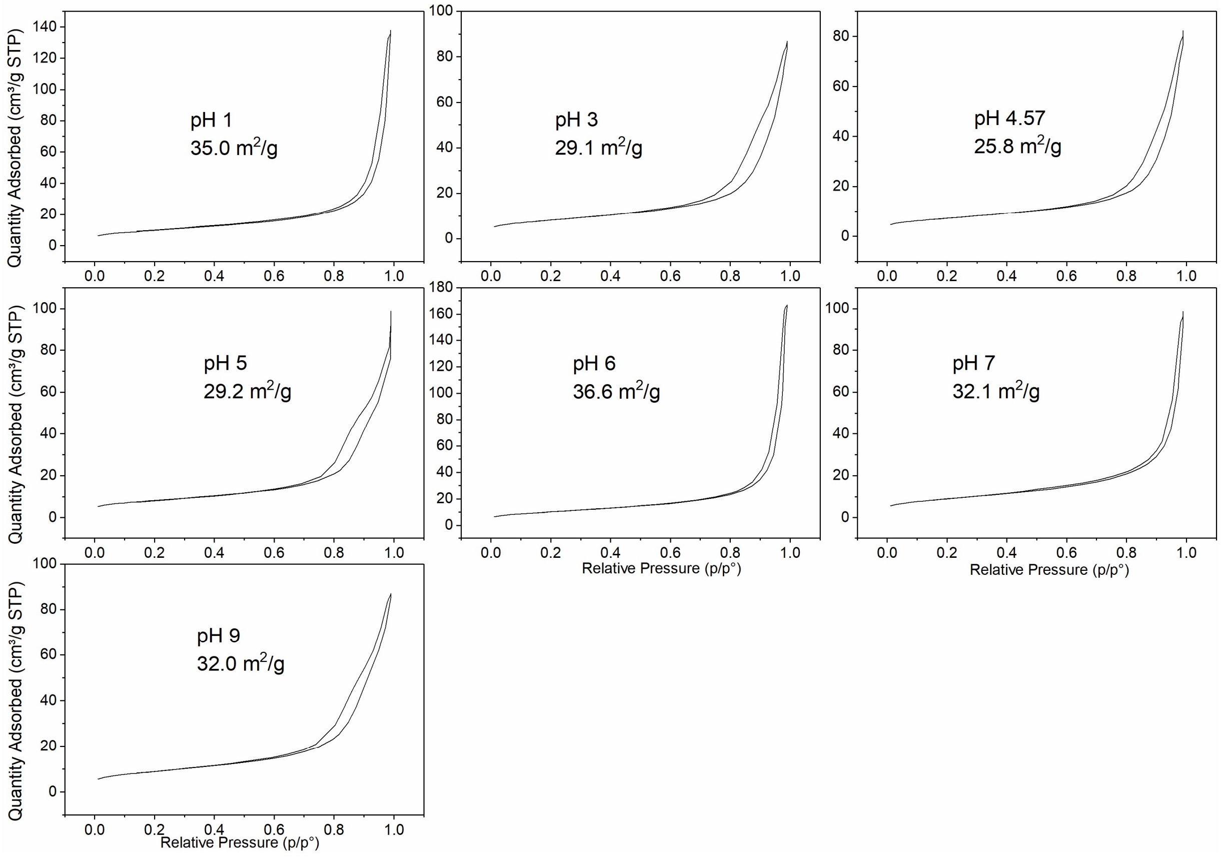

Figure S19. Nitrogen adsorption-desorption isotherms of $NiMoO_4$ samples produced at different precursor solution's pH (1, 3, 4.57, 5, 6, 7, 9). Calcination temperature: 500 °C, calcination time: 6 h, and φ =1.

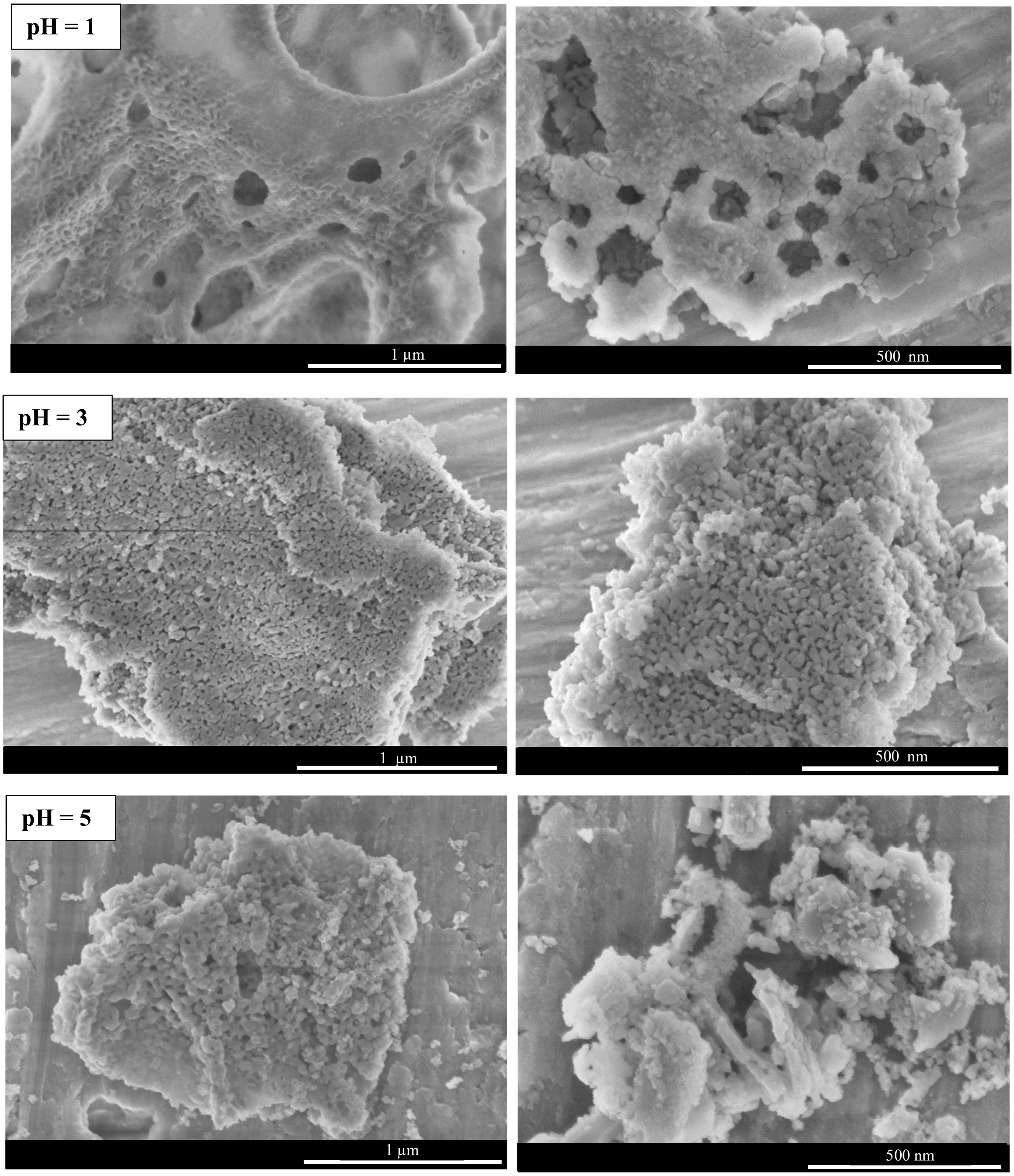
pH = 1
1 µm
500 nm
pH = 3
1 µm
500 nm
pH = 5
1 µm
500 nm

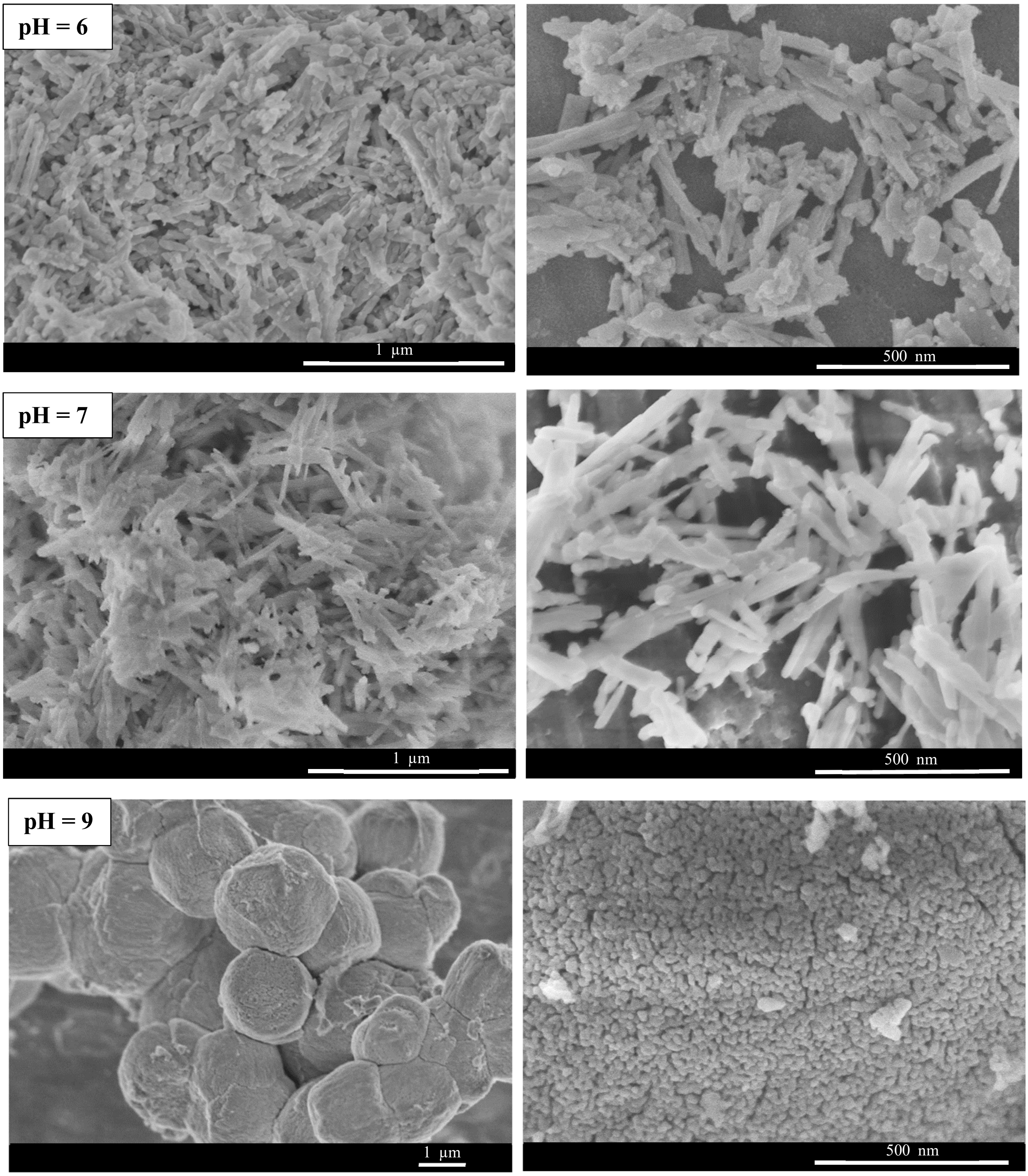

Figure S20. Representative SEM images illustrating the impact of the precursor solution's pH on the surface morphology of the synthesized $NiMoO_4$ materials. Calcination temperature = 500 ^{o}C, calcination time = 6 h, and φ = 1.

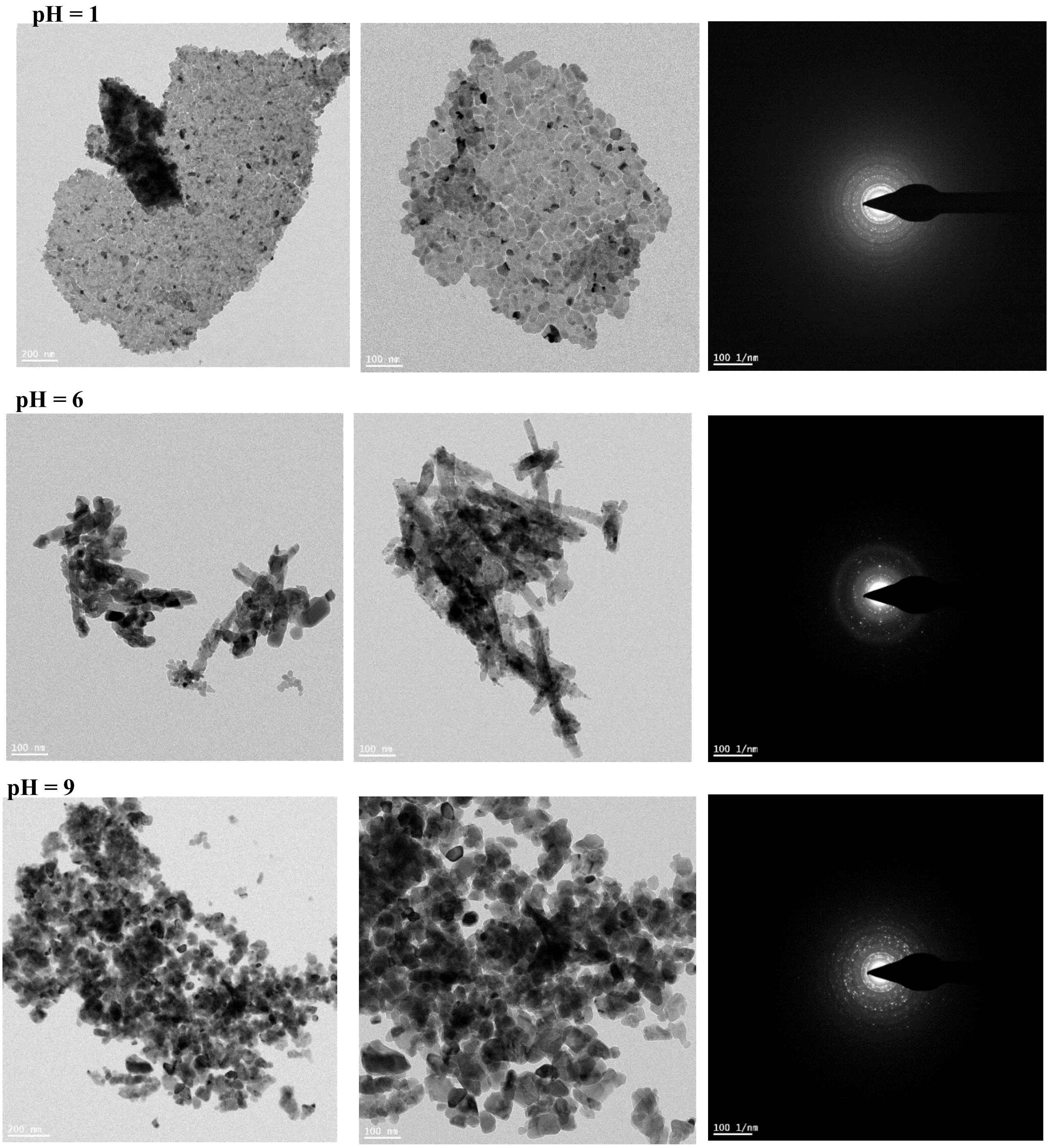

Figure S21. TEM images of $NiMoO_4$ samples produced at different precursor solution's pH (1, 6, 9). Calcination temperature: 500 °C, calcination time: 6 h, and φ = 1.

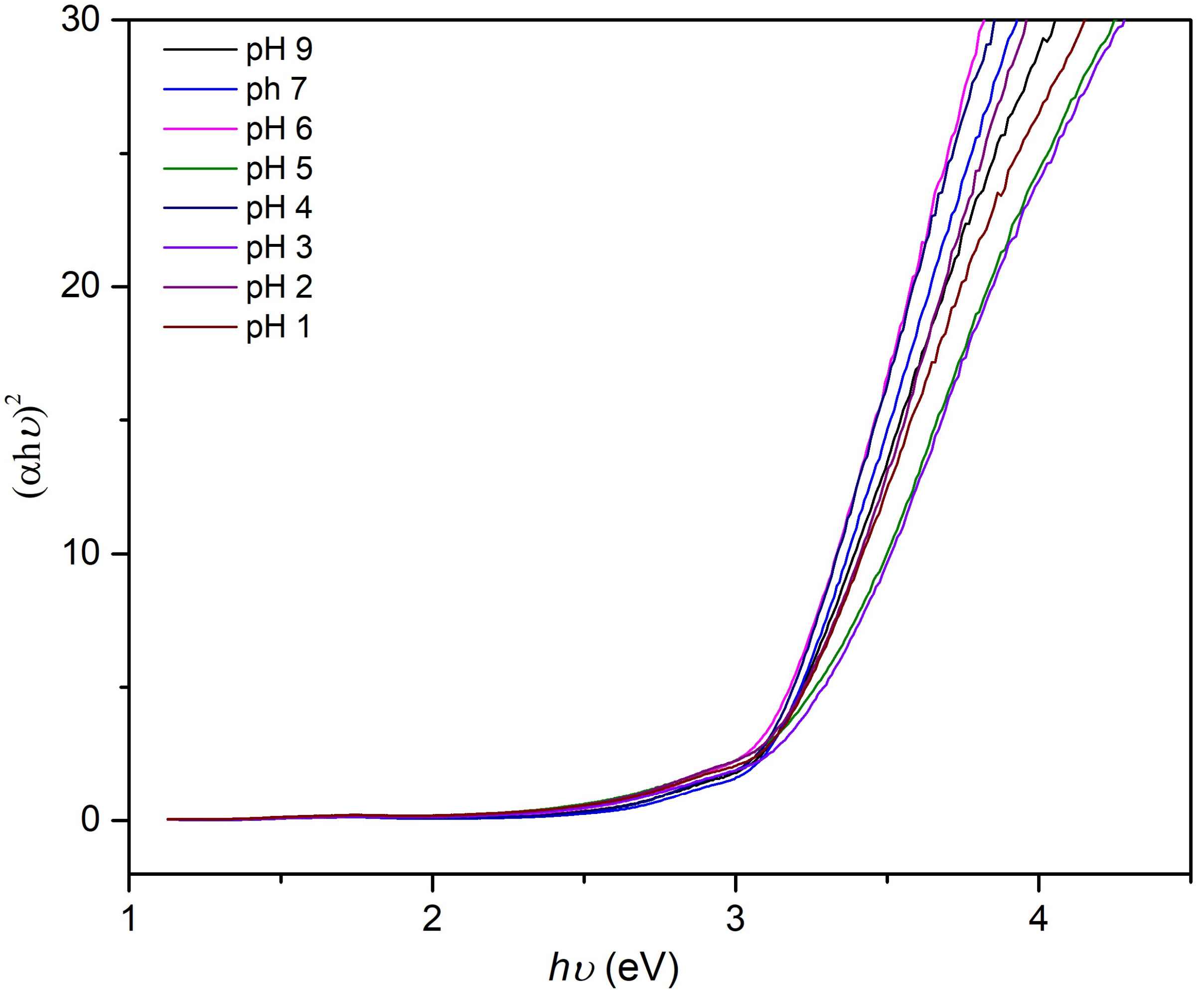

Figure S22. Tauc plots derived from the UV-Vis absorption spectra of $NiMoO_4$ samples produced at different precursor solution's pH (1, 3, 4.57, 5, 6, 7, 9). Calcination temperature: 500 ^{o}C, calcination time: 6 h, and $\varphi = 1$.

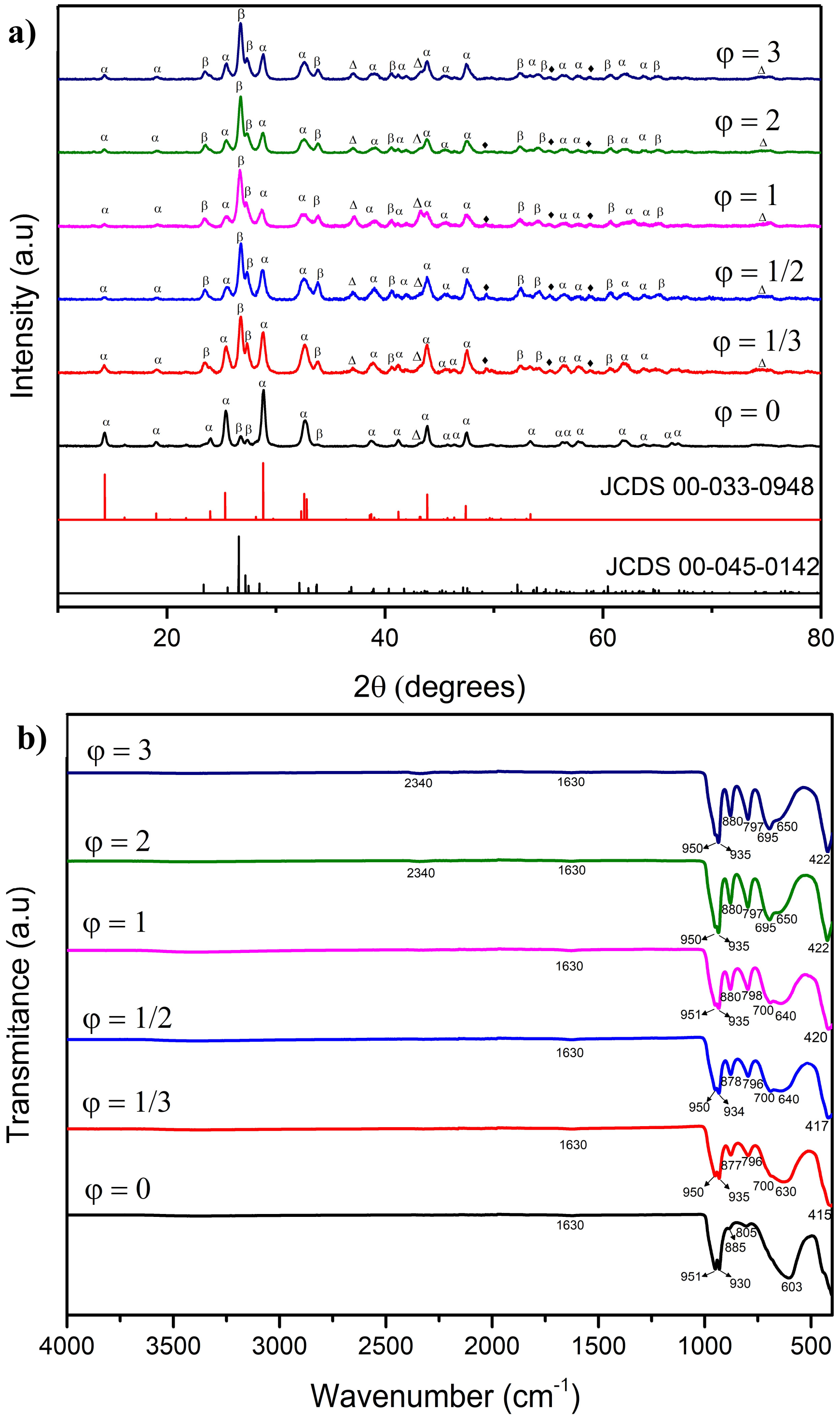

Figure S23. (a) XRD patterns, and (b) FTIR spectra of $NiMoO_4$ samples produced at different fuel/oxidant ratios (φ); (α) α-$NiMoO_4$, (β) β-$NiMoO_4$, (♦) MoO_3, and (Δ) NiO. Calcination temperature: 500 ºC, calcination time: 6 h, and pH = 4.57.

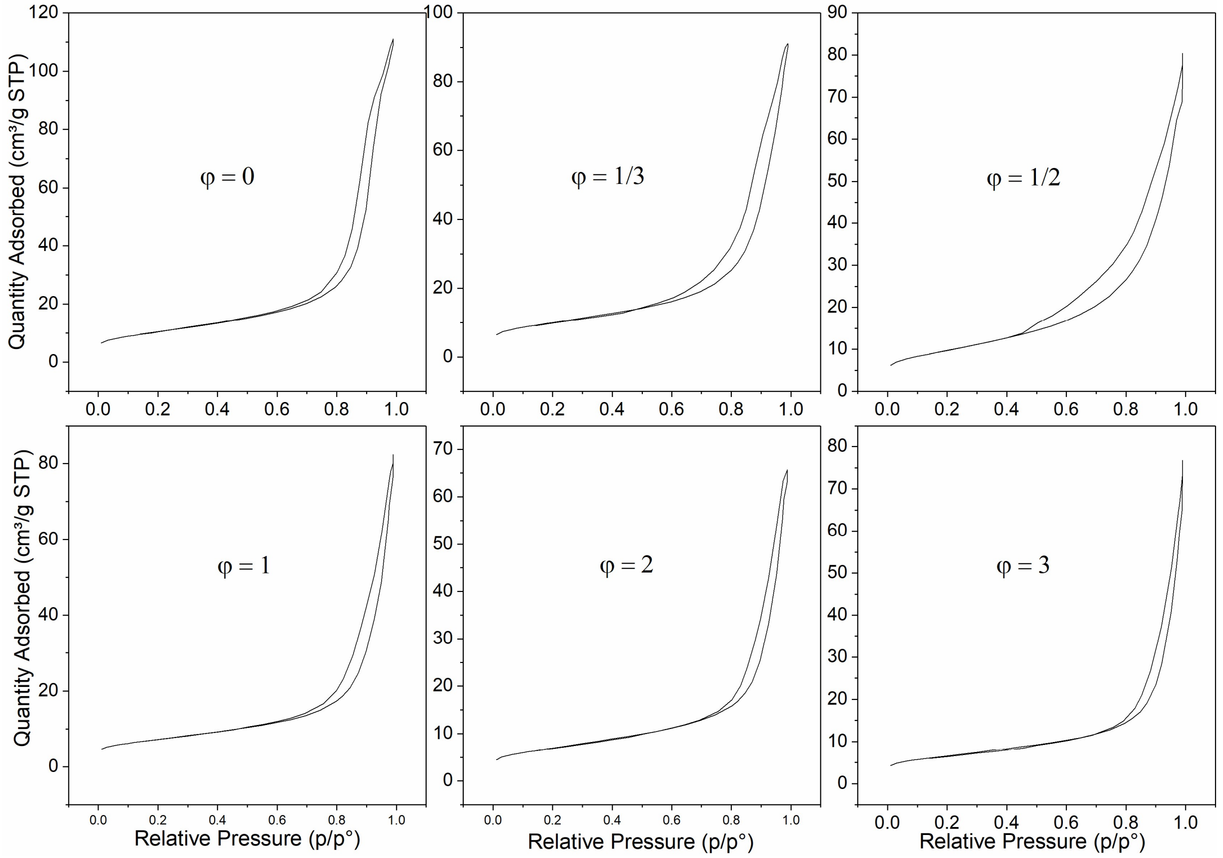

Figure S24. Nitrogen adsorption-desorption isotherms of $NiMoO_4$ samples produced at different fuel-to-oxidant ratios (φ: 0, 1/3, 1/2, 1, 2, 3). Calcination temperature: 500 °C, calcination time: 6 h, and pH = 4.57.

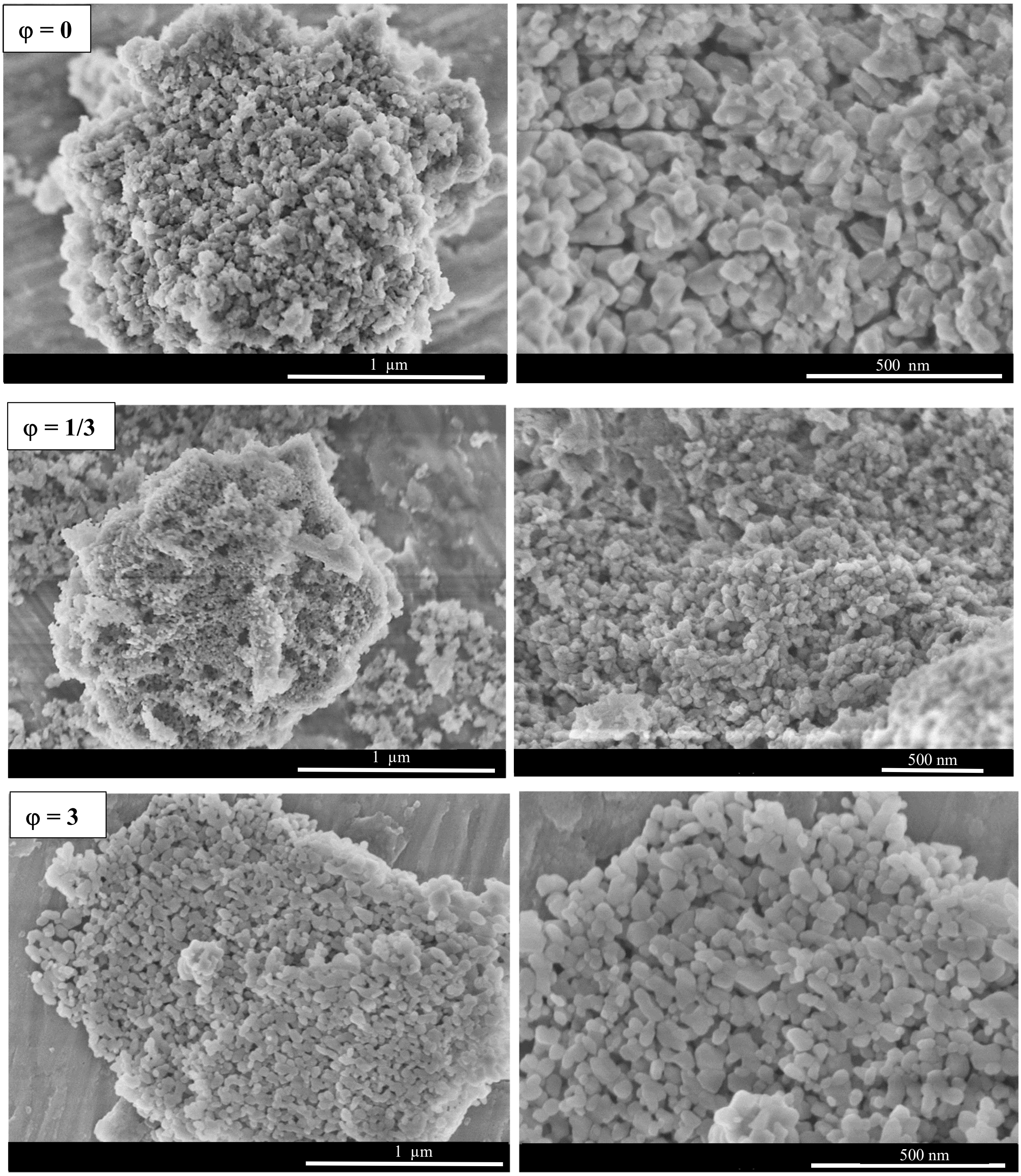

Figure S25. Representative SEM images illustrating the impact of the fuel-to-oxidant ratio (φ: 0, 1/3, 1/2, 1, 2, 3) on the surface morphology of the synthesized $NiMoO_4$ materials. Calcination temperature: 500 °C, calcination time: 6 h, and pH = 4.57.

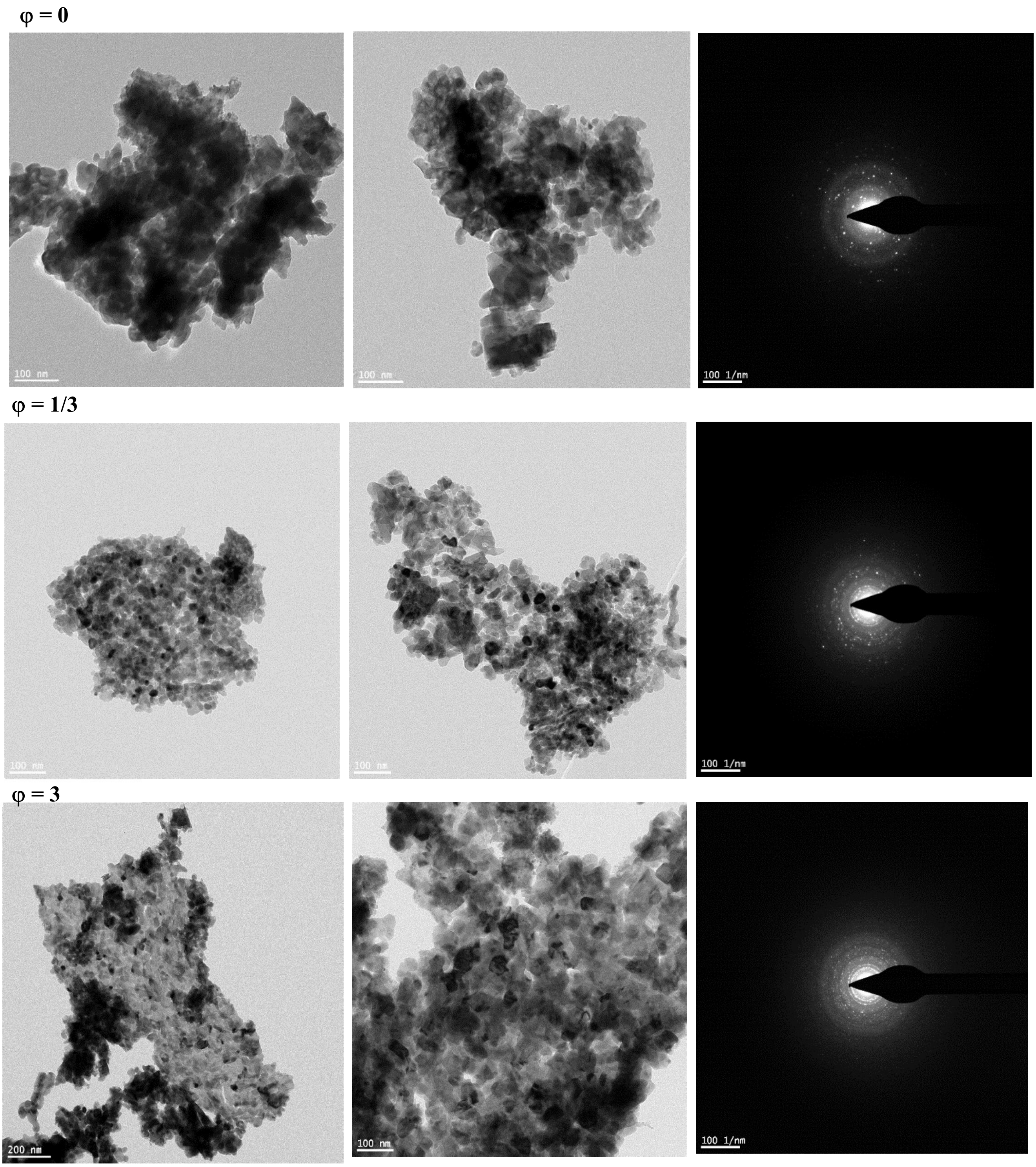

Figure S26. TEM images and SAED patterns of $NiMoO_4$ samples produced at different fuel-to-oxidant ratios (φ: 0, 1/3, 1/2, 1, 2, 3). Calcination temperature: 500 °C, calcination time: 6 h, and pH = 4.57.

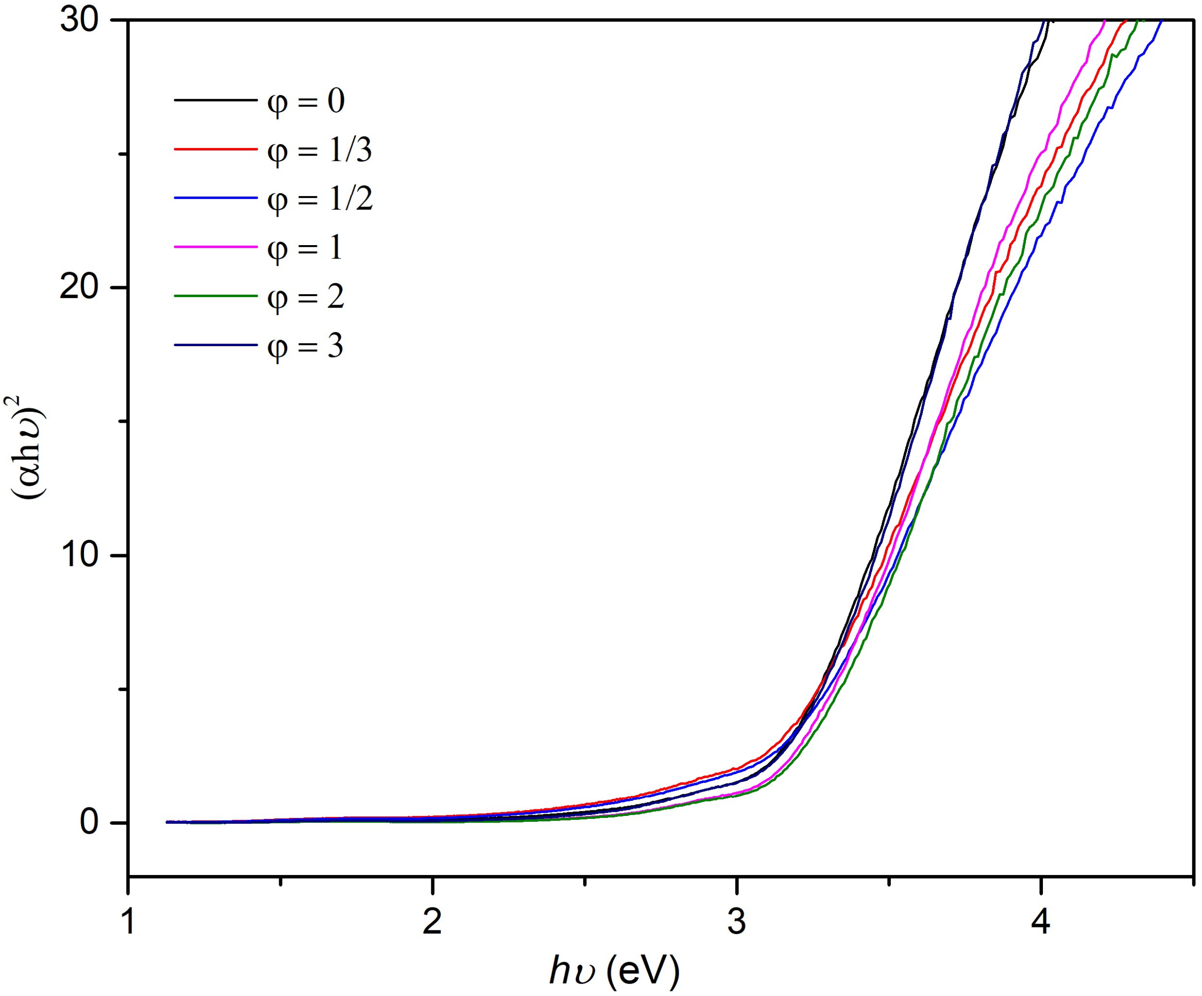

Figure S27. Tauc plots obtained from UV-Vis absorption spectra of $NiMoO_4$ samples produced at different fuel-to-oxidant ratios (φ: 0, 1/3, 1/2, 1, 2, 3). Calcination temperature: 500 ^{o}C, calcination time: 6 h, and pH = 4.57.

1. Varma, A., et al., *Solution combustion synthesis of nanoscale materials.* Chem. Rev., 2016. **116**(23): p. 14493-14586.
2. Rammal, M.B. and S. Omanovic, *Synthesis and characterization of NiO, MoO3, and NiMoO4 nanostructures through a green, facile method and their potential use as electrocatalysts for water splitting.* Mater. Chem. Phys., 2020: p. 123570.
3. Kingsley, J. and L. Pederson, *Combustion synthesis of perovskite LnCrO3 powders using ammonium dichromate.* Mater. Lett., 1993. **18**(1-2): p. 89-96.
4. Morishita, M. and A. Navrotsky, *Calorimetric study of nickel molybdate: Heat capacity, enthalpy, and Gibbs energy of formation.* J. Am. Ceram. Soc., 2003. **86**(11): p. 1927-1932.
5. Cox, J., D. Wagman, and V. Medvedev, *CODATA Key Values for Thermodynamics, Hemisphere Publishing Corp.* New York, 1989.
6. Bates, O.K., *Thermal conductivity of liquid silicones.* Industrial & Engineering Chemistry, 1949. **41**(9): p. 1966-1968.
